# Tumor Cell–Intrinsic c-Myb Upregulation Stimulates Antitumor Immunity in a Murine Colorectal Cancer Model

**DOI:** 10.1158/2326-6066.CIR-22-0912

**Published:** 2023-07-21

**Authors:** Merel van Gogh, Jesus F. Glaus Garzon, Dilara Sahin, Lucia Knopfova, Petr Benes, Onur Boyman, Igor Jurisica, Lubor Borsig

**Affiliations:** 1 Institute of Physiology, University of Zurich, Zurich, Switzerland.; 2 Department of Immunology, University Hospital Zurich, Zurich, Switzerland.; 3 Department of Experimental Biology, Faculty of Science, Masaryk University, Brno, Czech Republic.; 4 International Clinical Research Center, St. Anne's University Hospital, Brno, Czech Republic.; 5 Faculty of Medicine, University of Zurich, Zurich, Switzerland.; 6 Osteoarthritis Research Program, Division of Orthopedic Surgery, Schroeder Arthritis Institute and, Data Science Discovery Centre for Chronic Diseases, Krembil Research Institute, Toronto Western Hospital (UHN), Toronto, Canada.; 7 Department of Medical Biophysics, University of Toronto, Toronto, Canada.; 8 Department of Computer Science, University of Toronto, Toronto, Canada.; 9 Faculty of Dentistry, University of Toronto, Toronto, Canada.; 10 Institute of Neuroimmunology, Slovak Academy of Sciences, Bratislava, Slovakia.; 11 Comprehensive Cancer Center Zurich, University Hospital of Zurich, Zurich, Switzerland.

## Abstract

The transcription factor c-Myb is overexpressed in many different types of solid tumors, including colorectal cancer. However, its exact role in tumorigenesis is unclear. In this study, we show that tumor-intrinsic c-Myb expression in mouse models of colon cancer and melanoma suppresses tumor growth. Although no differences in proliferation, apoptosis, and angiogenesis of tumors were evident in tumors with distinct levels of c-Myb expression, we observed changes in intratumoral immune cell infiltrates. MC38 tumors with upregulated c-Myb expression showed increased numbers of CD103^+^ dendritic cells and eosinophils, but decreased tumor-associated macrophages (TAM). Concomitantly, an increase in the number of activated cytotoxic CD8^+^ T cells upon c-Myb upregulation was observed, which correlated with a pro-inflammatory tumor microenvironment and increased numbers of M1 polarized TAMs. Mechanistically, c-Myb upregulation in immunogenic MC38 colon cancer cells resulted in enhanced expression of immunomodulatory genes, including those encoding β2-microglobulin and IFNβ, and decreased expression of the gene encoding the chemokine receptor CCR2. The increased numbers of activated cytotoxic CD8^+^ T cells contributed to tumor growth attenuation. In poorly immunogenic CT26, LLC, and B16-BL6 tumor cells, c-Myb upregulation did not affect the immunomodulatory gene expression. Despite this, c-Myb upregulation led to reduced B16-BL6 tumor growth but it did not affect tumor growth of CT26 and LLC tumors. Altogether, we postulate that c-Myb functions as a tumor suppressor in a tumor cell–type specific manner and modulates antitumor immunity.

## Teaser

The authors show c-Myb in tumor cells induces expression of immune-related genes, resulting in immunomodulation and tumor growth inhibition. The findings provide a possible mechanistic explanation for the observed better prognosis in cancer patients with higher c-Myb expression.

## Introduction

Colorectal cancer is the fourth most common type of cancer, with a lifetime risk of developing colorectal cancer of 4.1% (1). Although the overall 5-year survival rate has increased from 50% in the 1970s to approximately 70% to date, clinical outcome varies highly due to the heterogeneous nature of the disease.

Transcription is a well-orchestrated process, which is highly controlled in a spatiotemporal manner. Altered expression or activation of transcription factors (TF), due to mutations or protein-fusion, can cause severe cellular dysregulation, which can result in different developmental pathologies, such as autoimmunity, neurological disorders, and cancer (2). In the context of a cancer, TFs are often downstream of multiple signaling pathways. Several TFs, including c-Myc, STAT, and NF-κB, have been identified to play major roles across cancer types (2). Because of the elaborate functions of TFs, many protumorigenic and antitumorigenic processes might be altered.

The *MYB* gene encodes a member of the MYB family of TFs that is restricted to high turnover compartments, such as the hematopoietic system and intestinal epithelial crypts (3). *MYB* is generally considered a proto-oncogene and it is often activated or overexpressed in cancer, including leukemia, colon, and breast cancer. TF c-Myb is a key regulator of stemness in bone marrow, colonic crypts, and in specific regions of the brain (3). Furthermore, hematopoiesis and cell lineage commitment are c-Myb dependent. Many cell type–specific c-Myb target genes are known, and they can influence proliferation, apoptosis, metabolism, inflammation, and angiogenesis (4).

The role of c-Myb in tumorigenesis has mainly been studied in relation to leukemia (5); but its expression has also been linked to solid cancers particularly breast and colon cancer (6, 7). However, reports on the role of c-Myb in solid cancers are contradicting. Initial studies showed that c-Myb acts as an oncogene required for tumorigenesis, and that its enhanced expression correlates with a poor clinical outcome (6, 8). Nevertheless, more recent studies have provided evidence that increased levels of c-Myb correlate with good prognosis for both patients with breast and colorectal cancer (9, 10). Moreover, in a preclinical model, c-Myb expression was shown to inhibit lung metastasis in breast cancer through the suppression of a specific inflammatory signature (11). In addition, c-Myb expression has been suggested to influence the immune compartment in a colon cancer model (12). Thus, whether tumor cell–intrinsic c-Myb expression affects the tumor microenvironment (TME) and antitumor immunity remains an open question.

In this study, we used an inducible c-Myb expression system in several tumor cell lines to elucidate the precise role of c-Myb in tumorigenesis. We found that c-Myb in mouse models of colon cancer and melanoma acts as a tumor suppressor, and that it can shape the immune compartment of the TME, resulting in lasting antitumor immune responses.

## Materials and Methods

### Cell lines

MC38 cells were obtained from Dr. J. Schlom (NIH) in 1997, B16-BL6 melanoma cells were provided by Dr. I. Vlodavsky (Technion Haifa) in 2005, and LLC1.1 (LL/2) and CT26 cells were obtained from ATTC in 2013. All cell lines were cultured in DMEM high glucose containing L-glutamine (Sigma, D5796), supplemented with 10% FBS (Biochrom), S-0615, nonessential amino acids (Thermo Fisher Scientific, 11140), and 1 mmol/L Na-pyruvate (Thermo Fisher Scientific, 11360070), without antibiotics; and kept at 37°C/5%CO_2_ (13). Cells were expanded into low passage number and working stocks frozen down. All cell lines were tested and found to be free of mycoplasma, whereas no further cell authentication assays were carried out. Cells were passaged two to three times post thaw before implanting into mice. c-Myb inducible cell lines were created by lentiviral transduction (Supplementary Fig. S1), followed by sorting of GFP^+^ tumor cells. Selected clones were validated for luciferase activity, GFP expression, and c-Myb induction upon doxycycline (Dox; D-9891; Sigma) 1 μg/mL treatment; detection of c-Myb expression was by immunofluorescence microscopy. We prepared the following TET-on (T/O) tumor cells for use in this study: MC38^T/O^, LLC^T/O^, B16-BL6^T/O^, and CT-26^T/O^.

### Proliferation assay

For 2D culture, 10,000 MC38wt or MC38^T/O^ cells/well were plated (24-well plate) in triplicates and treated with Dox (1 μg/mL) every 24 hours. The number of living cells were counted every 24 hours using Trypan blue and a Neubauer chamber.

For 3D culture. 2,500 cells MC38wt or MC38^T/O^ were plated in spheroid microplates (Corning, CLS4515) in triplicates and treated with Dox (1 μg/mL) every 24 hours. In addition, every 24 hours images were taken, and the maximum diameter of the spheroids was determined using the Leica DMi8 Thunder Imager.

### Cloning

The plasmids used for cloning were bought from Addgene (Supplementary Table S1). The c-Myb transcript was cloned into Entry vector #62099 downstream of the tetracycline (TET) inducible CMV promoter using *Bam*HI and *Xba*I sites. Two-fragment recombination was performed as described previously (14). Briefly, the recombination was performed in one step by adding 5 fmoles of each entry vector to 10 fmoles of the destination vector, 2.5 μL of TE buffer and Gateway LR CLonease II Plus Enzyme Mix (Life Technologies, #12538–120). After an incubation of 16 to 20 hours at 25°C and a 10 minute proteinase K digestion at 37°C, DH5α competent cells (Thermo Fisher Scientific, #18265017) were transformed. For validation of the two-fragment recombination, single colonies were expanded, analyzed for the correct digestion pattern, and sequenced (Microsynth). We used the same vector construct containing a tetracycline-inducible *MYB* region to create T/O tumor cells (Supplementary Fig. S1A).

### IHC staining and microscopy

For c-Myb quantification, Dox inducible cells were cultured on chamber slides (Thermo Fisher Scientific) and treated with Dox (1 μg/mL) for the indicated times. After fixation with 2% paraformaldehyde for 15 minutes, and 30 minutes incubation with 20 mmol/L glycine, slides were blocked with 1%BSA/0.15%Tween/PBS for 1 hour. Cells were stained with rabbit anti–c-Myb (Abcam, ab226470), counterstained with 4′,6-diamidino-2-phenylindole (DAPI; Sigma, D-9542), and slides were mounted using Prolong Gold Antifade (Invitrogen). Images were taken with a Leica DMi8 Microscope (40× lens, DFC 9000GT camera), using Thunder Imaging System and were quantified using LASX Analysis Software, v.4.4.1, where a mask for the DAPI staining (nuclei) was created and c-Myb mean intensity was measured.

For IHC and immunofluorescence (IF) staining, 5 μm formalin-fixed paraffin embedded tumor tissues sections were stained with antibodies specific for c-Myb (Abcam, ab226470), Ki67 (eBioscience, SolA15), cCasp3 (Cell Signaling Technology, 9661), CD31 (Abcam, ab28364), and CD8 (Novus Biologicals, NBP1–50069), and then counterstained with DAPI (IF) or hematoxylin (Sigma, MHS16; IHC). For c-Myb and Ki67 staining, antigen retrieval was performed using Antigen Unmasking Solution, Citrate-Based (Vectorlabs, H-3300) before staining. Staining for cCasp3, CD31, and CD8 was performed on a NEXES IHC robot (Ventana instruments) using an iVIEW DAB Detection Kit (Roche Diagnostics, 05266157001) or on a Bond MAX (Leica). For IF staining of OCT Tissue-Tek (Sakura, 4583)-embedded frozen tumor tissues (obtained from murine tumors of all used cell lines), 5 μm cryosections were stained for CD31 (BioLegend, MEC13.3) and counterstained with DAPI. Histology samples were scanned using a Leica DMi8 Thunder Imager and quantified with LASX Analysis Software. IHC analysis was normalized per tumor area and vessel perfusion was quantified as percentage of CD31^+^ vessels.

### Luciferase assay

Dox inducible cell lines were incubated with Luciferin (Perkin Elmer, 122799) for 10 minutes at 37°C. Luciferase activity was analyzed by IVIS Spectrum Imaging (Zurich Integrative Rodent Physiology, University of Zurich).

Wild-type MC38 cells were transiently transfected with both tetracycline inducible c-Myb–eGFP–Neomycin and a c-Myb reporter plasmid containing luciferase (15) using Lipofectamine 3000 (Thermo Fisher Scientific). The luciferase assay was performed 48 hours after transfection by following the manufacturer's instructions (Promega), after Dox (1 μg/mL) treatment for the indicated time points.

### Animal experiments

All animal experiments were approved by the Veterinary Office of Kanton Zurich, Switzerland, and were performed according to the guidelines of the Swiss Animal Protection Law. Wild-type C57BL/6J mice (male and female, 6 to 8 weeks old) were purchased from Charles River Laboratories. RAG2γc-deficient, RAG1-deficient, and Batf3-deficient mice were kindly provided by Dr. Schneider (University of Zurich). Mice were housed in specific pathogen free (SPF) conditions on a 12-hour light/dark cycle with chow/water provided *ad libitum*. Mice were randomly assigned to experimental groups.

LLC1.1^T/O^ (3 × 10^5^ cells), wtMC38 or MC38^T/O^ (5 × 10^5^ cells), B16-BL6^T/O^ (1 × 10^6^ cells), or CT26^T/O^ (5 × 10^5^ cells) tumor cells were subcutaneously injected in the right flank of a mouse. Tumor size was measured three times weekly from the second week onwards using calipers and tumor volume was calculated: *V* = *π* × *L* × *W*^2^/6. Mice were sacrificed at the defined endpoint, or when tumors became necrotic or reached 2 cm^3^. Mice were given control or Dox (800 mg/kg) containing chow (Granovit AG) starting the day after tumor cell injection, unless stated otherwise.

To measure tumor cell proliferation *in vivo*, EdU (Invitrogen, A10044; 50 mg/kg body weight) was intraperitoneally injected 20 hours before tumor cell analysis by flow cytometry. For tissue perfusion, 100 μL fluorescently-labeled Lycopersicon Esculentum Lectin (Vector Lab, TL-1176–1) was intravenously injected 10 minutes before termination. Mice were anesthetized with 10% ketamine 0.65mL/kg and 2% xylazine 0.5 mL/kg prior to perfusion with 2% paraformaldehyde. Tumors were dissected, embedded in OCT Tissue-Tek (Sakura, 4583), and analyzed by IF.

### Flow cytometry and cell sorting

To analyze apoptosis, cells were stained with the Annexin V Apoptosis Detection Kit with 7-AAD (BioLegend, 640922) by following the manufacturer's instructions. Data were acquired with the BD FACSCanto (3L) and analyzed using FlowJo software v.7.6.5 (TreeStar).

Flow cytometry analysis of tumor tissue was conducted after mice were perfused with PBS and dissected tumors were digested with Collagenase IV (Sigma, C5138), DNase I (Sigma, DN25), and Hyaluronidase (Sigma, H6254) in RPMI1640 (Gibco, 52400) containing 2% FBS for 30 minutes at 37°C. The cell suspension was filtered through a 100 μm cell strainer, erythrocytes lysed with ammonium–chloride–potassium lysis buffer, and the resulting suspension filtered through a 40 μm cell strainer. Single cell suspension was stained with Zombie Fixable Viability Kit (BioLegend, 423114) for 30 minutes on ice, incubated with anti-CD16/32 (BD, 553141) for 10 minutes in FACS buffer (PBS/10 mmol/L EDTA/2% FBS), and then incubated with antibodies for surface antigens (Supplementary Table S2) for 30 minutes. For intracellular staining, samples were fixed and permeabilized using the Foxp3/TF Staining Set (Thermo Fisher Scientific, 00–5523) and subsequently incubated with antibodies for intracellular proteins for 30 minutes (Supplementary Table S2). Data were acquired with a Cytek Aurora (5L) Spectral Analyzer in the Flow Cytometry Facility at the University of Zurich and analyzed using FlowJo software v.7.6.5.

For EdU flow cytometry analysis, tumor samples were prepared as described above and the Click-iT Plus EdU Flow Cytometry Assay Kit (Thermo Fisher Scientific, C10634) was used according to the manufacturer's instructions.

GFP^+^ tumor cells were sorted from tumor samples that were prepared as described above and stained with Zombie Fixable Viability Kit for 30 minutes on ice, incubated with anti-CD16/32 for 10 minutes in FACS buffer, and then incubated with anti-CD45 for 30 minutes. At least 500,000 GFP^+^CD45^−^ cells were sorted with the BD FACSAria III (5L; Flow Cytometry Facility, University of Zurich) using the 100 μm nozzle and processed for RNA extraction (see RNA isolation and qRT-PCR).

### Western blotting

Murine (T/O) tumor cells were treated with Dox as indicated and lysed with lysis buffer (20 mmol/L Tris, 150 mmol/L NaCl, 1 mmol/L EDTA, 1% Triton X-100), supplemented with Phosphatase Inhibitor Cocktail (Sigma, P0044) and cOmplete Protease Inhibitor Cocktail (Roche, 11873580001) for 20 minutes on ice and cleared by centrifugation for 10 minutes at 14,000 × *g*. Cell lysates were separated on 10% SDS-PAGE gels and blotted on protran nitrocellulose membranes (GE Healthcare). Membranes were blocked with 5% milk in TBS-Tween and overnight incubated with anti-Myb antibody (1:100; Catalog No. 05–175, Millipore), followed by a 1 hour incubation with a secondary HRP-conjugated antibody (Cell Signaling technology, 7076) and chemiluminescent detection using Amersham Hyperfilm ECL (GE Healthcare). Subsequent incubation and detection of β-actin (Cell Signaling Technology, 3700) was used as a loading control. Quantification was performed using ImageJ (Fiji).

### Cytokine and chemokine analysis

Whole tumor tissues were sonicated in PBS supplemented with cOmplete Protease Inhibitor Cocktail (Roche) and centrifuged for 10 minutes at 16,000 × *g*. Supernatant was used for Bio-Plex analysis using the Bio-Plex Pro Mouse Chemokine Panel 31-Plex and Bio-Plex Pro Mouse Cytokine 23-Plex kits (Bio-Rad, 12009159 and M60009RDPD, respectively) and analyzed with the Bio-Plex 200 Systems (Bio-Rad). Protein concentration was determined by using the Pierce BCA Protein Assay Kit (Thermo Fisher Scientific, 23227).

### RNA isolation and qRT-PCR

RNA was extracted from cells cultured *in vitro* or sorted tumor cells using the Direct-zol RNA miniprep (Zymo Research, R2070) or RNeasy Plus Mini Kit (Qiagen, 74034), respectively. cDNA was synthesized using the Omniscript RT Kit based on 200 ng of total RNA (Qiagen, 2051511) and qPCR was performed with KAPA SYBR FAST qPCR Master Mix (KAPA Biosystems, KK4602) using specific primers (Supplementary Table S2), 1 μL of a template (1:100 dilution) in duplicates, and it was analyzed with a CFX96 Touch Real-Time PCR (Bio-Rad) using 2^–ΔΔCT^. Gene expression was normalized against the house-keeping gene *Gapdh* and relative to the control or untreated samples.

### RNA sequencing

RNA was extracted from sorted tumor cells (GFP^+^CD45^−^) using the RNeasy Plus Mini Kit (Qiagen, 74034). RNA quality control, library preparation, sequencing, and data pre-processing were performed by Lexogen GmbH, as described previously (16). The RNA sequencing (RNA-seq) data have been deposited in gene expression omnibus (GEO) under the accession number GSE232823.

### Alignment and read quantification

The reads were aligned to the spike-in complemented Ensembl release 101 of the Mus musculus high coverage assembly GRCm38 from the Genome Reference Consortium. The alignment was performed with the splice-aware aligner STAR version 2.6.1a (17). The alignments were quantified based on the annotations of Ensembl GRCm38 and the spike-in specific annotations of Lexogen with the FeatureCounts software program version 1.6.4 of the subread analysis. A differential gene expression analysis was conducted using DESeq2 version v1.18.1 (18). Significance was determined at adjusted *P* < 0.05. Differentially expressed transcripts were clustered using Functional Genomics Center Zürich Heatmap tool (http://fgcz-shiny.uzh.ch).

### Network and enrichment analyses

Gene Ontology enrichment analysis was performed using Enrichr tool (https://maayanlab.cloud/Enrichr) and the 2021 version of the Gene Ontology. Enrichment results for biological process, molecular function, and cellular compartment are sorted based on *P* value.

Pathway enrichment analysis was performed using integrated pathway database portal pathDIP (19) version 4.1 (http://ophid.utoronto.ca/pathDIP). A total of 87 differentially expressed genes (DEG) were submitted as input for querying pathDIP, selecting all database sources, extended pathways, and using default parameters. Identified significantly enriched pathways are presented in Supplementary Table S3. The most relevant pathways, that is, significantly enriched pathways that covered 35 or more of the input DEGs present (top 10% of significant pathways) were further considered and are listed in Supplementary Table S4.

### Killing assay

TET-on MC38^T/O^, LLC^T/O^, and B16-BL6^T/O^ tumor cells were cultured in 48-well plates overnight before a 24-hour treatment with Dox (1 μg/mL). Subsequently CD8^+^ T cells were enriched from spleens of control or Dox-treated tumor-bearing mice using the MojoSort Mouse CD8 T-Cell Isolation Kit (BioLegend, 480008). Cultured tumor cells and enriched CD8^+^ T cells were added to the culture in ratios 1:2 or 1:5 (tumor cell:T cell). After 24 hours, samples were analyzed for apoptotic tumor cells by flow cytometry.

### Survival analysis

Dataset GSE39582 containing mRNA expression profiling of 562 patients with colorectal cancer was retrieved from Gene Expression Omnibus (20). Kaplan–Meier plots representing the probability of overall survival (OS) of patients with colorectal cancer stratified according to the median expression of MYB (probe ID 204798_at) were calculated with GraphPad Prism software (version 9.2.0). The log-rank test was used to assess the significance of the association with survival outcome.

### Statistical analysis

Data are presented as mean values ± SEM. Statistical analysis was performed using the GraphPad Prism software (version 9.2.0). Mann–Whitney test or one-way ANOVA with Kruskal–Wallis posttest were used when comparing two or more groups, respectively, and two-way ANOVA was used for comparing tumor growth curves.

### Data availability

The RNA-seq data have been deposited in GEO under the accession number GSE232823. All other data are available in the main text and the Supplementary Materials and Methods or are available from the corresponding author upon reasonable request.

## Results

### c-Myb upregulation in tumor cells can inhibit tumor growth *in vivo*

**Figure 1.**
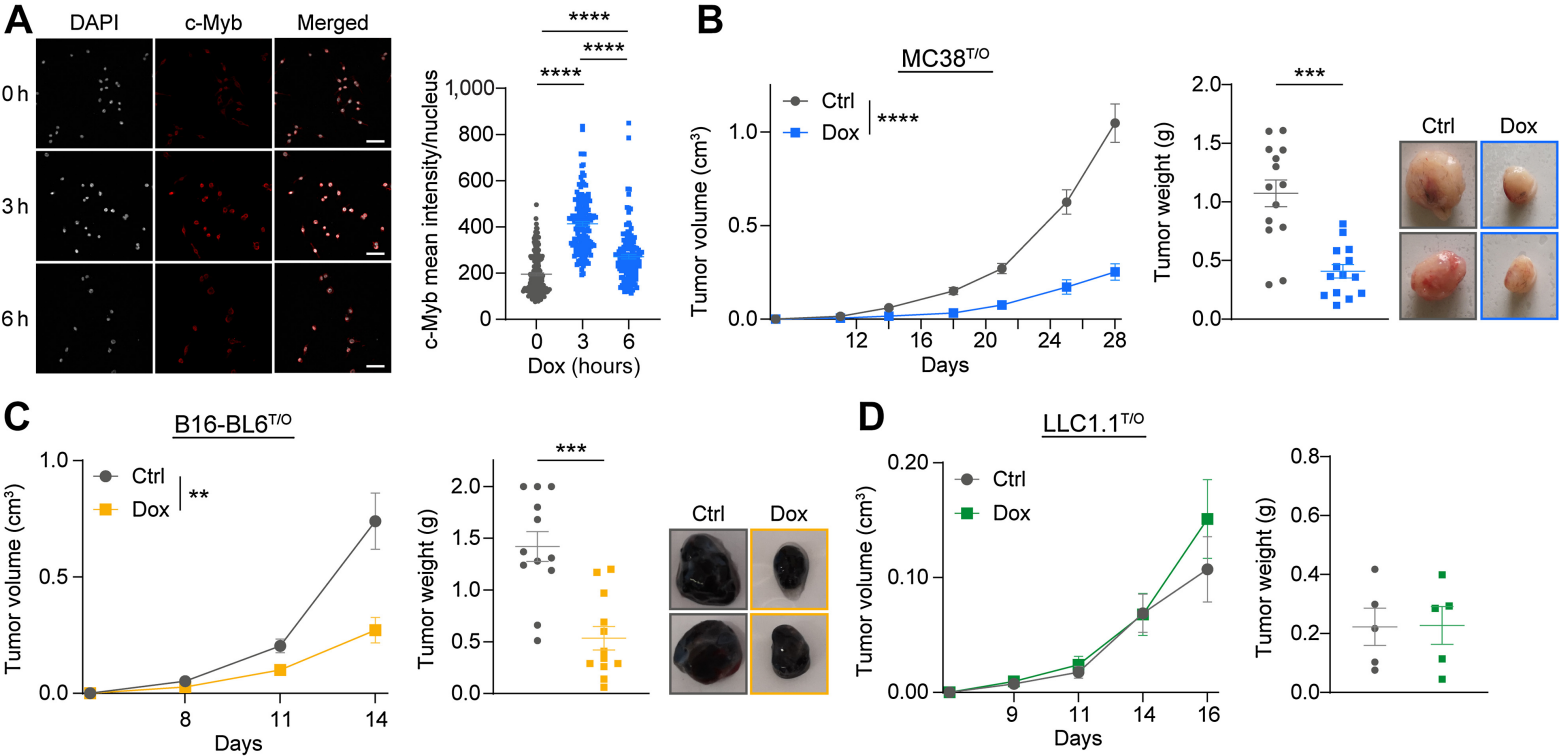
c-Myb upregulation reduces MC38 and B16-BL6 tumor growth *in vivo*. **A,** Immunofluorescence staining of c-Myb (red) in MC38^T/O^ cells, treated for the indicated times with 1 μg/mL Dox *in vitro*. c-Myb staining is quantified as mean intensity per nucleus (white). Data are presented from two independent experiments, in duplicates. Each data point represents one cell. Scale bar, 50 μm. **B–D,** C57BL/6J mice were subcutaneously injected with 5 × 10^5^ MC38^T/O^ (**B**), 1 × 10^6^ B16-BL6^T/O^ (**C**), or 3 × 10^5^ LLC^T/O^ (**D**) cells and were fed control (Ctrl) or Dox containing chow for the duration of the experiment. Tumor growth, final tumor weight, and representative pictures are shown. *n* = 14 (**B**), *n* = 12 (**C**), or *n* = 5 (**D**) mice per group from three (**A**), two (**B**), or one (**C**) independent experiments. Data are presented as mean values ± SEM. One-way ANOVA with Kruskal–Wallis posttest was used for statistical analysis (**A**), two-way ANOVA was used for comparing tumor growth curves (**B–D**). **, *P* < 0.01, ***, *P* < 0.001, ****, *P* < 0.0001.

To investigate the role of c-Myb in solid tumors, we developed an inducible c-Myb system in several murine cancer cell lines [MC38 colon cancer cell line, B16-BL6 melanoma cell line, and Lewis lung carcinoma (LLC) cell line]. This system allowed us to elucidate the role of c-Myb at different stages of tumor development. MC38^T/O^ cells treated with Dox *in vitro* showed a time-dependent increase in c-Myb mRNA and protein expression (Supplementary Fig. S1B). Similarly, Dox treatment of MC38^T/O^, B16-BL6^T/O^, and LLC^T/O^ cells induced an upregulation of c-Myb protein as determined by its nuclear localization using IF (Fig. 1A; Supplementary Figs. S1C and S1D). To confirm that c-Myb was biologically active upon Dox treatment, we transiently transfected a c-Myb reporter plasmid with luciferase, which showed increased luciferase activity (Supplementary Fig. S1E). Finally, we quantified the expression of known c-Myb target genes 6 and 24 hours after Dox stimulation of MC38^T/O^ cells (Supplementary Fig. S1F). Induced expression of *Cited2*, *Rad54b*, *Daam1*, and *Calm2* genes and reduced expression of *Rab31* and *Lcp1* target genes (21) was detected.

Because the TF c-Myb is generally known as a proto-oncogene, we expected an increase in tumor growth upon subcutaneous tumor cell injection in C57BL/6J mice fed Dox containing chow. However, we observed a significant decrease in tumor growth of both MC38^T/O^ and B16-BL6^T/O^ tumors in Dox-treated mice compared with mice receiving control (Ctrl) chow (Fig. 1B and C; Supplementary Fig. S1G). In contrast, no significant changes in LLC^T/O^ tumor growth upon Dox treatment were detected (Fig. 1D). We confirmed the upregulation of c-Myb in tumors of Dox treated mice (Supplementary Fig. S1H). To ascertain that the observed phenotype is not induced only by Dox treatment, but was a result of c-Myb upregulation, we repeated the *in vivo* experiment using wild-type (wt) MC38 tumor cells (Supplementary Fig. S1I). In addition, no difference was observed in wtMC38 subcutaneous tumor growth between Ctrl- and Dox-treated mice. These data show that c-Myb upregulation strongly inhibits tumor growth in mouse models of colon cancer and melanoma.

### c-Myb upregulation in MC38 tumor cells has no effect on cell proliferation, apoptosis, or tumor angiogenesis

**Figure 2.**
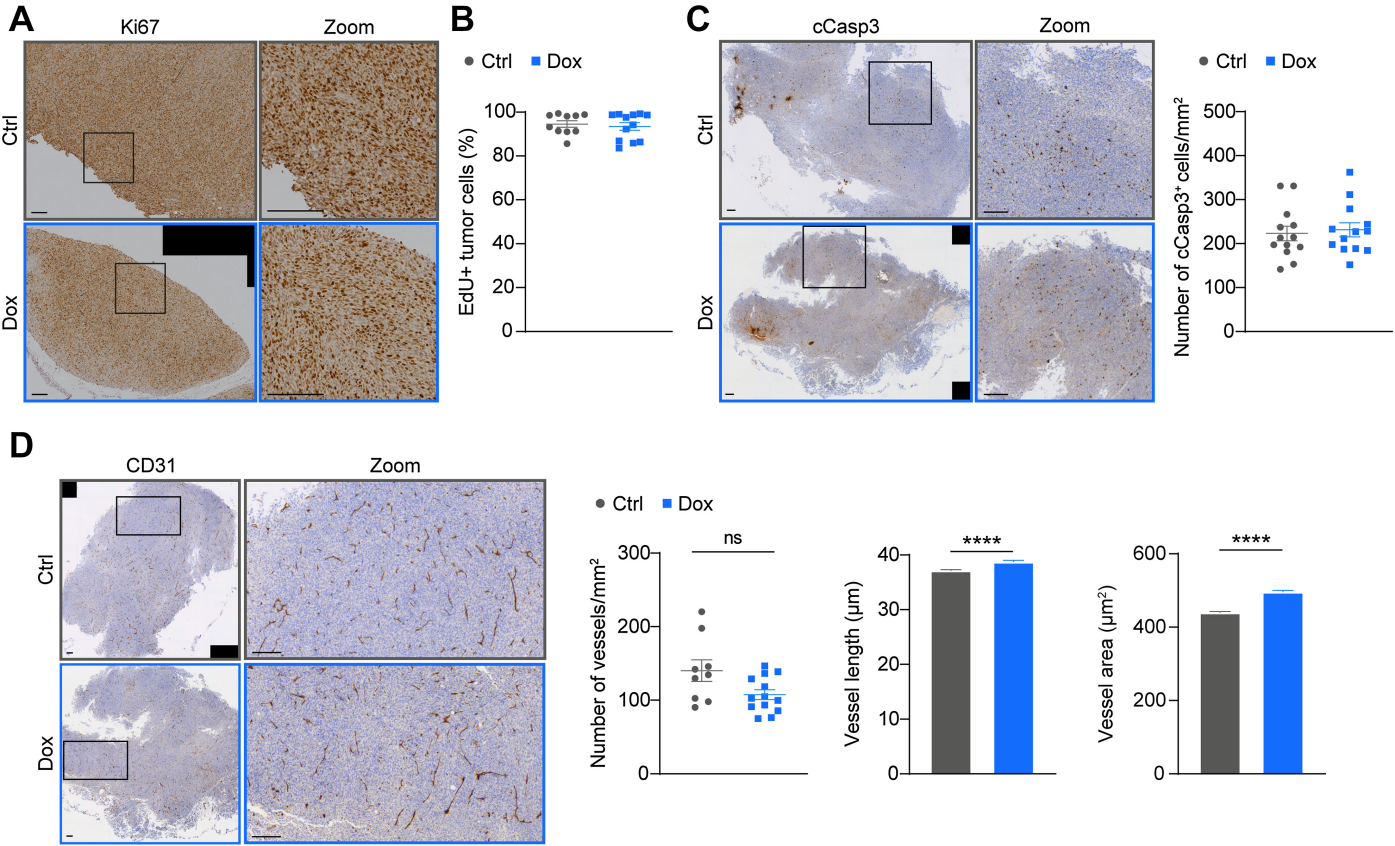
c-Myb upregulation does not alter MC38 intrinsic properties or tumor angiogenesis. **A, C,** and **D,** Representative images of Ki67 (**A**), cleaved caspase 3 (cCasp3; **C,** left) and CD31 (**D,** left) stained formalin-fixed paraffin embedded MC38^T/O^ subcutaneous tumor sections from control (Ctrl) or Dox-treated mice. Quantifications of cCasp3 and CD31 are shown as number of positive cells / vessels per mm^2^ (**C** and **D,** right) and mean vessel length and area (**D,** right), respectively. **B,** Proliferating GFP^+^EdU^+^ tumor cells are represented as percentage of total living GFP^+^ tumor cells as assessed by flow cytometry of MC38^T/O^ sc tumors from Ctrl or Dox-treated mice. *n* = 9–13 mice per group from two (**B**) or three (**A, C,** and **D**) independent experiments. Data are presented as mean values ± SEM. Mann–Whitney test was used for statistical analysis. ns, not significant; ****, *P* < 0.0001. Scale bars, 200 μm.

c-Myb is known to play a role in several autocrine signaling pathways, such as proliferation and apoptosis (4). Thus, we hypothesized that the observed reduction in tumor growth upon c-Myb upregulation might be due to tumor cell intrinsic pathways. The analysis of proliferation in both 2D and 3D culture did not show any differences between untreated and Dox-treated MC38^T/O^ cells (Supplementary Figs. S2A and S2B). Moreover, proliferation did not change in subcutaneous MC38^T/O^ tumors upon Dox treatment, as determined by staining of the proliferation marker Ki67 and the incorporation of the thymidine analog EdU *in vivo* (Fig. 2A and B). Analysis of apoptotic cells showed no significant differences between untreated and Dox treated MC38^T/O^ cells *in vitro* (Supplementary Fig. S2C). Similarly, staining of cleaved Caspase 3 (cCasp3) in subcutaneous MC38^T/O^ tumors from Ctrl and Dox treated mice, did not reveal any significant differences in numbers of apoptotic cells (Fig. 2C).

Because c-Myb activity is known to influence angiogenesis (4), we assessed tumor blood vessels. CD31 staining of blood vessels in subcutaneous MC38^T/O^ tumors from Ctrl and Dox-treated mice and showed no apparent difference in number of vessels; however, there was a small, but statistically significant increase in vessel length and area in Dox-treated mice (Fig. 2D). The functionality of the perfused blood vessels showed no significant differences between Ctrl and Dox-treated mice (Supplementary Fig. S2D).

### c-Myb upregulation by tumor cells drives changes in the TME

**Figure 3.**
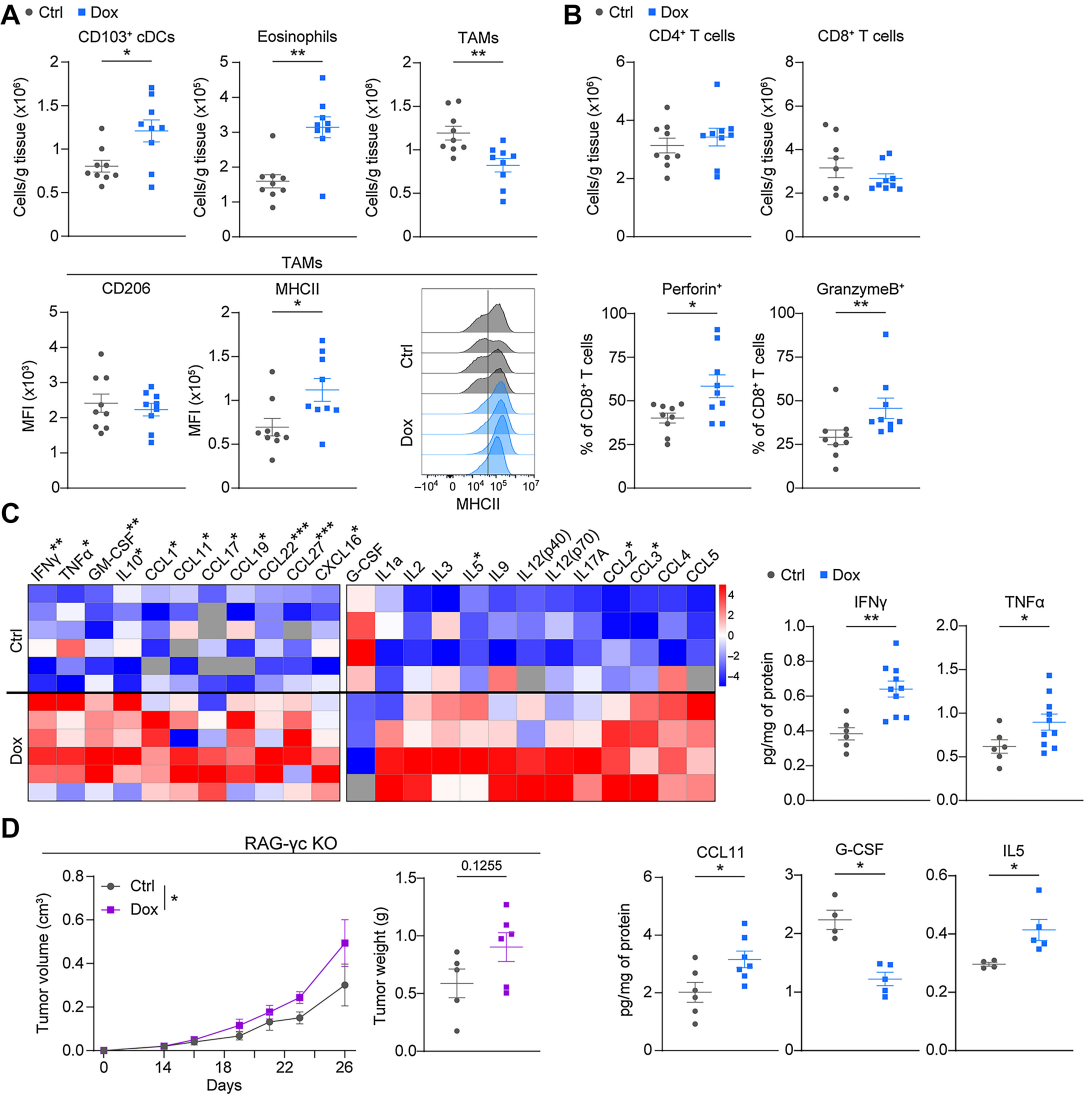
Tumor cell c-Myb upregulation results in changes in the TME. **A** and **B,** Flow cytometry analysis of subcutaneous MC38^T/O^ tumors from Ctrl or Dox-treated mice at day 28 for CD103^+^ conventional DC (cDC); CD45^+^CD64^−^CD11c^+^MHCII^+^CD103^+^), eosinophils (CD45^+^CD11b^+^Ly6G^−^MHCII^−^SiglecF^+^), TAMs (CD45^+^CD11b^+^CD64^+^CD11c^+^), and CD4^+^/CD8^+^ T cells (CD45^+^CD11b^−^CD3e^+^CD4^+^ or CD8^+^, respectively). Data are represented as the number of cells per gram of tumor tissue. Median fluorescent intensity (MFI) is shown for CD206 and MHCII on TAMs, as well as representative histograms for MHCII expression. CD8^+^ perforin^+^/granzymeB^+^ populations are represented as percentage of total CD8^+^ T cells. **C,** Bioplex analysis of subcutaneous MC38^T/O^ tumor homogenates of Ctrl and Dox-treated mice using two separate detection kits. Heatmaps are shown of representative samples. Graphs of absolute values are represented as pg of cytokine per mg of total protein. **D,** Tumor growth curves and final tumor weight of Ctrl or Dox-treated RAG-γc deficient (KO) mice subcutaneously injected with MC38^T/O^ cells. *n* = 9–12 (**A** and **B**), *n* = 4–10 (**C**), or *n* = 5–6 (**D**) mice per group, from two independent experiments (**A–C**). Data are presented as mean values ± SEM. Mann–Whitney test was used (**A–C**) for statistical analysis. Two-way ANOVA was used for comparing tumor growth curves (**D**). *, *P* < 0.05, **, *P* < 0.01, ***, *P* < 0.001.

To assess changes in the TME, we analyzed immune cells present in subcutaneous MC38^T/O^ tumors from Ctrl and Dox treated mice (Fig. 3A and B; Supplementary Figs. S3A–S3C). We detected a significant increase in CD103^+^ conventional dendritic cells (cDC) in tumors from Dox-treated mice (Fig. 3A), whereas no changes in Ly6C^+^ monocytic cells were found (Supplementary Fig. S3C). An increase in eosinophils and a concomitant decrease in Ly6G^+^ granulocytic cells was also observed (Fig. 3A; Supplementary Fig. S3C). We observed a decrease in CD45^+^CD11b^+^CD64^+^CD11c^+^ cells, hereafter named tumor-associated macrophages (TAM), in tumors from Dox-treated mice (Fig. 3A). Macrophages are often divided into two main categories, antitumorigenic, MHC-II–expressing antigen-presenting macrophages, and protumorigenic macrophages characterized by CD206 expression (22). TAMs from subcutaneous MC38^T/O^ tumors of Dox-treated mice showed a homogenous population with high MHC-II expression (Fig. 3A). The expression of CD206 in TAMs was overall low regardless of treatment. The analysis of adaptive immune cells revealed no differences in counts of CD4^+^ and CD8^+^ T cells (Fig. 3B; Supplementary Fig. S3B). However, percentages of CD8^+^ T cells expressing perforin or granzyme-B were significantly increased in subcutaneous MC38^T/O^ tumors from Dox-treated mice. No change in PD1 expression was observed (Supplementary Fig. S3C). A similar phenotype of CD8^+^ T cells was observed in subcutaneous B16-BL6^T/O^ tumors from Dox-treated mice (Supplementary Fig. S3D), where we also observed an increase in total numbers of intratumoral CD4^+^ and CD8^+^ T cells.

Cytokine analysis of MC38^T/O^ tumors of Ctrl and Dox-treated mice showed striking differences (Fig. 3C; Supplementary Figs. S3E and S3F). Many classical pro-inflammatory cytokines, including IFNγ and TNFα, were more abundantly present in tumors of Dox-treated mice, in line with the enhanced activity of the CD8^+^ T cells (Fig. 3B). In addition, CCL11 and IL5, which are known to stimulate eosinophils, were increased in tumors of Dox-treated mice, in line with the increased intratumoral numbers of eosinophils (Fig. 3A). In contrast, G-CSF levels were reduced in Dox-treated mice, consistent with decreased counts of granulocytes in tumors (Fig. 3C; Supplementary Fig. S3C). In addition, IFNγ-driven increased expression of chemokines in tumors of Dox-treated mice (Fig. 3C; Supplementary Fig. S3F), such as CCL2, CCL3, CCL5, and CXCL16 has been associated with enhanced recruitment of CD8^+^ T cells to tumors and reduced tumor progression (23–25). Collectively, the upregulation of c-Myb in two different cancer models (MC38 and B16-BL6) induces changes in the tumor immune compartment.

### Tumor control upon c-Myb upregulation is dependent on the adaptive immune system

To test whether tumor control upon c-Myb upregulation was mediated by the adaptive immune system, we injected MC38^T/O^ cells subcutaneously in severely immune-deficient RAG2γc-deficient mice, which lack innate lymphoid, T, B, and NK cells. We observed a significant increase in tumor growth upon c-Myb upregulation in these mice (Fig. 3D). We repeated the experiment in RAG1-deficient mice, where we found a similar phenotype (Supplementary Fig. S3G).

### Early c-Myb upregulation is crucial for control of tumor growth

**Figure 4.**
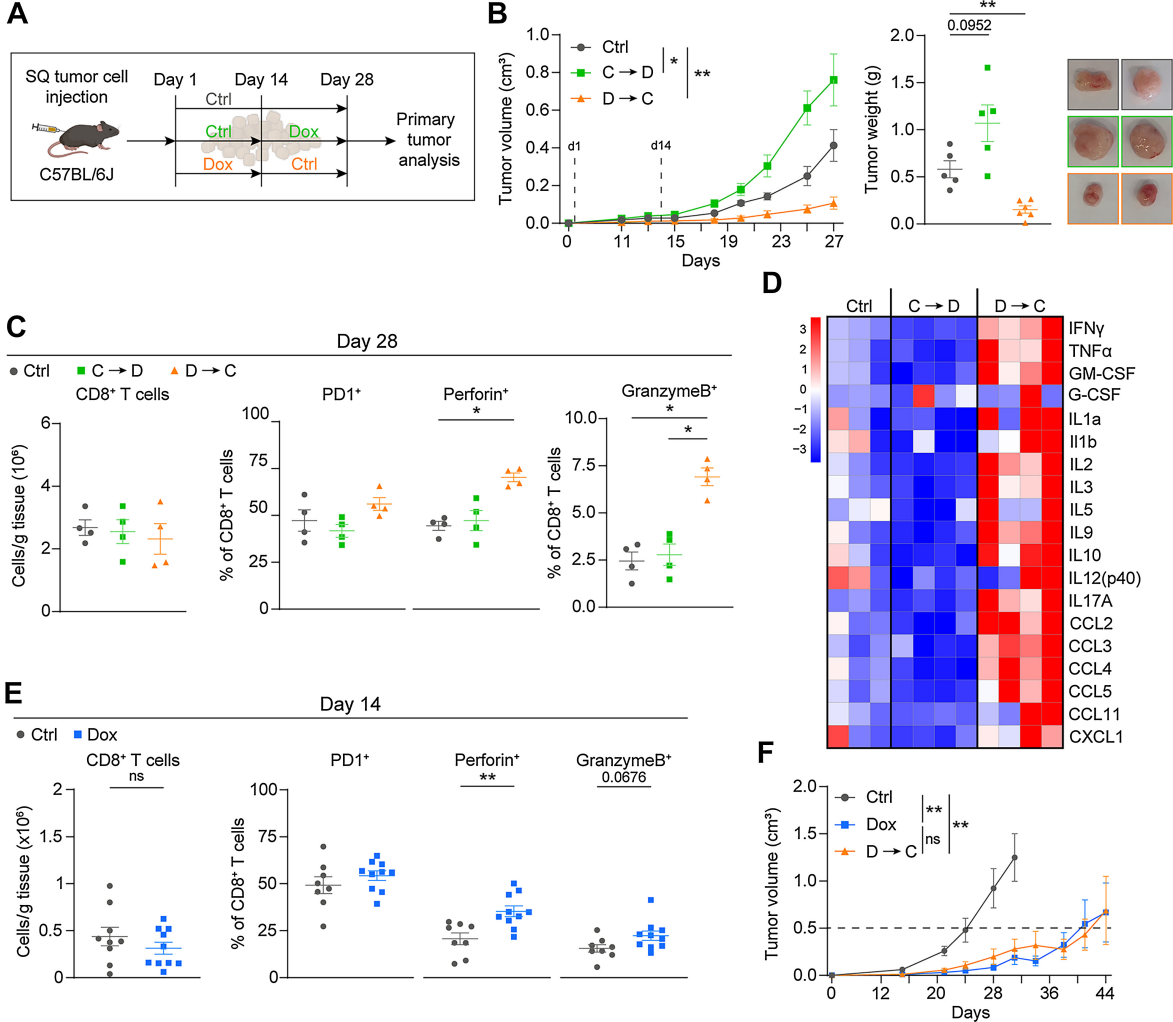
Early c-Myb upregulation is crucial for control of tumor growth. **A,** Experimental setup. C57BL/6J mice were subcutaneously injected with MC38^T/O^ cells and fed Ctrl or Dox chow. For two groups, chow is switched at day 14 for the rest of the experiment, Dox→Ctrl (D→C) or Ctrl→Dox (C→D). **B–D,** Tumor growth curves, final tumor weight, representative pictures (**B**); flow cytometry analysis (**C**); and Bio-plex analysis (**D**) of indicated groups are shown. Heat maps are shown of representative samples. **E,** Flow cytometry analysis of subcutaneous MC38^T/O^ tumors at day 14. Flow cytometry analysis for CD8^+^ T cells (CD45^+^CD11b^−^CD3e^+^CD8^+^) are represented as number of cells per gram of tumor tissue (**C, E**). CD8^+^PD1^+^/perforin^+^/granzymeB^+^ populations are represented as percentage of total CD8^+^ T cells (**C, E**). **F,** Long-term tumor growth was assessed in C57BL/6J mice subcutaneously injected with MC38^T/O^ cells and fed Ctrl or Dox chow for the duration of the experiment; or Dox chow for the first 14 days and subsequently switched to Ctrl chow for the rest of the experiment (D → C). *n* = 5–6 (**B**), *n* = 4 (**C**), *n* = 3–4 (**D**), *n* = 8–10 (**E**), *n* = 7–8 (**F**) mice per group. Data are presented as mean values ± SEM. Two-way ANOVA was used for comparing tumor growth curves (**B**, **F**). One-way ANOVA with Kruskal–Wallis posttest was used (**C–E**) for statistical analysis. *, *P* < 0.05; **, *P* < 0.01.

To better understand the influence of c-Myb upregulation on different stages of MC38 tumorigenesis, we performed experiments with Dox treatment either limited to the initial 2 weeks of tumor growth (Dox→Ctrl) or limited to weeks 3 and 4 of the experiment (Ctrl→Dox; Fig. 4A). In parallel, one group of mice received Ctrl chow for the entire duration of the experiment (Ctrl). We observed that tumors in the Ctrl→Dox group showed enhanced tumor growth compared to the Ctrl group, whereas tumors in the Dox→Ctrl group were significantly smaller (Fig. 4B). The quantification of c-Myb expression in tumors by IF showed that c-Myb was upregulated in the Ctrl→Dox group compared with the Ctrl group and was significantly downregulated in the Dox→Ctrl group compared with the Ctrl→Dox group (Supplementary Fig. S4A).

To understand the differences between the Dox→Ctrl and the Ctrl→Dox groups, we analyzed intratumoral immune cells and cytokines. Upregulation of c-Myb after initial tumor growth (Ctrl→Dox), did not result in changes in these parameters (Fig. 4C and D; Supplementary Figs. S4B and S4C). However, Dox→Ctrl tumors showed a pro-inflammatory and cytotoxic immune signature, similar to mice receiving Dox for the entire duration of the experiment (Fig. 3B and C). This signature was associated with enhanced effector molecules of CD8^+^ T cells and elevated levels of many cytokines in the TME, even when c-Myb was not upregulated anymore, resulting in efficient control of tumor growth (Fig. 4C and D). In addition, MHC-II expression on TAMs in the Dox→Ctrl group remained significantly higher than in the Ctrl group, even 2 weeks after removal of Dox-containing chow (Supplementary Fig. S4B). To evaluate whether the adaptive immune system was already altered at the time of Dox removal, we analyzed tumors at day 14 (Fig. 4E). Even though CD8^+^ T-cell infiltration was low at this time point, an increase in cell activation upon Dox treatment was already observed, as determined by the presence of intratumoral CD8^+^ T cells expressing perforin or granzyme-B. In addition, we observed increased c-Myb expression after day 14 (Supplementary Fig. S4D).

To investigate the lasting effect of c-Myb–induced control in MC38^T/O^ tumors, we subcutaneously injected C57BL/6J mice with the tumor cells and followed tumor growth over an extended time (Fig. 4F; Supplementary Fig. S4D). Mice were euthanized when humane endpoints were met. Both Dox→Ctrl and Dox groups showed significantly delayed tumor growth when compared with the Ctrl group. Ctrl tumors reached 0.5 cm^3^ on average at day 25, whereas tumors of animals receiving Dox→Ctrl or Dox reached this size at day 40 and 42, respectively (Fig. 4F). c-Myb expression was detected both in continuously Dox-treated and Dox→Ctrl mice (Supplementary Fig. S4F). In addition, three of eight (37.5%) and four of seven tumors (57.1%) in the Dox→Ctrl and Dox groups, respectively, were under 0.5 cm^3^ at the endpoint of the experiment (Supplementary Fig. S4D). These data demonstrate that c-Myb upregulation during early tumorigenesis is sufficient to shape the TME and to promote tumor control.

### c-Myb upregulation in tumor cells induces changes in immunomodulation

**Figure 5.**
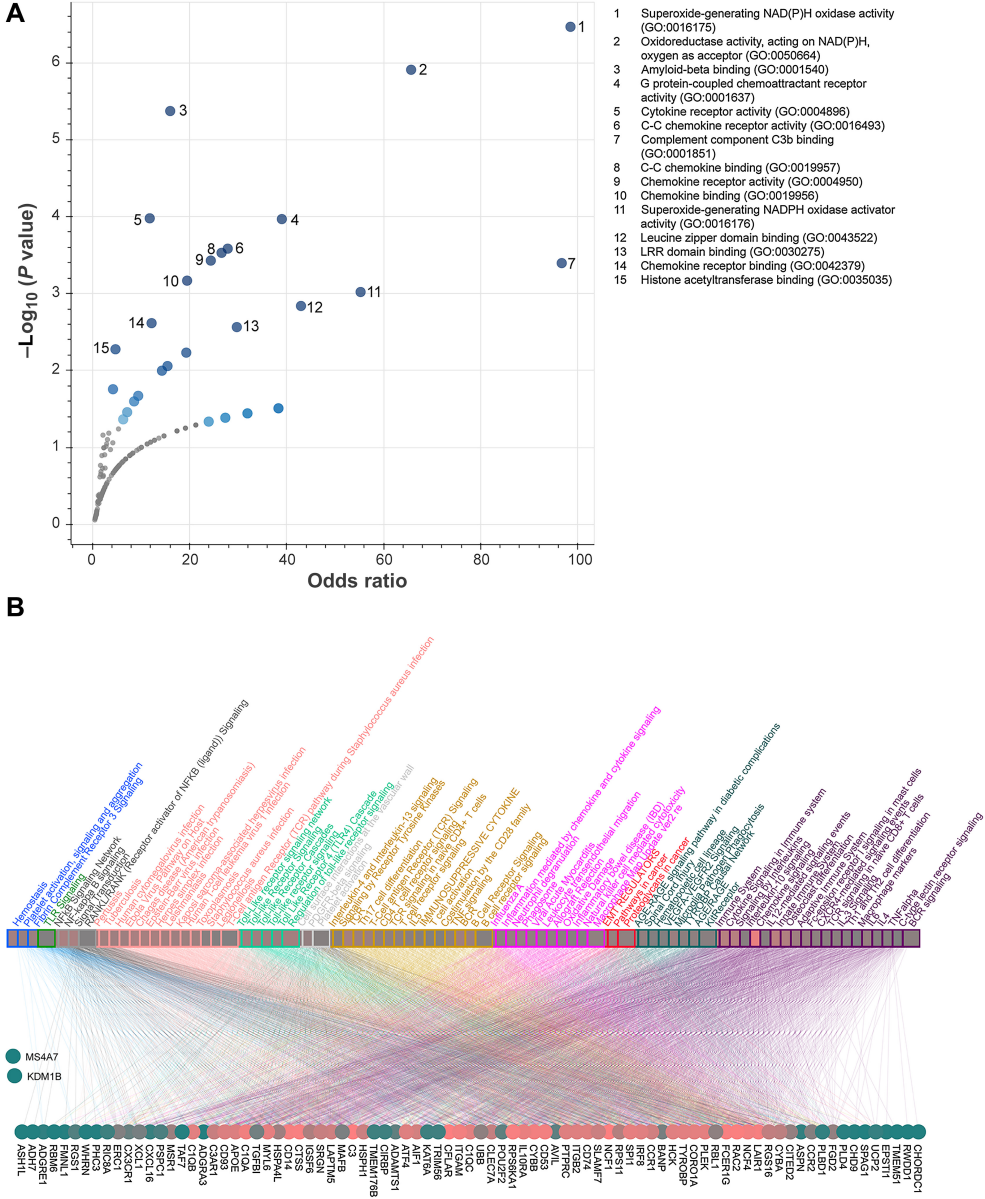
c-Myb upregulation in MC38 tumor cells modulates genes involved in immune responses. **A,** Gene Ontology enrichment analysis of genes significantly dysregulated between tumor cells from mice on Dox- and Ctrl-chow using Enrichr tool (https://maayanlab.cloud/Enrichr). Sorted CD45^−^GFP^+^ tumor cells from subcutaneous MC38^T/O^ tumors in C57BL/6J mice, after 21 days were analyzed by RNA-seq (*n* = 4). **B,** Using the list of 87 dysregulated genes as input to pathDIP (https://ophid.utoronto.ca/pathDIP), we identified significantly enriched pathways (Supplementary Table S3). Here we highlight pathway-gene associations for the top 10% significantly enriched pathways (i.e., more than 35 of the input DEGs present; listed in Supplementary Table S4). Similar pathways are grouped and color-coded (both pathway name and edge color that connects to corresponding DEGs). Number of gene–pathway associations (i.e., node degree) is reflected by node color shade from green (low degree; only a few pathways or a few genes) through red (high degree; many pathways or many genes). The node degree for genes ranges from 0 (MS4A7, KDM1B) to 90 (CD74, PTPRC, ITGB2), and for pathways from 35 (hematopoietic cell lineage, VEGFA-VEGFR2 Signaling, T-cell activation, costimulation by the CD28 family, TLR signaling, macrophage markers, T-cell antigen receptor pathway during *Staphylococcus aureus* infection, C-type lectin receptor signaling, RANKL/RANK Signaling) to 71 (chemokine signaling).

To understand how c-Myb-regulated genes in tumor cells affected the TME resulting in reduced tumor growth, we performed RNA-seq in sorted GFP^+^ tumor cells from subcutaneous MC38^T/O^ tumors from Ctrl and Dox-treated mice (Supplementary Fig. S5A). Significantly altered genes were analyzed by gene ontology enrichment analysis (Fig. 5A; Supplementary Table S4). In c-Myb–expressing tumors, we observed dysregulation in genes associated with cytokine activity, chemokine receptors, and G-protein chemoattractant receptor activity, as well as immune cell–recruitment and –activation. Pathway analysis of genes dysregulated in c-Myb–expressing tumors using pathDIP revealed an overall strong association with the immune system and cytokine signaling pathways, where over 50 of 87 genes were associated with these pathways (Fig. 5B; Supplementary Tables S3 and S4). Further analysis of the top 10 dysregulated genes revealed an overall downregulation of genes in complement pathway activation and monocytic-cell recruitment and maturation (Supplementary Fig. S5B), showing the repressor function of c-Myb (21).

**Figure 6.**
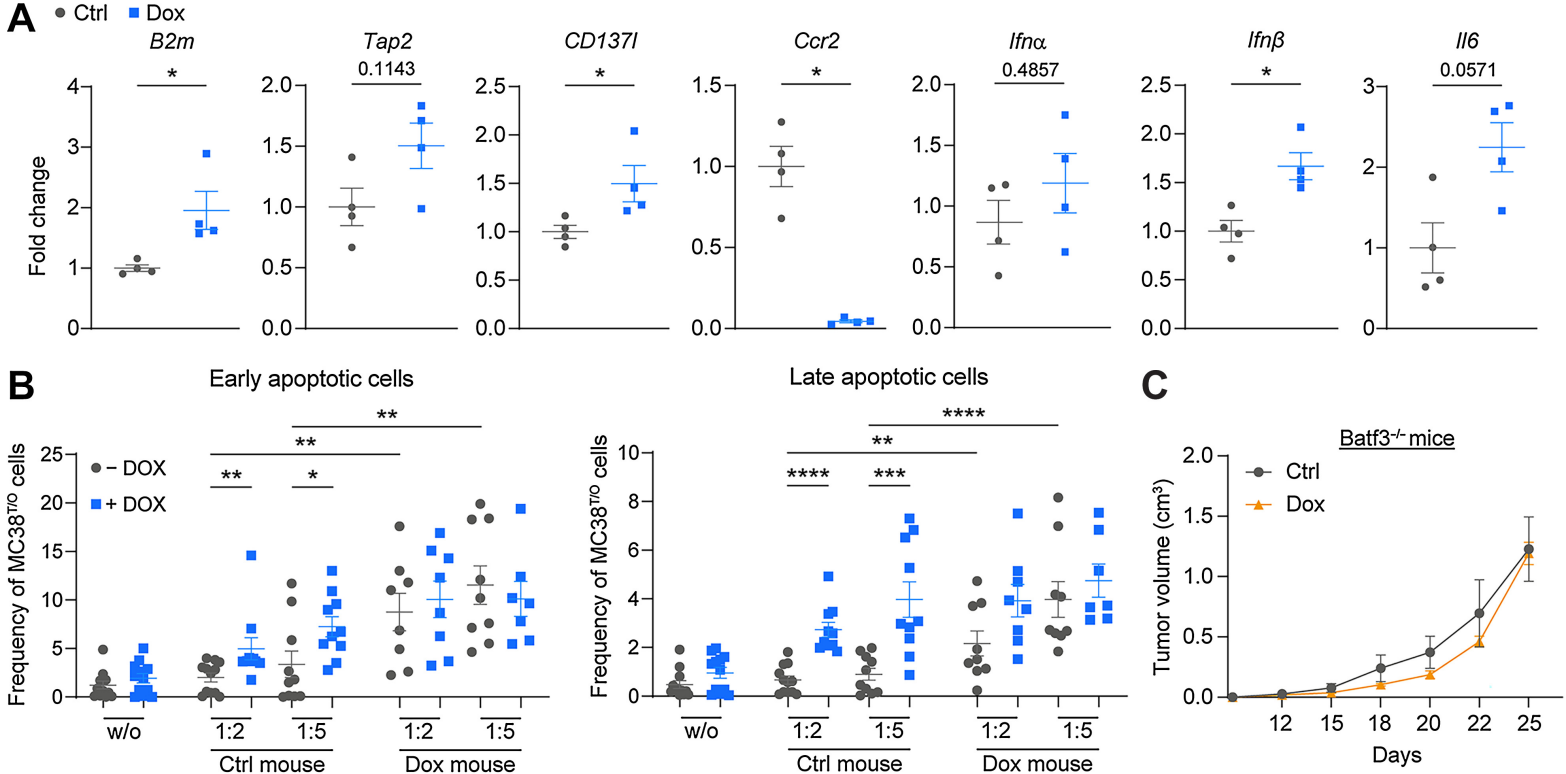
c-Myb upregulation regulates intrinsic changes in MC38 tumor cells that control tumor growth by CD8^+^ T cells. **A,** qRT-PCR data for *B2m*, antigen peptide transporter 2 (*Tap2*), CD137 ligand (*Cd137L*), *Ccr2*, *Ifnβ*, and *Il6* is normalized to *Gapdh* expression and relative to the Ctrl group (*n* = 4 mice per group). **B,** Killing assay of cultured MC38^T/O^ cells; untreated (− Dox, grey) or treated with Dox for 24 hours (+ Dox, blue) prior to coculture. Twenty-four hours after the addition of enriched CD8^+^ T cells (tumor cell: CD8^+^ T cell ratio 1:2 / 1:5) from a spleen of a subcutaneous MC38^T/O^ tumor-bearing C57BL/6J mouse (Ctrl- or Dox-mouse), early (AnnexinV^+^7-AAD^−^) and late (AnnexinV^+^7-AAD^+^) apoptotic tumor cells were assessed by flow cytometry. Data are presented as percentage of total MC38^T/O^ cells. w/o: tumor cells cultured without CD8^+^ T cells. All data are presented from three independent experiments **C,***Batf3*^−/−^ mice were subcutaneously injected with MC38^T/O^ cells and were fed Ctrl or Dox containing chow for the duration of the experiment. *n* = 5 mice per group. Tumor growth is shown. Data are presented as mean values ± SEM. Mann–Whitney test was used for statistical analysis (**A**). One-way ANOVA with Kruskal–Wallis posttest was used for statistical analysis of apoptosis (**B**). Two-way ANOVA was used for comparing tumor growth curves (**C**). *, *P* < 0.05, **, *P* < 0.01, ***, *P* < 0.001, ****, *P* < 0.0001.

Next, we analyzed tumor cells from c-Myb–expressing and control tumors for genes involved in immune activation and antigen presentation. Analysis of sorted MC38^T/O^ tumor cells from Dox-treated mice showed elevated levels of *Il6*, β2-microglobulin (*B2m*), which associates with the MHC-I complex, and CD137-ligand (*Cd137L*), a ligand of the costimulatory receptor CD137, which is expressed on effector T lymphocytes (Fig. 6A). In addition, *Tap2*, another gene involved in antigen processing and presentation also showed a tendency to increased expression *in vivo* (Fig. 6A). In addition, we observed increased expression of the type I IFN *Ifnβ* (Fig. 6A); increased *Ifnα* was not statistically significant. Similarly, upregulation of *Il6*, *B2m*, and *Cd137L* was observed in MC38^T/O^ cells treated with Dox *in vitro* (Supplementary Fig. S6A). Finally, significant downregulation of *Ccr2* in c-Myb induced tumor cells was detected (Fig. 6A). In contrast, we observed no major changes in immune-related genes in either LLC1.1^T/O^ or B16-BL6^T/O^ cells treated with Dox *in vitro*, or in the tumors from Dox-treated mice injected with LLC1.1^T/O^ or B16-BL6^T/O^ tumor cells (Supplementary Fig. S6B). Similarly, no significant changes in immune-related genes were observed in CT26^T/O^ colorectal cells and Dox-induced c-Myb expression in mice injected with CT26^T/O^ cells did not affect tumor growth (Supplementary Fig. S6C), which is in agreement with previous study by Millen and colleagues (12).

### c-Myb upregulation in MC38 tumor cells enhances CD8^+^ T-cell cytotoxicity

We tested the hypothesis that c-Myb regulated genes involved in immune modulation, including antigen presentation, in tumor cells could directly enhance T cell–mediated cytotoxicity. We performed *in vitro* killing assays using (un)treated MC38^T/O^ cells, which were incubated with splenic CD8^+^ T cells isolated from Ctrl or Dox-treated MC38^T/O^ tumor-bearing mice (Supplementary Fig. S6D). Analysis of early and late apoptotic tumor cells showed an increase in CD8^+^ T cell–mediated cytotoxicity in MC38^T/O^ cells treated with Dox compared with untreated tumor cells (Fig. 6B). In addition, CD8^+^ T cells from Dox-treated mice had a higher cytotoxic capacity than CD8^+^ T cells from Ctrl-treated mice, independently of the tumor cell treatment *in vitro*. These results suggest that c-Myb–expressing MC38^T/O^ tumor cells can directly enhance CD8^+^ T-cell cytotoxicity.

Next, we performed the same experiment with the B16-BL6^T/O^ and LLC^T/O^ models (Supplementary Fig. S6E). It is known that both B16-BL6 and LLC tumors are poorly immunogenic (26, 27). Accordingly, we observed no significant induction of tumor cell apoptosis after coculture of (un)treated LLC^T/O^ tumor cells with CD8^+^ T cells. No significant differences in tumor cell death were observed between treated and untreated B16-BL6^T/O^ melanoma cells, although CD8^+^ T cells from Dox-treated mice were more cytotoxic compared with CD8^+^ T cells from Ctrl-treated mice.

Finally, we tested the hypothesis that cross-presentation of tumor antigen is required for efficient control of tumor growth. The intratumoral presence of DCs expressing the transcription factor Batf3 is required for cross-priming and recruitment of antitumor effector T cells (28, 29). We injected MC38^T/O^ cells subcutaneously into *Batf3*^−/−^ mice and followed tumor growth in animals either fed on Ctrl or Dox chow (Fig. 6C; Supplementary Fig. S6F). Induced c-Myb expression did not affect tumor growth in *Batf3*^−/−^ mice, indicating that tumor-derived antigens cross-presented by DCs were required for effective T-cell control of tumor progression.

### c-Myb is a possible prognostic biomarker for specific molecular subtypes of patients with colorectal cancer

Although c-Myb is overexpressed in over 80% of patients with colorectal cancer (3), its consequence for tumorigenesis and metastasis remains unclear. Therefore, we performed survival analysis of 562 patients with colorectal cancer in the dataset GSE39582 (20). Although overall *MYB* transcript levels were not significantly associated with patient survival, patients with specific molecular subtypes of colorectal cancer enriched in stem cell/EMT features (C4 and C6) had significantly longer survival when high *MYB* expression was detected (Supplementary Fig. S7). This analysis points to a specific context–dependent function of MYB in modulating cancer progression.

## Discussion

The transcriptional regulator c-Myb is involved in a wide range of developmental and homeostatic processes throughout the body. Mutations or dysregulation of c-Myb can result in abnormal expression levels and contribute to tumorigenesis (3). In different subsets of leukemia, c-Myb has been described as an essential component for maintained proliferation and aberrant gene expression programs. Consequently, targeting c-Myb in leukemia is in focus as an anticancer therapy (30).

The role of dysregulated c-Myb expression in solid tumors remains poorly understood. Even though c-Myb expression is crucial for the development of colorectal cancer (7), c-Myb expression correlates with a better prognosis for these patients (8, 10). We set out to study the role of c-Myb in colorectal cancer, and evaluate it in parallel in lung cancer and melanoma mouse models. Both, MC38 colorectal cancer and melanoma models showed a significant decrease in tumor growth upon induction of c-Myb expression. Of note, c-Myb upregulation in a breast cancer model has been shown to delay primary tumor growth (31, 32). The upregulation of c-Myb in our inducible model is approximately 1.5- to 2-fold *in vitro*, depending on the cell line, which corresponds to dysregulated expression observed in patients with acute lymphoblastic leukemia or colorectal cancer (33, 34).

Further analysis of the colorectal cancer model revealed that c-Myb upregulation during tumorigenesis did not affect tumor cell proliferation, apoptosis, or tumor angiogenesis. However, we observed changes in the TME, specifically the number and activation status of immune cells that correlated with changes in cytokine expression. First, we observed a decrease in TAMs in tumors with c-Myb induction. A negative correlation between c-Myb and infiltrated TAMs has been shown before in human breast cancer (35). Macrophages in the TME have a highly dynamic nature and can either polarize into an immunosuppressive population or stimulate antitumor responses (36). Upon c-Myb upregulation, a shift in TAM phenotype towards a pro-inflammatory state with enhanced MHC-II expression was observed. The pro-inflammatory phenotype of macrophages has been shown to enhance T-cell activity (36, 37). Accordingly, we observed increased numbers of CD8^+^ T cells expressing granzyme-B and perforin in c-Myb–induced tumors. Second, we found an increased number of eosinophils in the TME. Eosinophils are innate immune cells that can exert both pro- and antitumorigenic functions (38). IL5 and CCL11 are crucial cytokines for their maturation and migration and both were found significantly upregulated in the TME of c-Myb–expressing tumors. Although eosinophils in colon cancer mouse models have shown antitumor immunity, higher numbers of eosinophils in the tumor correlated with increased CD8^+^ T-cell numbers and a better prognosis for patients with cancer (39). Third, we detected increased numbers of CD103^+^ DCs in c-Myb–expressing tumors. Taken together, the immune cell composition in c-Myb–upregulated tumors shifted to a more antitumorigenic state, showing an increased antigen-presenting phenotype and more perforin^+^/granzymeB^+^/CD8^+^ T cells.

Tumors cells try to evade the immune system by creating an immunosuppressive microenvironment through the production of cytokines and chemokines (40). We observed significantly increased protein levels of several cytokines in tumors upon c-Myb expression, including IFNγ, TNFα, CXCL16, CCL2, etc. Previously, it has been shown that type I and type II IFNs can activate antitumorigenic macrophages and result in the activation of CD8^+^ T cells and a subsequent reduction of tumor growth in a breast cancer model (41). Type I and II IFNs are known to induce the production of cytokines, including CXCL9 and CXCL10, which play a role in immune cell migration, differentiation, and activation and ultimately in tumor suppression (42). We did not observe changes in T-cell infiltration in our MC38 tumor model, which is a tumor with higher immune cell infiltration and has high basal levels of cytokines, including CXCL10. However, we did find an increase in T-cell numbers in our B16-BL6 melanoma model upon c-Myb upregulation, which is considered a poorly immune cell infiltrated tumor model. In the MC38 colon cancer model, we detected higher levels of CXCL16 upon c-Myb expression. Enhanced CXCL16 expression in colon cancer has been shown to correlate with increased T-cell infiltration and better prognosis in patients with cancer (25).

Another mechanism of immune evasion is the prevention of immune recognition and subsequent elimination, which can be orchestrated by tumor cells through downregulation of the antigen processing and presentation machinery (43). Tumor cells with a high mutational burden often present more tumor-specific neoantigens, which enables the immune system to recognize these tumors cells (44). Of the three cell lines used in this study, MC38 tumor cells have the highest mutational burden (45) with several MC38-specific neoantigens that can be presented by MHC-I on MC38 tumor cells and recognized by antigen-specific CD8^+^ T cells (46). Upregulation of c-Myb in tumors resulted in enhanced expression of B2M, part of the MHC-I complex, suggesting that antigen presentation in these tumor cells is likely increased. Recently, patients with melanoma who did not respond to anti-PD1 therapy were shown to carry mutations in pathways involved in IFN-receptor signaling and in antigen presentation, such as B2M (47). Furthermore, TAP2, which is involved in the antigen-processing machinery, IL6 and IFNβ were significantly upregulated in our c-Myb–expressing MC38 tumors. Type I IFNs are known to suppress tumor growth through the activation of cytotoxic T cells (48). In addition, significant downregulation of CCR2 expression on c-Myb upregulated tumors cells was observed. CCR2 is a chemokine receptor expressed by a wide range of immune cells but also by different types of tumor cells. Recently, it was shown that the deletion of CCR2 in breast cancer cells leads to a reduction of tumor growth through changes in the TME, including increased infiltration and activation of CD8^+^ T cells and cross-presenting CD103^+^ DCs, and the upregulation of MHCI expression (49). Our killing assays using MC38 colon cancer cells show that tumor cell intrinsic changes upon c-Myb upregulation can directly increase CD8^+^ T cell–mediated cytotoxicity. An attenuation of tumor growth was observed in the B16-BL6 tumors upon c-Myb induction *in vivo*, and there was increased tumor cell sensitivity to CD8^+^ T cell–mediated killing *in vitro*, albeit not statistically significant. Because c-Myb is known to have cell type–specific target genes, we reason that different target genes are activated and/or inhibited in the B16-BL6 tumor cells, which requires further analysis. The observed changes in T-cell numbers in the B16-BL6 model, further support this hypothesis.

In colorectal cancer, increased expression of c-Myb has been detected in over 80% of patients (3), whereas changes in c-Myb expression have been observed also in lung cancer and melanoma cells (50, 51). We tested the effect of increased c-Myb expression in two colon carcinoma cells MC38 and CT26, LLC1.1 lung carcinoma cells, and B16 melanoma cells, showing effective tumor growth control only in MC38 and B16-BL6 models. In the CT26 colon cancer model, we observed no effect, which is in agreement with previous results, where the overexpression of c-Myb resulted in immune suppression and no effect on tumor growth (12). Thus, the effectiveness of c-Myb–induced control of MC38 tumor growth might be due to the mesenchymal phenotype and microsatellite instability features that are inherently immunosuppressive (52). Furthermore, the clinical evidence shows a better overall survival for patients with cancer of a specific group with high *MYB* expression and an enhanced mesenchymal phenotype (20), which correlates well with the findings in the MC38 model. In addition, the known poor immunogenicity of LLC1.1, CT26, and B16-BL6 tumor cells could explain the nonresponsiveness to increased c-Myb expression in these models. Overall, our data show that c-Myb upregulation in MC38 changes gene expression that increases immunogenicity and thereby contributes to the control of tumor growth. We provide evidence that c-Myb in combination with an immunogenic cell-specific signature, can function as a tumor suppressor.

## Supplementary Material

Supplementary Data Supplementary Figures and Tables
