# Supplementary material for: Tumor Cell–Intrinsic c-Myb Upregulation Stimulates Antitumor Immunity in a Murine Colorectal Cancer Model
Source: Cancer Immunol Res. 2023 Jul 21;11(10):1432–44. doi: 10.1158/2326-6066.CIR-22-0912 (PMC10548106; doi:10.1158/2326-6066.CIR-22-0912)
Supplement: Supplementary Data — Supplementary Figures and Tables [file cir-22-0912_supplementary_data_suppst1-st4_sf1-sf7.pdf]

Supplementary Data

van Gogh *et al.*

Tumor cell-intrinsic c-Myb upregulation stimulates anti-tumor immunity in a murine colorectal cancer model

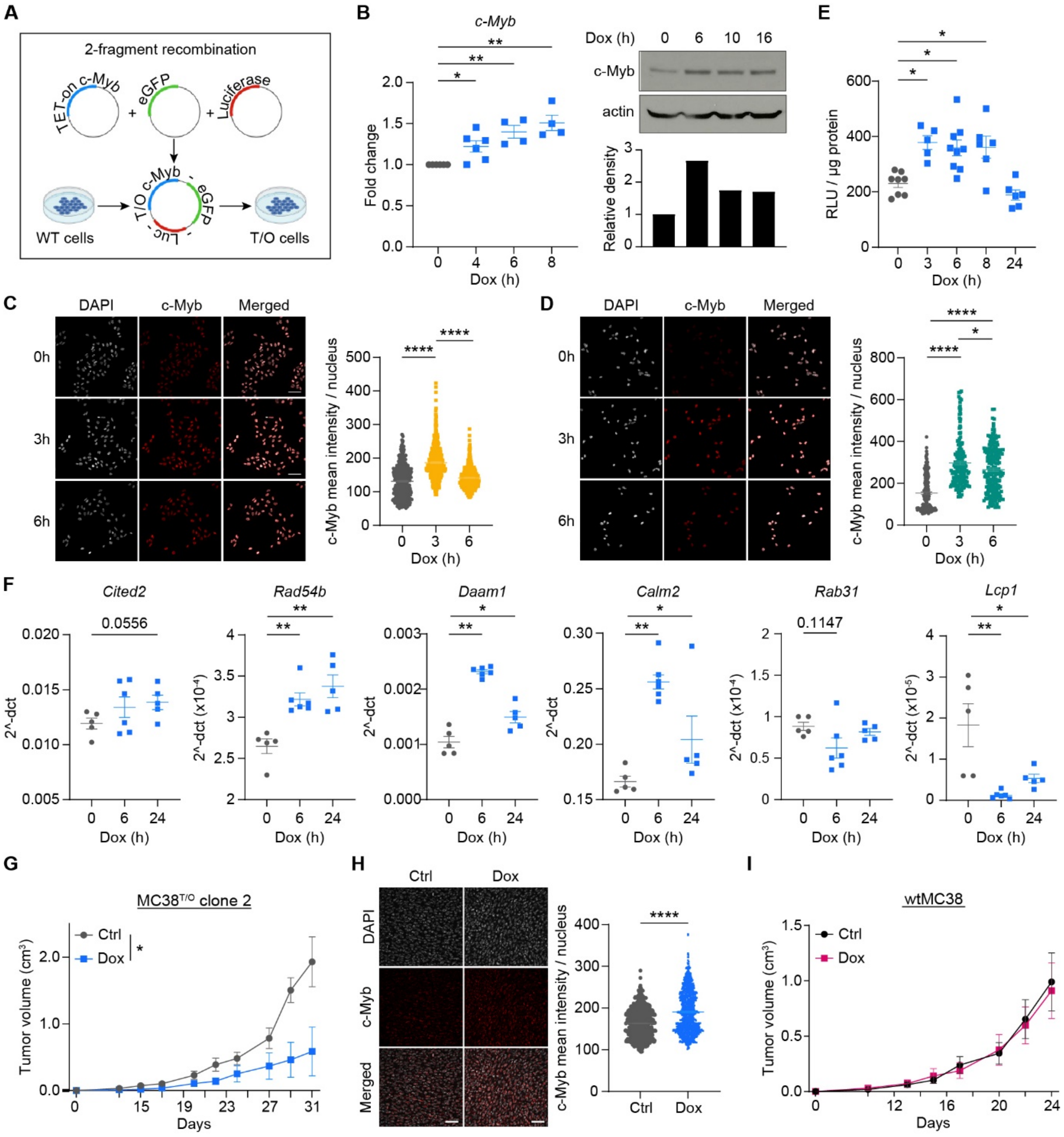

Supplementary Figure S1. Doxycycline inducible cell line preparation and characterization. A, Two-fragment recombination was used to create one plasmid which contains constitutively active eGFP (SV40 promoter) and Firefly Luciferase (PGK promoter) and doxycycline (Dox) inducible c-Myb. Wild-type MC38 colon cancer, LLC1.1 Lewis Lung Carcinoma and B16-BL6 melanoma cells were transduced with this plasmid to create Dox inducible cell lines (TET-on); MC38$^{T/O}$, LLC$^{T/O}$ and B16-BL6$^{T/O}$, respectively. **B,** left panel: RT-qPCR data of c-Myb of in vitro Dox treated MC38$^{T/O}$ cells. Data is normalized to GAPDH expression and relative to the untreated cells (0h). Right panel: Representative Western Blot for c-Myb of in vitro Dox treated MC38$^{T/O}$ cells for the indicated time points. Quantification data is normalized against actin loading control and represented as relative density. **C-D,** Immunofluorescence staining for c-Myb (red) in B16-BL6$^{T/O}$ (**C**) and LLC$^{T/O}$ (**D**) cells, treated for the indicated time points with 1µg/ml Dox in vitro. c-Myb staining is quantified as mean intensity per nucleus (white). Data are presented from two independent experiments. Each data point represents a nucleus. **E,** Luciferase assay of wtMC38 cells transiently transfected with a Dox inducible c-Myb plasmid

(TET-on c-Myb – eGFP – Neomycin) and a c-Myb reporter plasmid (p6MBS/luc; containing six c-Myb binding sites), treated with Dox (1µg/ml) for the indicated time points. Luciferase activity is represented as relative light units (RLU) per µg protein. Data are presented from three independent experiments. **F,** RT-qPCR data of indicated known c-Myb target genes of in vitro Dox treated MC38T/O cells. Data is normalized to GAPDH expression. Cited2, Rad54b, Daam1 and Calm2 are known c-Myb induced and Rab31 and Lcp1 are c-Myb reduced target genes. **G,** Tumor growth curves of MC38T/O clone 2 subcutaneous tumors in C57BL/6J mice, fed Ctrl or Dox containing chow. **H,** Immunofluorescence staining for c-Myb (red) of formalin-fixed paraffin embedded MC38T/O subcutaneous tumor sections of mice fed Ctrl or Dox containing chow for 28 days. c-Myb staining is quantified as mean intensity per nucleus (white). Each dot represents one nucleus. **I,** Tumor growth curves of wild-type (wt) MC38 subcutaneous tumors in C57BL/6J mice fed Ctrl or Dox chow. n=4 (**G-H**); n=7-9 mice per group from two independent experiments (**I**). *p<0.05, ****p<0.0001. Scale bars: 50µm.

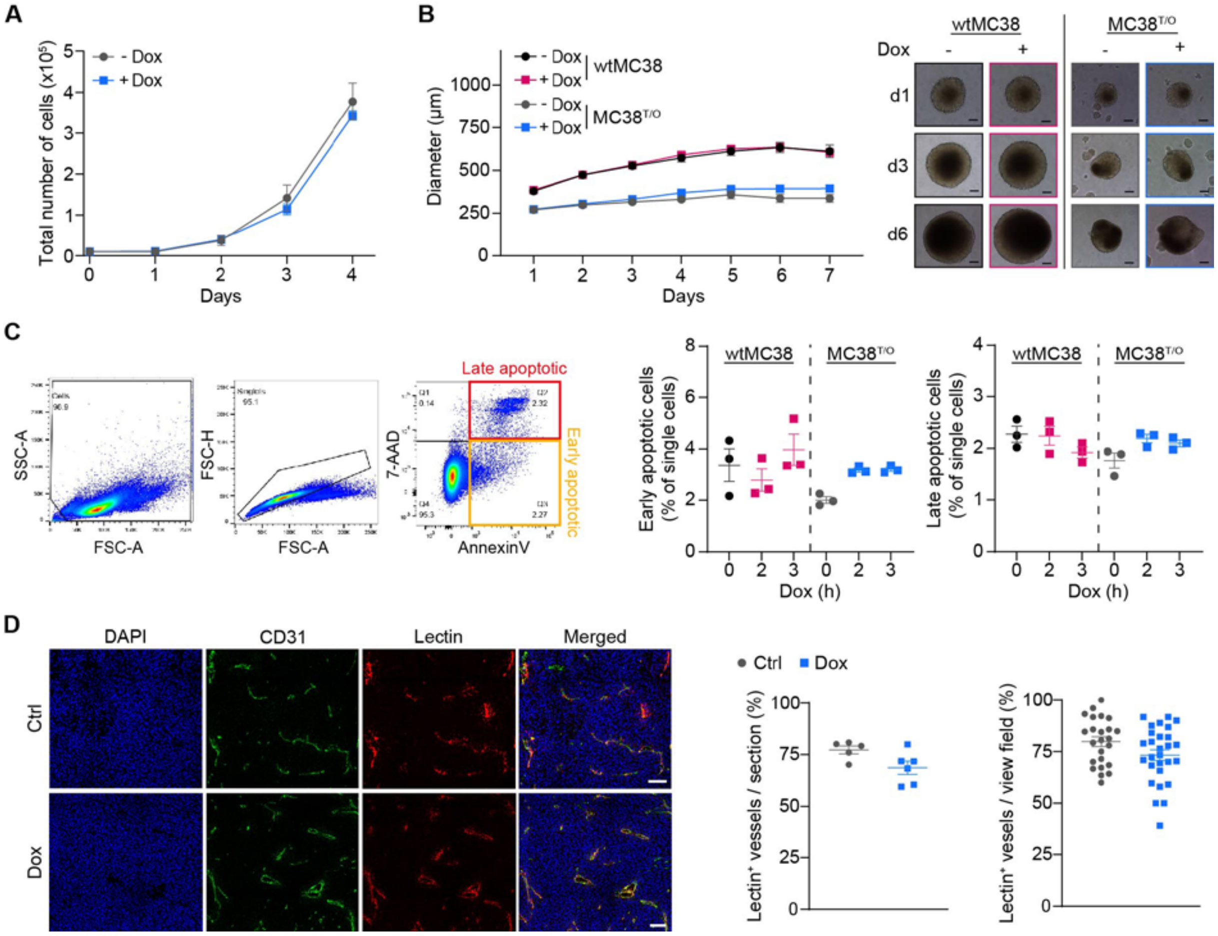

Supplementary Figure S2. c-Myb upregulation does not alter MC38 intrinsic properties or angiogenesis Part II. A-B, *In vitro* cell proliferation of MC38$^{T/O}$ cells in 2D (**A**) and 3D (**B**) cultures, treated with doxycycline (Dox) for the indicated time points. Cell proliferation was assessed by manual counting using Trypan Blue (2D culture) or measuring the diameter of the spheroids (3D culture). Representative pictures of 3D spheroids are shown (**B** right panel). **C,** Apoptotic wtMC38 and MC38$^{T/O}$ cell analysis upon Dox treatment as assessed by Flow Cytometry (left panel). Early (AnnexinV^{+} 7-AAD^{-}) and late (AnnexinV^{+} 7-AAD^{+}) apoptotic cells are presented as percentage of total single cells (right panel). **D,** Vessel perfusion in MC38$^{T/O}$ tumors from Ctrl or Dox treated mice was assessed by intravenous injection of fluorescently labeled lectin 10 minutes before tumor resection. Tumor sections were stained for CD31 (green) and lectin positive vessels (red) were quantified in the whole tumor section (middle) and per view field (right). Each condition is measured in triplicates (**A-C**) or n=5-6 mice per group from two independent experiments (**D**). Scale bars: 100µm.

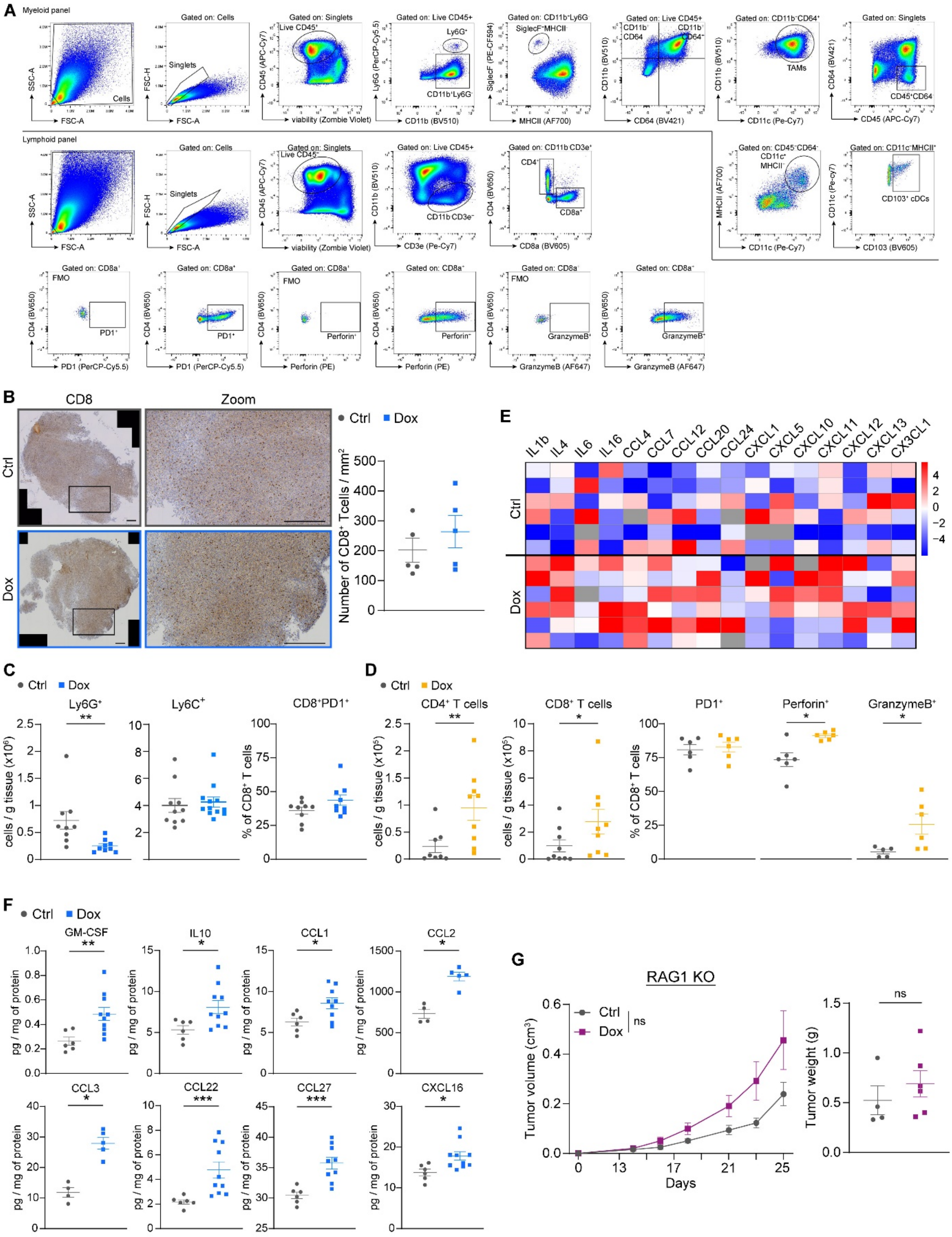

Supplementary Figure S3. c-Myb upregulation results in changes in the tumor microenvironment Part II. A, Flow cytometry gating strategy for immune cells. **B,** Representative images of CD8 stained formalin-fixed paraffin embedded MC38$^{T/O}$ subcutaneous tumor sections of control (Ctrl) or doxycycline (Dox) treated mice (left). Quantification of CD8^{+} T cells per tumor area (right). **C,** Flow cytometry analysis of MC38$^{T/O}$ SQ tumors of Ctrl or Dox treated mice for Ly6G^{+} cells (CD45^{+}CD11b^{+}CD64^{-}CD11c^{-}Ly6G^{+}), Ly6C^{+} cells (CD45^{+}CD11b^{+}CD64^{-}CD11c^{-}Ly6C^{+}) represented as the

number of cells per gram of tumor tissue, and $CD8^+PD1^+$ T cells ($CD45^+CD11b^-CD3e^+CD8^+$ $PD1^+$); represented as percentage of total $CD8^+$ T cells. **D,** Flow cytometry analysis of B16-BL6$^{T/O}$ SQ tumors from Ctrl or Dox treated mice for $CD4^+$/$CD8^+$ T cells ($CD45^+CD11b^-CD3e^+CD4^+$ or $CD8^+$ respectively). Data is represented as the number of cells per gram of tumor tissue. $CD8^+PD1^+$/ $Perforin^+$/ $GranzymeB^+$ populations are represented as percentage of total $CD8^+$ T cells. **E,** Heatmap of Bioplex analysis of MC38$^{T/O}$ SQ tumor homogenates of Ctrl and Dox treated mice. **F,** Representative graphs of Bio-plex data from Figure 3C. Values are represented as pg of cytokine per mg of total protein. **G,** Tumor growth and final tumor weight of RAG1 deficient (KO) mice subcutaneously injected with MC38$^{T/O}$ cells, fed Ctrl or Dox chow. n=5 (**B**), n=9-12 (**C**), n=5-9 (**D**), n=6 (**E**), n=4-10 (**F**) or n=4-6 (**G**) mice per group, from two independent experiments. *p<0.05, **p<0.01, ***p<0.001, ns = not significant. Scale bar: 500µm.

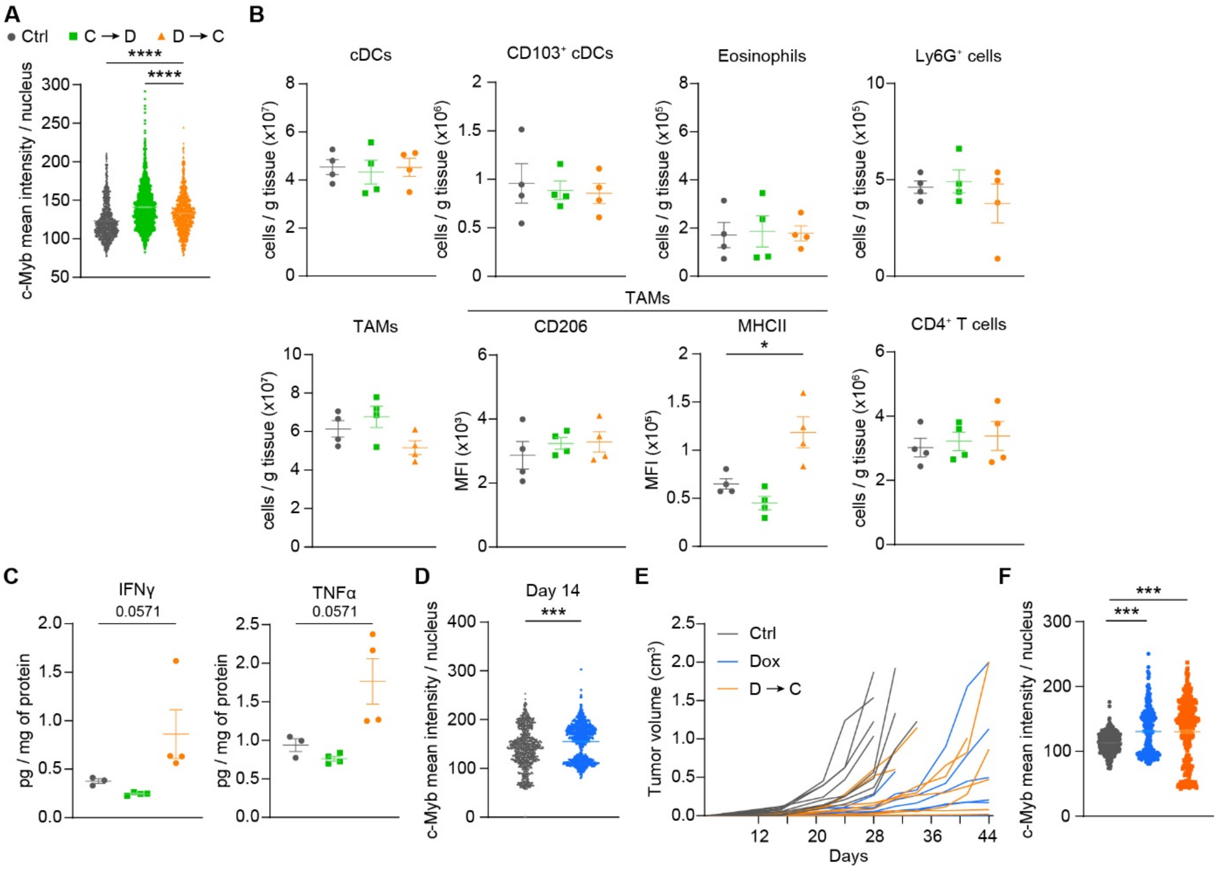

Supplementary Figure S4. Early c-Myb upregulation is crucial for control of tumor growth Part II. A, Quantification of immunofluorescence staining for c-Myb of formalin-fixed paraffin embedded MC38$^{T/O}$ subcutaneous tumor sections from C57BL/6J mice treated as indicated. Each dot represents one nucleus. **B,** Flow cytometry analysis for tumor-associated macrophages (TAMs; CD45$^+$CD11b$^+$CD64$^+$CD11c$^+$), eosinophils (CD45$^+$CD11b$^+$Ly6G$^-$MHCII$^-$SiglecF$^+$), Ly6G$^+$ cells (CD45$^+$CD11b$^+$CD64$^-$CD11c$^-$Ly6G$^+$) and CD4$^+$ T cells (CD45$^+$CD11b$^-$CD3e$^+$CD4$^+$) of MC38$^{T/O}$ subcutaneous tumors from C57BL/6J mice treated as indicated. Data is represented as number of cells per gram of tumor tissue. Median fluorescent intensity (MFI) is shown for CD206 and MHCII on TAMs. **C,** In relation to Bio-plex analysis in Fig. 4d: Graphs of absolute values are represented as pg of cytokine per mg of total protein. **D,** Tumors at day 14 (related to Figure 4f). c-Myb staining is quantified as mean intensity per nucleus (white). Each dot represents one nucleus. Grey = Ctrl (no Dox), Blue = Dox treatment for 14 days. **E,** Individual tumor growth curves of C57BL/6J mice subcutaneously injected with MC38$^{T/O}$ cells, provided with Ctrl or Dox chow for the entire duration of the experiment, or Dox chow for the first 14 days and subsequently switched to Ctrl chow for the remainder of the experiment (D → C) (related to Figure 4f). **F,** Tumors at day of termination as indicated in panel E. c-Myb staining is quantified as mean intensity per nucleus (white). Each dot represents one nucleus. Color coding the same as in panel E. n=3 (**A**), n=4 (**B**), n=3-4 (**C**) or n=7-8 (**D**) mice per group. *$p<0.05$, ****$p<0.0001$.

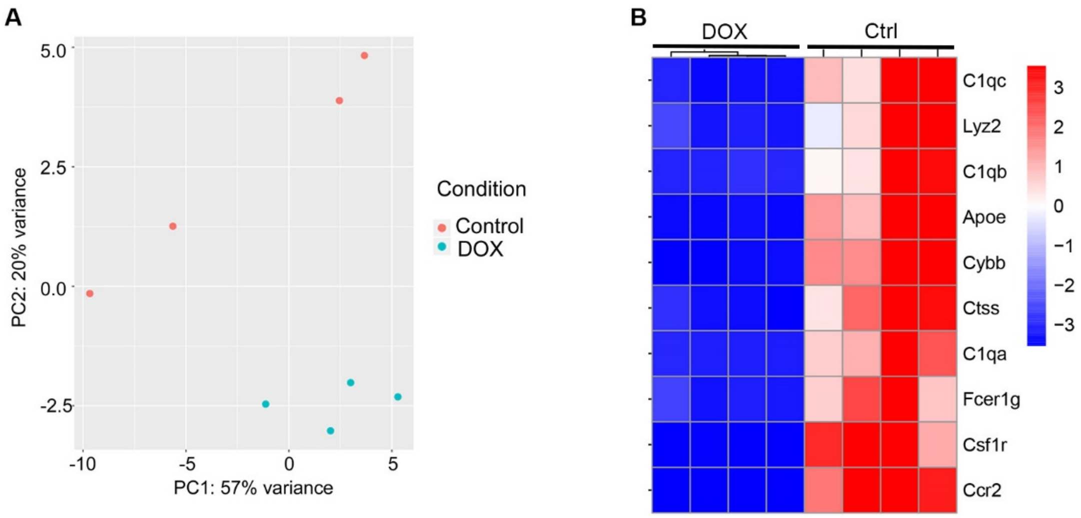

Supplementary Figure S5. c-Myb upregulation regulates intrinsic changes in MC38 tumor cells. The analysis of GSE232823 datasets. **A,** Principal component analysis of RNA sequencing data from tumor cells sorted from mice on Dox or control-chow for 21 days. **B,** Heatmap representation of the top 10 significantly differentially regulated genes between tumor cells from Dox-treated or Ctrl-treated mice.

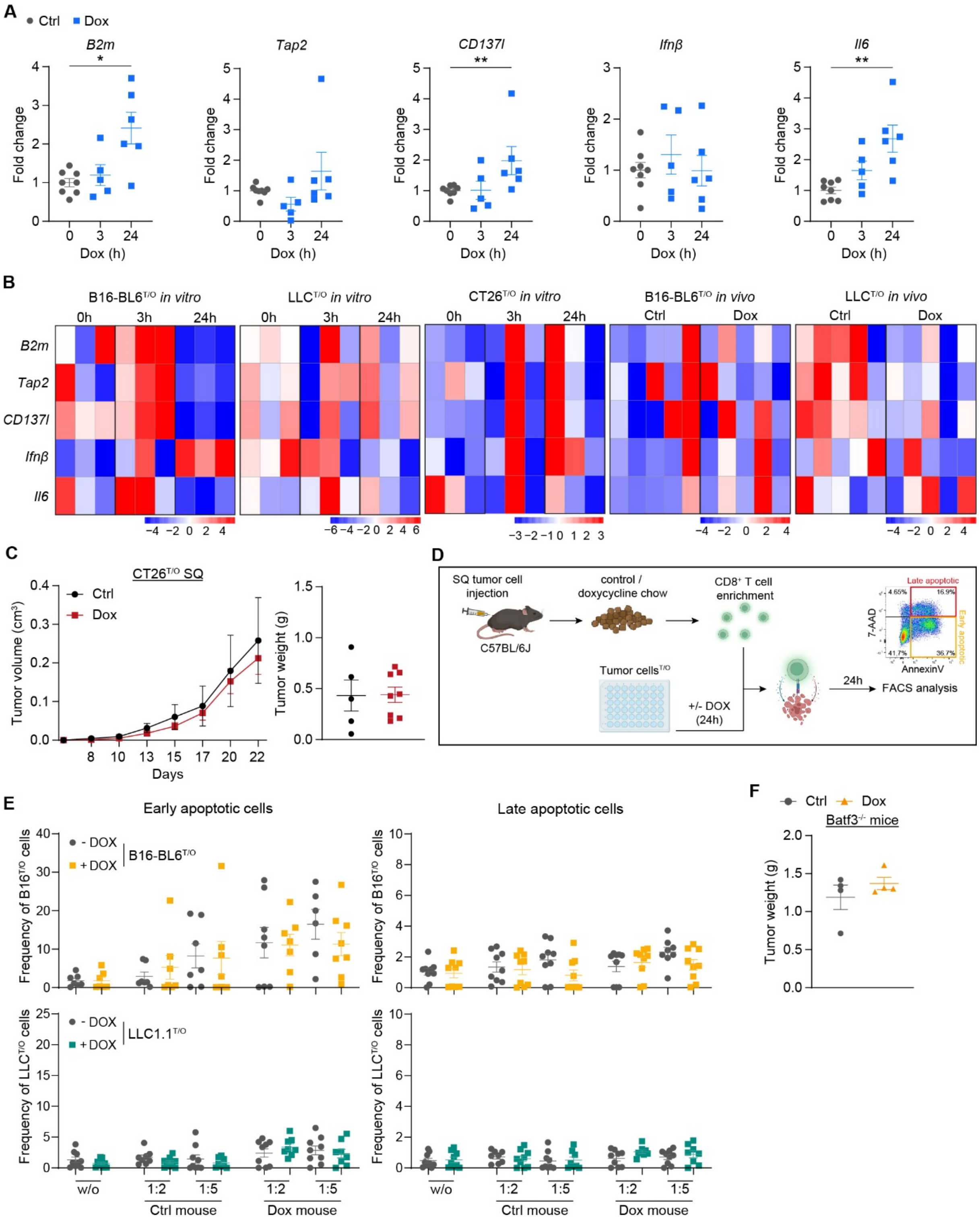

Supplementary Figure S6. c-Myb upregulation regulates intrinsic changes in MC38 tumor cells that control tumor growth by CD8⁺ T cells Part II. A-B, RT-qPCR data of indicated genes of *in vitro* Dox treated MC38$^{T/O}$ (**A**) and B16-BL6$^{T/O}$, LLC$^{T/O}$ and CT26$^{T/O}$ (**B**) cells. Data is normalized to GAPDH expression and relative to the untreated cells (**A**) or represented in a heat map (**B**). Data are presented from three independent experiments. Two right panels show analysis of respective genes in tumors of B16-BL6 and LLC tumor cells growth *in vivo*. **C,** Balb/c mice were subcutaneously injected with 5x10^{5} CT26$^{T/O}$ cells and were fed control (Ctrl) or doxycycline (Dox) containing chow for the duration of the experiment. Tumor growth and final tumor weight are shown. n=5-8. **D,** Experimental setup for killing assays. **E,** Killing assay of B16-BL6$^{T/O}$ (upper panels) and LLC$^{T/O}$ cells (lower panels), cultured and

untreated (- Dox) or treated with Dox (+ Dox) for 24 hours. Subsequently, enriched $CD8^+$ T cells from a spleen of, respectively, B16-BL6$^{T/O}$ or LLC$^{T/O}$ SQ tumor bearing C57BL/6J mice (Ctrl or Dox treated) were added in different ratios (tumor cell: $CD8^+$ T cell ratio 1:2 / 1:5). After 24 hours of co-culture early (AnnexinV$^+$ 7-AAD$^-$) and late (AnnexinV$^+$ 7-AAD$^+$) apoptotic tumor cells were assessed by Flow Cytometry. Data is presented as percentage of total tumor cells. w/o: tumor cells cultured without $CD8^+$ T cells. Data are presented from three independent experiments. **F,** Batf3$^{-/-}$ mice were subcutaneously injected with MC38$^{T/O}$ cells and were fed control (Ctrl) or doxycycline (Dox) containing chow for the duration of the experiment. Final tumor weight is shown. n=4. *p<0.05, **p<0.01.

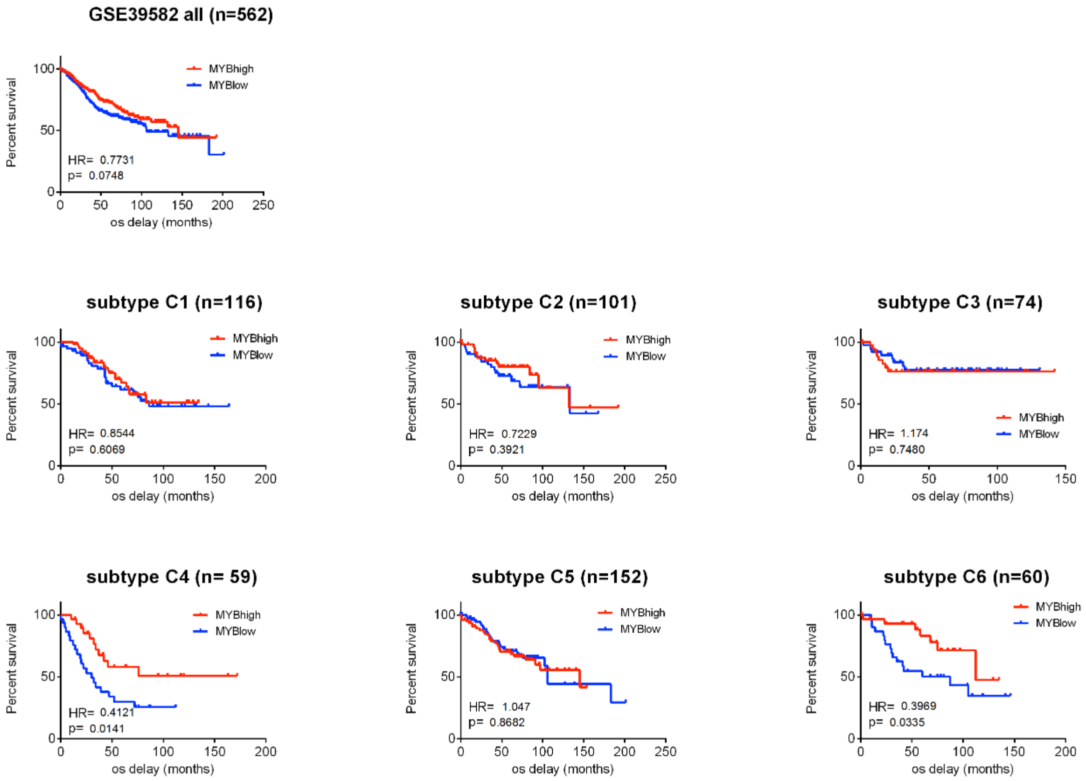

Supplementary Figure S7. c-Myb is a prognostic biomarker for specific molecular subtypes of CRC patients. Survival analysis of total 562 patients in GSE39582 cohort (19). Graphs show overall survival time of patients stratified according to the median MYB expression, top panel shows all patients, panels bellow show patients of specific molecular subtypes classified by Marisa et al. 2013. HR, hazard ratio, p, log-rank test.

Supplementary Table 1

Cloning vectors (Addgene)

Plasmid	# (Addgene)	Type of vector	Specifics
pMuLE ENTR SV40 eGFP L5-L2	62144	Entry vector	eGFP under SV40 promoter
pMuLE ENTR CMV/TO L1-R5	62099	Entry vector	TET-inducible CMV promoter
pMuLE Lenti Dest Luc2	62179	Destination vector	Firefly luciferase under PGK promoter
pMuLE Lenti Dest Neo	62178	Destination vector	Neomycin under PGK promoter

Supplementary Table 2

Flow cytometry antibodies

Antigen	Fluorophore	Clone	Company
CD45	APC-Cy7	30-F11	Biolegend
CD11b	BV510	M1/70	Biolegend
CD3e	PE-Cy7	145-2C11	Biolegend
PD1	PerCP-Cy5.5	29F.1A12	Biolegend
CD4	BV650	RM4-5	Biolegend
CD8	BV605	53-6.7	Biolegend
CD11c	PE-Cy7	N418	Biolegend
Ly6G	PerCP-Cy5.5	1A8	Biolegend
CD206	BV650	C068C2	Biolegend
MHCII	AF700	M5/114.15.2	Biolegend
CD64	BV421	X54-5/7.1	Biolegend
CD103	BV605	2E7	Biolegend
Siglec F	PE-CF594	E50-2440	BD
Perforin*	PE	S16009A	Biolegend
Granzyme B*	AF647	GB11	Biolegend

*Intracellular staining antibodies

Primers (5'-3') used for qPCR analysis:

B2M Fw CACCCGCCTCACATTGAAATC, **B2M Rv** TTGATCACATGTCTCGATCCCA;

CCR2 Fw GCAAGTTCAGCTGCCTGCAAA, **CCR2 Rv** GTATGCCGTGGATGAACTGAGGT;

CD137L Fw AAAAACCAAGCATCGTTGTGC, **CD137L Rv** GTAGAGCCCGGGACTGTCTA;

GAPDH Fw CATGTTCCAGTATGACTCCACTC, **GAPDH Rv** GGCCTCACCCCATTTGATGT;

IFNβ Fw GCCTTTGCCATCCAAGAGATGC, **IFNβ Rv** ACACTGTCTGCTGGTGGAGTTC;

IL6 Fw GTGGAAATGAGAAAAGAGTTGTGC, **IL6 Rv** ACCAGAGGAAATTTTCAATAGGC;

TAP2 Fw CTGTGAGGACGCTCAAGTGAT, **TAP2 Rv** CCCTTTTTCCCCGATTTCTGTG.

CITED2 fw GCAAAGACGGAAGGACTGGA, **CITED2 rev** CGTAGTGTATGTGCTCGCCC;

RAD54B fw GCAGCATCAAGACAACGACG, **RAD54B rev** TGAACTGGGGTACCAGTGAGA;

DAAM1 fw GCCTGGAAGCTCAGCTCAAA, **DAAM1 rev** GTCGAACACCTCTCCTGAGC;

CALM2 fw CCGTGTGTTTGATAAGGATGGCA, **CALM2 rev** GTAGTTTACCTGACCGTCCCC;

RAB31 fw GCCCAGAAATAGCCGCCTTG, **RAB31 rev** GGTCATGAAAGATGCCCCAATAGT;

LCP1 fw GGAACCGAGAGCTGTAAGGC, **LCP1 rev** ACTGCTTTAGGTGACAGACTTGAAT

c-Myb fw CATTTGATGGGGTTTGGGCA, **c-Myb rev** AGGATAGGGAACGTGACTGGA;

Supplementary Table 3

Pathway enrichment analysis using pathDIP (18) version 4.1 (http://ophid.utoronto.ca/pathDIP). 87 DEGs were used as input for querying all data sources, extended pathways and using default parameters. Significantly enriched pathways with more than 35 of the input DEGs present were further considered, as highlighted in Fig. 5b.

Pathway Source	Pathway Name	p-value	q-value (FDR: BH-method)	q-value (Bonferroni)
WikiPathways	Macrophage markers	5.70E-41	1.63E-37	1.63E-37
WikiPathways	TYROBP Causal Network	5.14E-38	7.34E-35	1.47E-34
REACTOME	Cross-presentation of particulate exogenous antigens (phagosomes)	1.36E-36	1.29E-33	3.87E-33
ACSN2	NO ROS PRODUCTION	7.59E-33	5.43E-30	2.17E-29
REACTOME	MyD88 deficiency (TLR2/4)	3.87E-31	2.21E-28	1.11E-27
KEGG	Pertussis	1.98E-30	9.44E-28	5.66E-27
REACTOME	IRAK4 deficiency (TLR2/4)	2.53E-29	1.03E-26	7.24E-26
KEGG	Asthma	7.91E-29	2.51E-26	2.26E-25
REACTOME	Interleukin-10 signaling	7.21E-29	2.58E-26	2.06E-25
KEGG	Intestinal immune network for IgA production	1.62E-28	4.62E-26	4.62E-25
KEGG	Staphylococcus aureus infection	2.72E-28	7.07E-26	7.77E-25
KEGG	Phagosome	8.97E-28	2.14E-25	2.56E-24
WikiPathways	Microglia Pathogen Phagocytosis	2.97E-27	6.52E-25	8.48E-24
WikiPathways	Allograft Rejection	3.34E-27	6.81E-25	9.54E-24
WikiPathways	Fibrin Complement Receptor 3 Signaling	7.20E-27	1.37E-24	2.06E-23
KEGG	Hematopoietic cell lineage	1.13E-25	2.01E-23	3.22E-22
KEGG	Leishmaniasis	1.57E-25	2.64E-23	4.49E-22
ACSN2	MARKERS DC	1.28E-24	2.03E-22	3.65E-21
KEGG	Allograft rejection	2.00E-24	3.01E-22	5.71E-21
REACTOME	ER-Phagosome	3.82E-24	5.46E-22	1.09E-20
REACTOME	Translocation of ZAP-70 to Immunological synapse	5.65E-24	7.69E-22	1.62E-20
KEGG	Inflammatory bowel disease (IBD)	7.37E-24	9.57E-22	2.11E-20
KEGG	Complement and coagulation cascades	1.37E-23	1.70E-21	3.90E-20
KEGG	Rheumatoid arthritis	1.54E-23	1.84E-21	4.40E-20
KEGG	NF-kappa B signaling	1.97E-23	2.25E-21	5.63E-20
WikiPathways	Oxidative Damage	4.91E-23	5.40E-21	1.40E-19
KEGG	Antigen processing and presentation	5.30E-23	5.61E-21	1.51E-19
KEGG	Type I diabetes mellitus	1.82E-22	1.86E-20	5.21E-19
WikiPathways	Platelet-mediated interactions with vascular and circulating cells	2.28E-22	2.25E-20	6.53E-19
REACTOME	Phosphorylation of CD3 and TCR zeta chains	2.45E-22	2.34E-20	7.01E-19
KEGG	Viral myocarditis	2.62E-22	2.41E-20	7.48E-19
KEGG	Graft-versus-host disease	3.93E-22	3.41E-20	1.12E-18
KEGG	Osteoclast differentiation	3.81E-22	3.41E-20	1.09E-18
KEGG	Tuberculosis	1.02E-21	8.58E-20	2.92E-18
KEGG	Autoimmune thyroid disease	1.48E-21	1.21E-19	4.23E-18
WikiPathways	Toll-like Receptor Signaling	1.75E-21	1.39E-19	5.01E-18
KEGG	Chagas disease (American trypanosomiasis)	1.82E-21	1.41E-19	5.21E-18
WikiPathways	Human Complement System	2.47E-21	1.86E-19	7.06E-18
REACTOME	PD-1 signaling	3.08E-21	2.26E-19	8.81E-18
WikiPathways	Regulation of toll-like receptor signaling	4.94E-21	3.53E-19	1.41E-17
WikiPathways	Interactions between immune cells and microRNAs in tumor microenvironment	8.40E-21	5.85E-19	2.40E-17
BioCarta	lck and fyn tyrosine kinases in initiation of tcr activation	9.96E-21	6.78E-19	2.85E-17
PID	IL12-mediated signaling events	1.20E-20	7.96E-19	3.42E-17
REACTOME	Neutrophil degranulation	1.41E-20	9.14E-19	4.02E-17
REACTOME	Diseases associated with the TLR signaling cascade	1.59E-20	1.01E-18	4.55E-17
REACTOME	Diseases of Immune System	1.59E-20	1.01E-18	4.55E-17
ACSN2	FC RECEPTORS	1.80E-20	1.10E-18	5.16E-17
KEGG	Toll-like receptor signaling	2.46E-20	1.46E-18	7.02E-17
BioCarta	classical complement	3.13E-20	1.76E-18	8.96E-17
REACTOME	Immunoregulatory interactions between a Lymphoid and a non-Lymphoid cell	3.01E-20	1.76E-18	8.61E-17
REACTOME	Cell surface interactions at the vascular wall	3.10E-20	1.77E-18	8.86E-17
ACSN2	RECRUITMENT OF IMMUNE CELLS	3.94E-20	2.17E-18	1.13E-16
KEGG	Cytokine-cytokine receptor interaction	7.06E-20	3.81E-18	2.02E-16
REACTOME	Interferon gamma signaling	9.94E-20	5.26E-18	2.84E-16
KEGG	Legionellosis	1.24E-19	6.42E-18	3.53E-16
ACSN2	IMMUNOSUPPRESSIVE CYTOKINE	1.79E-19	9.14E-18	5.12E-16

**KEGG**	Systemic lupus erythematosus	2.74E-19	1.37E-17	7.82E-16
**BioCarta**	activation of csk by camp-dependent protein kinase inhibits signaling through the t cell receptor	4.08E-19	2.01E-17	1.17E-15
**KEGG**	Th17 cell differentiation	4.91E-19	2.38E-17	1.40E-15
**KEGG**	Toxoplasmosis	5.49E-19	2.62E-17	1.57E-15
**REACTOME**	Antigen processing-Cross presentation	7.58E-19	3.55E-17	2.17E-15
**Spike**	NFkB Signaling Network	8.73E-19	4.02E-17	2.49E-15
**WikiPathways**	Inflammatory Response	1.14E-18	5.15E-17	3.24E-15
**KEGG**	Cell adhesion molecules (CAMs)	1.60E-18	7.15E-17	4.58E-15
**WikiPathways**	Spinal Cord Injury	2.41E-18	1.03E-16	6.89E-15
**REACTOME**	ROS and RNS production in phagocytes	2.40E-18	1.04E-16	6.86E-15
**REACTOME**	Regulation of TLR by endogenous ligand	2.39E-18	1.05E-16	6.84E-15
**KEGG**	Herpes simplex infection	2.74E-18	1.15E-16	7.84E-15
**WikiPathways**	Ebola Virus Pathway on Host	4.13E-18	1.71E-16	1.18E-14
**REACTOME**	Toll-like Receptor Cascades	7.59E-18	3.10E-16	2.17E-14
**WikiPathways**	Viral Acute Myocarditis	7.73E-18	3.11E-16	2.21E-14
**WikiPathways**	Complement and Coagulation Cascades	9.26E-18	3.68E-16	2.65E-14
**REACTOME**	Signaling by Interleukins	2.33E-17	9.12E-16	6.66E-14
**REACTOME**	Innate Immune System	2.70E-17	1.04E-15	7.71E-14
**WikiPathways**	IL1 and megakaryocytes in obesity	3.07E-17	1.17E-15	8.77E-14
**PID**	IL12 signaling mediated by STAT4	3.72E-17	1.38E-15	1.06E-13
**WikiPathways**	Complement Activation	3.70E-17	1.39E-15	1.06E-13
**REACTOME**	Cytokine Signaling in Immune system	4.02E-17	1.47E-15	1.15E-13
**KEGG**	Leukocyte transendothelial migration	4.58E-17	1.66E-15	1.31E-13
**KEGG**	Epstein-Barr virus infection	5.86E-17	2.09E-15	1.67E-13
**REACTOME**	Interleukin-4 and Interleukin-13 signaling	7.66E-17	2.70E-15	2.19E-13
**WikiPathways**	Cells and Molecules involved in local acute inflammatory response	1.15E-16	4.01E-15	3.29E-13
**REACTOME**	Hemostasis	1.20E-16	4.12E-15	3.42E-13
**KEGG**	Th1 and Th2 cell differentiation	1.64E-16	5.57E-15	4.68E-13
**PID**	Endogenous TLR signaling	1.70E-16	5.73E-15	4.87E-13
**REACTOME**	Generation of second messenger molecules	2.12E-16	7.04E-15	6.06E-13
**KEGG**	Amoebiasis	2.79E-16	9.16E-15	7.97E-13
**systems-biology.org**	Toll-Like receptor signaling network	2.88E-16	9.34E-15	8.22E-13
**PID**	amb2 Integrin signaling	3.38E-16	1.09E-14	9.66E-13
**PID**	Beta2 integrin cell surface interactions	3.70E-16	1.18E-14	1.06E-12
**INOH**	CD4 T cell receptor signaling-JNK cascade	7.61E-16	2.39E-14	2.17E-12
**REACTOME**	Toll Like Receptor 4 (TLR4) Cascade	8.48E-16	2.63E-14	2.42E-12
**NetPath**	BCR	1.59E-15	4.87E-14	4.53E-12
**WikiPathways**	LTF danger signal response	1.61E-15	4.88E-14	4.59E-12
**REACTOME**	RHO GTPases Activate NADPH Oxidases	1.67E-15	5.02E-14	4.77E-12
**ACSN2**	SMAC	1.70E-15	5.05E-14	4.85E-12
**REACTOME**	Costimulation by the CD28 family	1.86E-15	5.48E-14	5.32E-12
**PID**	Fc-epsilon receptor I signaling in mast cells	1.97E-15	5.75E-14	5.63E-12
**REACTOME**	Platelet activation, signaling and aggregation	2.81E-15	8.10E-14	8.02E-12
**KEGG**	Malaria	3.28E-15	9.39E-14	9.39E-12
**KEGG**	Kaposi sarcoma-associated herpesvirus infection	5.51E-15	1.56E-13	1.57E-11
**BioCarta**	ras-independent pathway in nk cell-mediated cytotoxicity	7.35E-15	2.06E-13	2.10E-11
**NetPath**	TCR	7.94E-15	2.20E-13	2.27E-11
**ACSN2**	CYTOKINES CHEMOKINES PRODUCTION	9.95E-15	2.73E-13	2.84E-11
**REACTOME**	Immune System	1.46E-14	3.96E-13	4.16E-11
**KEGG**	TNF signaling	1.62E-14	4.38E-13	4.64E-11
**REACTOME**	Detoxification of Reactive Oxygen Species	1.85E-14	4.94E-13	5.29E-11
**WikiPathways**	ApoE and miR-146 in inflammation and atherosclerosis	2.45E-14	6.47E-13	6.99E-11
**KEGG**	Platelet activation	2.80E-14	7.21E-13	8.01E-11
**PID**	TCR signaling in naïve CD4+ T cells	2.79E-14	7.25E-13	7.98E-11
**Panther_Pathway**	Inflammation mediated by chemokine and cytokine signaling	2.79E-14	7.31E-13	7.96E-11
**KEGG**	Influenza A	3.33E-14	8.50E-13	9.53E-11
**NetPath**	IL5	4.47E-14	1.13E-12	1.28E-10
**Spike**	TLR Signaling	4.80E-14	1.20E-12	1.37E-10
**PID**	TCR signaling in naïve CD8+ T cells	6.09E-14	1.51E-12	1.74E-10
**WikiPathways**	T-Cell antigen Receptor (TCR) Signaling	7.14E-14	1.76E-12	2.04E-10
**PID**	CXCR4-mediated signaling events	8.69E-14	2.12E-12	2.48E-10
**REACTOME**	GPVI-mediated activation cascade	9.14E-14	2.21E-12	2.61E-10
**PID**	IL23-mediated signaling events	9.74E-14	2.34E-12	2.78E-10
**KEGG**	Prion diseases	1.04E-13	2.48E-12	2.98E-10
**REACTOME**	Complement cascade	1.12E-13	2.64E-12	3.19E-10
**REACTOME**	Adaptive Immune System	1.24E-13	2.90E-12	3.54E-10
**KEGG**	Human cytomegalovirus infection	1.42E-13	3.29E-12	4.05E-10
**KEGG**	Natural killer cell mediated cytotoxicity	1.89E-13	4.36E-12	5.40E-10

**WikiPathways**	B Cell Receptor Signaling	2.44E-13	5.57E-12	6.96E-10
**PID**	Urokinase-type plasminogen activator (uPA) and uPAR-mediated signaling	3.12E-13	7.08E-12	8.93E-10
**REACTOME**	DAP12 signaling	4.00E-13	9.01E-12	1.14E-09
**REACTOME**	Trafficking and processing of endosomal TLR	4.20E-13	9.38E-12	1.20E-09
**ACSN2**	IMMUNOSTIMULATORY CYTOKINE	5.02E-13	1.11E-11	1.43E-09
**KEGG**	Fc gamma R-mediated phagocytosis	5.32E-13	1.17E-11	1.52E-09
**KEGG**	Human T-cell leukemia virus 1 infection	8.42E-13	1.84E-11	2.41E-09
**KEGG**	NOD-like receptor signaling	1.27E-12	2.76E-11	3.64E-09
**REACTOME**	Regulation of Complement cascade	1.52E-12	3.26E-11	4.33E-09
**REACTOME**	Chemokine receptors bind chemokines	1.85E-12	3.95E-11	5.29E-09
**ACSN2**	TREG MODULATORS	1.87E-12	3.97E-11	5.35E-09
**PID**	IL6-mediated signaling events	1.90E-12	4.00E-11	5.44E-09
**KEGG**	Primary immunodeficiency	2.01E-12	4.20E-11	5.76E-09
**PID**	IL27-mediated signaling events	2.07E-12	4.29E-11	5.92E-09
**KEGG**	AGE-RAGE signaling pathway in diabetic complications	2.16E-12	4.41E-11	6.17E-09
**BioCarta**	the co-stimulatory signal during t-cell activation	2.15E-12	4.41E-11	6.14E-09
**NetPath**	IL4	2.42E-12	4.91E-11	6.93E-09
**WikiPathways**	Type II interferon signaling (IFNG)	2.59E-12	5.18E-11	7.40E-09
**REACTOME**	Interleukin-3, Interleukin-5 and GM-CSF signaling	2.59E-12	5.20E-11	7.39E-09
**REACTOME**	CD28 dependent Vav1	2.75E-12	5.47E-11	7.87E-09
**INOH**	JAK STAT MolecularVariation 2	2.88E-12	5.69E-11	8.24E-09
**NetPath**	IL6	3.20E-12	6.26E-11	9.14E-09
**WikiPathways**	IL-3 Signaling	3.53E-12	6.86E-11	1.01E-08
**REACTOME**	Integrin cell surface interactions	3.65E-12	7.05E-11	1.04E-08
**KEGG**	Salmonella infection	5.19E-12	9.96E-11	1.48E-08
**INOH**	CD4 T cell receptor signaling	5.25E-12	1.00E-10	1.50E-08
**WikiPathways**	miRNAs involvement in the immune response in sepsis	5.49E-12	1.04E-10	1.57E-08
**BioCarta**	pertussis toxin-insensitive ccr5 signaling in macrophage	6.29E-12	1.17E-10	1.80E-08
**INOH**	CD4 T cell receptor signaling-NFkB cascade	6.27E-12	1.18E-10	1.79E-08
**REACTOME**	Platelet Adhesion to exposed collagen	6.36E-12	1.18E-10	1.82E-08
**REACTOME**	Interferon Signaling	1.18E-11	2.18E-10	3.38E-08
**WikiPathways**	Photodynamic therapy-induced NF-kB survival signaling	1.32E-11	2.41E-10	3.77E-08
**REACTOME**	FCERI mediated Ca+2 mobilization	1.70E-11	3.10E-10	4.86E-08
**REACTOME**	Regulation of signaling by CBL	1.81E-11	3.27E-10	5.17E-08
**WikiPathways**	AGE/RAGE	1.84E-11	3.32E-10	5.27E-08
**WikiPathways**	T-Cell antigen Receptor (TCR) pathway during Staphylococcus aureus infection	3.03E-11	5.39E-10	8.67E-08
**BioCarta**	t cell receptor signaling	3.02E-11	5.39E-10	8.62E-08
**KEGG**	T cell receptor signaling	3.55E-11	6.26E-10	1.01E-07
**PID**	BCR signaling	3.74E-11	6.55E-10	1.07E-07
**REACTOME**	Other semaphorin interactions	3.99E-11	6.96E-10	1.14E-07
**INOH**	Integrin	5.20E-11	9.00E-10	1.48E-07
**Panther_Pathway**	T cell activation	5.29E-11	9.10E-10	1.51E-07
**WikiPathways**	Lung fibrosis	5.34E-11	9.13E-10	1.52E-07
**REACTOME**	FCERI mediated MAPK activation	5.56E-11	9.45E-10	1.59E-07
**KEGG**	Chemokine signaling	6.55E-11	1.10E-09	1.87E-07
**ACSN2**	ACTIVATING CHECKPOINTS	6.54E-11	1.11E-09	1.87E-07
**WikiPathways**	Thymic Stromal LymphoPoietin (TSLP) Signaling	6.68E-11	1.12E-09	1.91E-07
**BioCarta**	fc epsilon receptor i signaling in mast cells	7.69E-11	1.28E-09	2.20E-07
**PID**	Syndecan-4-mediated signaling events	8.68E-11	1.43E-09	2.48E-07
**NetPath**	KitReceptor	9.68E-11	1.59E-09	2.77E-07
**KEGG**	B cell receptor signaling	1.01E-10	1.65E-09	2.89E-07
**WikiPathways**	Selective expression of chemokine receptors during T-cell polarization	1.23E-10	2.00E-09	3.51E-07
**KEGG**	Measles	1.66E-10	2.68E-09	4.74E-07
**WikiPathways**	Hematopoietic Stem Cell Differentiation	1.68E-10	2.70E-09	4.81E-07
**INOH**	CD4 T cell receptor signaling-ERK cascade	1.72E-10	2.75E-09	4.92E-07
**ACSN2**	MARKERS MACROPHAGE	1.85E-10	2.94E-09	5.29E-07
**REACTOME**	TCR signaling	2.02E-10	3.19E-09	5.77E-07
**WikiPathways**	Chemokine signaling	2.64E-10	4.14E-09	7.54E-07
**KEGG**	Pathogenic Escherichia coli infection	3.53E-10	5.51E-09	1.01E-06
**WikiPathways**	Pathogenic Escherichia coli infection	3.53E-10	5.51E-09	1.01E-06
**INOH**	JAK STAT MolecularVariation 1	4.21E-10	6.50E-09	1.20E-06
**REACTOME**	CD28 co-stimulation	4.35E-10	6.69E-09	1.24E-06
**REACTOME**	DAP12 interactions	4.53E-10	6.93E-09	1.30E-06
**WikiPathways**	Simplified Depiction of MYD88 Distinct Input-Output	5.06E-10	7.70E-09	1.45E-06
**REACTOME**	Extracellular matrix organization	5.59E-10	8.45E-09	1.60E-06
**BioCarta**	role of mef2d in t-cell apoptosis	5.97E-10	8.98E-09	1.71E-06
**BioCarta**	hiv-1 defeats host-mediated resistance by cem15	6.30E-10	9.42E-09	1.80E-06

**REACTOME**	Fcgamma receptor (FCGR) dependent phagocytosis	7.31E-10	1.09E-08	2.09E-06
**PID**	IL8- and CXCR2-mediated signaling events	9.49E-10	1.41E-08	2.71E-06
**SIGNOR2.0**	SIGNOR-TCA	1.07E-09	1.57E-08	3.05E-06
**KEGG**	C-type lectin receptor signaling	1.09E-09	1.59E-08	3.11E-06
**PID**	PDGFR-beta signaling	1.09E-09	1.59E-08	3.11E-06
**REACTOME**	Dectin-2 family	1.15E-09	1.67E-08	3.30E-06
**WikiPathways**	RANKL/RANK (Receptor activator of NFKB (ligand)) Signaling	1.27E-09	1.83E-08	3.63E-06
**SMPDB**	Fc Epsilon Receptor I Signaling in Mast Cells	1.36E-09	1.96E-08	3.90E-06
**Panther_Pathway**	B cell activation	1.47E-09	2.10E-08	4.20E-06
**REACTOME**	Signal regulatory protein family interactions	1.65E-09	2.33E-08	4.71E-06
**Panther_Pathway**	Toll receptor signaling	1.64E-09	2.34E-08	4.69E-06
**REACTOME**	Nef and signal transduction	1.93E-09	2.72E-08	5.52E-06
**KEGG**	Fc epsilon RI signaling	2.23E-09	3.12E-08	6.36E-06
**ACSN2**	MARKERS MDSC	2.40E-09	3.34E-08	6.85E-06
**PID**	Alpha-synuclein signaling	2.68E-09	3.72E-08	7.67E-06
**KEGG**	Proteoglycans in cancer	2.98E-09	4.11E-08	8.50E-06
**stke**	PI3K Class IB Pathway in Neutrophils	3.05E-09	4.19E-08	8.72E-06
**REACTOME**	Binding and entry of HIV virion	3.32E-09	4.54E-08	9.49E-06
**REACTOME**	Initial triggering of complement	3.73E-09	5.07E-08	1.06E-05
**PID**	IL4-mediated signaling events	3.93E-09	5.32E-08	1.12E-05
**REACTOME**	Platelet degranulation	3.95E-09	5.33E-08	1.13E-05
**stke**	Toll-Like Receptor Pathway	4.01E-09	5.39E-08	1.15E-05
**WikiPathways**	IL-10 Anti-inflammatory Signaling Pathway	4.04E-09	5.40E-08	1.16E-05
**REACTOME**	Downstream TCR signaling	4.45E-09	5.91E-08	1.27E-05
**REACTOME**	MHC class II antigen presentation	4.61E-09	6.11E-08	1.32E-05
**BioCarta**	toll-like receptor	4.71E-09	6.20E-08	1.35E-05
**ACSN2**	INFLAMMATORY SIGNALING	4.86E-09	6.37E-08	1.39E-05
**KEGG**	Fluid shear stress and atherosclerosis	5.88E-09	7.67E-08	1.68E-05
**IPAVS**	JAK STAT pathway in postconditioning ischemia	6.55E-09	8.50E-08	1.87E-05
**WikiPathways**	IL-2 Signaling	1.06E-08	1.38E-07	3.04E-05
**PID**	GMCSF-mediated signaling events	1.16E-08	1.50E-07	3.33E-05
**IPAVS**	GP130 JAK STAT	1.18E-08	1.51E-07	3.36E-05
**PID**	Beta1 integrin cell surface interactions	1.38E-08	1.76E-07	3.94E-05
**REACTOME**	Signaling by SCF-KIT	1.45E-08	1.84E-07	4.14E-05
**systems-biology.org**	Macrophage Map Update ver2 re	1.75E-08	2.22E-07	5.01E-05
**INOH**	IL-10 signaling	1.83E-08	2.31E-07	5.23E-05
**PID**	Beta3 integrin cell surface interactions	1.84E-08	2.31E-07	5.26E-05
**WikiPathways**	IL-5 Signaling	1.85E-08	2.31E-07	5.30E-05
**WikiPathways**	TNF related weak inducer of apoptosis (TWEAK) Signaling	1.92E-08	2.38E-07	5.48E-05
**WikiPathways**	IL-4 Signaling	2.55E-08	3.16E-07	7.29E-05
**SMPDB**	Thyroid hormone synthesis	2.75E-08	3.39E-07	7.87E-05
**WikiPathways**	Non-genomic actions of 1,25 dihydroxyvitamin D3	2.92E-08	3.57E-07	8.36E-05
**REACTOME**	Creation of C4 and C2 activators	2.92E-08	3.59E-07	8.35E-05
**REACTOME**	Signaling by the B Cell Receptor (BCR)	3.06E-08	3.72E-07	8.75E-05
**PID**	Osteopontin-mediated events	3.39E-08	4.11E-07	9.69E-05
**NetPath**	IL2	3.56E-08	4.29E-07	1.02E-04
**PID**	Thromboxane A2 receptor signaling	4.32E-08	5.17E-07	1.24E-04
**ACSN2**	DANGER SIGNAL	4.32E-08	5.19E-07	1.23E-04
**SignaLink2.0**	JAK/STAT(core)	4.44E-08	5.29E-07	1.27E-04
**WikiPathways**	Control of immune tolerance by vasoactive intestinal peptide	5.18E-08	6.11E-07	1.48E-04
**REACTOME**	The role of Nef in HIV-1 replication and disease pathogenesis	5.16E-08	6.12E-07	1.47E-04
**REACTOME**	Response to elevated platelet cytosolic Ca2+	5.36E-08	6.31E-07	1.53E-04
**NetPath**	TSLP	5.51E-08	6.43E-07	1.57E-04
**WikiPathways**	Signal transduction through IL1R	5.49E-08	6.43E-07	1.57E-04
**REACTOME**	Interleukin-2 family signaling	5.72E-08	6.64E-07	1.63E-04
**REACTOME**	MyD88:MAL(TIRAP) cascade initiated on plasma membrane	5.97E-08	6.91E-07	1.71E-04
**REACTOME**	Toll Like Receptor TLR6:TLR2 Cascade	5.97E-08	6.91E-07	1.71E-04
**KEGG**	Acute myeloid leukemia	6.16E-08	7.07E-07	1.76E-04
**REACTOME**	PECAM1 interactions	6.30E-08	7.17E-07	1.80E-04
**SIGNOR2.0**	SIGNOR-TLR	6.28E-08	7.18E-07	1.79E-04
**PID**	Canonical NF-kappaB	6.99E-08	7.93E-07	2.00E-04
**REACTOME**	Interferon alpha/beta signaling	7.03E-08	7.94E-07	2.01E-04
**REACTOME**	FCGR activation	7.67E-08	8.63E-07	2.19E-04
**KEGG**	African trypanosomiasis	9.08E-08	1.02E-06	2.60E-04
**REACTOME**	Regulation of actin dynamics for phagocytic cup formation	9.18E-08	1.02E-06	2.62E-04
**PID**	Class I PI3K signaling events	9.22E-08	1.03E-06	2.64E-04
**WikiPathways**	Vitamin D Receptor	1.14E-07	1.27E-06	3.26E-04
**KEGG**	Epithelial cell signaling in Helicobacter pylori infection	1.15E-07	1.27E-06	3.30E-04
**PID**	Alpha4 beta1 integrin signaling events	1.20E-07	1.31E-06	3.42E-04

**REACTOME**	Degradation of the extracellular matrix	1.57E-07	1.72E-06	4.48E-04
**REACTOME**	Toll Like Receptor 2 (TLR2) Cascade	1.60E-07	1.75E-06	4.58E-04
**REACTOME**	Toll Like Receptor TLR1:TLR2 Cascade	1.60E-07	1.75E-06	4.58E-04
**PID**	IL1-mediated signaling events	1.64E-07	1.77E-06	4.68E-04
**BioCarta**	antigen processing and presentation	1.76E-07	1.90E-06	5.03E-04
**KEGG**	Pathways in cancer	1.77E-07	1.90E-06	5.05E-04
**WikiPathways**	IL-6 signaling	1.96E-07	2.09E-06	5.59E-04
**REACTOME**	RHO GTPases Activate WASPs and WAVEs	1.97E-07	2.10E-06	5.62E-04
**BioCarta**	bcr signaling	2.00E-07	2.12E-06	5.71E-04
**REACTOME**	Classical antibody-mediated complement activation	2.29E-07	2.42E-06	6.54E-04
**KEGG**	Regulation of actin cytoskeleton	2.32E-07	2.44E-06	6.62E-04
**REACTOME**	Interleukin-9 signaling	2.46E-07	2.58E-06	7.03E-04
**ACSN2**	EMT REGULATORS	2.52E-07	2.64E-06	7.20E-04
**REACTOME**	Nucleotide-binding domain, leucine rich repeat containing receptor (NLR) signaling	2.55E-07	2.66E-06	7.29E-04
**WikiPathways**	Cytokines and Inflammatory Response	2.62E-07	2.72E-06	7.48E-04
**stke**	Interleukin 1 (IL-1) Pathway	2.75E-07	2.85E-06	7.87E-04
**NetPath**	RAGE	2.79E-07	2.88E-06	7.97E-04
**ACSN2**	ECM	2.87E-07	2.95E-06	8.21E-04
**WikiPathways**	ncRNAs involved in STAT3 signaling in hepatocellular carcinoma	2.92E-07	2.99E-06	8.34E-04
**REACTOME**	TRIF-mediated programmed cell death	3.50E-07	3.57E-06	9.99E-04
**PID**	Validated transcriptional targets of AP1 family members Fra1 and Fra2	3.52E-07	3.58E-06	1.01E-03
**REACTOME**	Interleukin-21 signaling	3.67E-07	3.72E-06	1.05E-03
**REACTOME**	Interleukin-2 signaling	3.93E-07	3.97E-06	1.12E-03
**REACTOME**	C-type lectin receptors (CLRs)	4.37E-07	4.40E-06	1.25E-03
**REACTOME**	Fc epsilon receptor (FCERI) signaling	4.45E-07	4.46E-06	1.27E-03
**PID**	Regulation of p38-alpha and p38-beta	4.55E-07	4.54E-06	1.30E-03
**KEGG**	Cytosolic DNA-sensing	4.59E-07	4.57E-06	1.31E-03
**WikiPathways**	Apoptosis	4.78E-07	4.74E-06	1.36E-03
**PID**	Signaling events mediated by PTP1B	4.85E-07	4.80E-06	1.39E-03
**BioCarta**	lectin induced complement	5.08E-07	5.00E-06	1.45E-03
**BioCarta**	nfkb activation by nontypeable hemophilus influenzae	5.12E-07	5.03E-06	1.46E-03
**BioCarta**	nf-kb signaling	5.45E-07	5.34E-06	1.56E-03
**KEGG**	Apoptosis	5.84E-07	5.70E-06	1.67E-03
**REACTOME**	Signaling by Receptor Tyrosine Kinases	6.76E-07	6.57E-06	1.93E-03
**REACTOME**	Signaling by VEGF	7.35E-07	7.09E-06	2.10E-03
**KEGG**	Human immunodeficiency virus 1 infection	7.32E-07	7.10E-06	2.09E-03
**WikiPathways**	TLR4 Signaling and Tolerance	7.83E-07	7.54E-06	2.24E-03
**WikiPathways**	Peptide GPCRs	8.15E-07	7.82E-06	2.33E-03
**REACTOME**	Regulation of KIT signaling	8.26E-07	7.89E-06	2.36E-03
**REACTOME**	Interleukin-27 signaling	8.35E-07	7.96E-06	2.39E-03
**WikiPathways**	Prolactin Signaling	8.44E-07	8.01E-06	2.41E-03
**NetPath**	Oncostatin M	8.88E-07	8.41E-06	2.54E-03
**PID**	Atypical NF-kappaB	9.20E-07	8.68E-06	2.63E-03
**PID**	IL2 signaling events mediated by STAT5	1.11E-06	1.04E-05	3.18E-03
**WikiPathways**	PDGF	1.11E-06	1.04E-05	3.17E-03
**WikiPathways**	Interferon type I signaling	1.28E-06	1.20E-05	3.67E-03
**REACTOME**	Signal Transduction	1.32E-06	1.23E-05	3.78E-03
**REACTOME**	Caspase activation via Death Receptors in the presence of ligand	1.46E-06	1.35E-05	4.17E-03
**PID**	Validated targets of C-MYC transcriptional repression	1.56E-06	1.44E-05	4.46E-03
**REACTOME**	Interleukin-35 Signalling	1.60E-06	1.48E-05	4.58E-03
**PID**	RAC1 signaling	1.72E-06	1.58E-05	4.91E-03
**PharmGKB**	Platelet Aggregation Inhibitor Pathway Pharmacodynamics	1.85E-06	1.70E-05	5.30E-03
**KEGG**	JAK-STAT signaling	1.86E-06	1.70E-05	5.32E-03
**REACTOME**	Binding and Uptake of Ligands by Scavenger Receptors	1.88E-06	1.71E-05	5.36E-03
**REACTOME**	Peptide ligand-binding receptors	1.89E-06	1.71E-05	5.39E-03
**KEGG**	Hepatitis B	1.95E-06	1.76E-05	5.56E-03
**BioCarta**	signal transduction through il1r	2.00E-06	1.80E-05	5.71E-03
**REACTOME**	VEGFA-VEGFR2 Pathway	1.99E-06	1.80E-05	5.69E-03
**REACTOME**	Interleukin-1 processing	2.06E-06	1.83E-05	5.87E-03
**NetPath**	Prolactin	2.05E-06	1.83E-05	5.86E-03
**IPAVS**	STAT signaling	2.05E-06	1.83E-05	5.85E-03
**NetPath**	IL3	2.10E-06	1.87E-05	6.01E-03
**PID**	EPO signaling	2.19E-06	1.94E-05	6.27E-03
**WikiPathways**	Oncostatin M Signaling	2.41E-06	2.12E-05	6.88E-03
**BioCarta**	il-10 anti-inflammatory signaling	2.75E-06	2.41E-05	7.85E-03
**INOH**	JAK STAT pathway and regulation	2.94E-06	2.58E-05	8.40E-03
**systems-biology.org**	EGFR signaling for RTKC	3.17E-06	2.77E-05	9.06E-03
**NetPath**	RANKL	3.37E-06	2.94E-05	9.64E-03
**REACTOME**	Collagen degradation	4.16E-06	3.61E-05	1.19E-02

**WikiPathways**	Overview of nanoparticle effects	4.21E-06	3.65E-05	1.20E-02
**ACSN2**	IMMUNOSTIMULATORY CORE	4.31E-06	3.71E-05	1.23E-02
**REACTOME**	Endosomal/Vacuolar	4.30E-06	3.71E-05	1.23E-02
**ACSN2**	CELL MATRIX ADHESIONS	4.42E-06	3.79E-05	1.26E-02
**ACSN2**	TCR SIGNALING	4.48E-06	3.84E-05	1.28E-02
**Panther_Pathway**	Apoptosis signaling	4.66E-06	3.97E-05	1.33E-02
**NetPath**	IL1	4.79E-06	4.08E-05	1.37E-02
**WikiPathways**	Senescence and Autophagy in Cancer	4.83E-06	4.09E-05	1.38E-02
**KEGG**	IL-17 signaling	5.17E-06	4.37E-05	1.48E-02
**PID**	Ephrin B reverse signaling	5.19E-06	4.37E-05	1.48E-02
**WikiPathways**	T-Cell Receptor and Co-stimulatory Signaling	5.19E-06	4.37E-05	1.48E-02
**REACTOME**	MyD88 dependent cascade initiated on endosome	5.40E-06	4.53E-05	1.54E-02
**REACTOME**	Toll Like Receptor 7/8 (TLR7/8) Cascade	5.40E-06	4.53E-05	1.54E-02
**BioCarta**	il 2 signaling	5.80E-06	4.83E-05	1.66E-02
**SIGNOR2.0**	SIGNOR-NFKBC	5.85E-06	4.86E-05	1.67E-02
**Panther_Pathway**	Interleukin signaling	5.96E-06	4.92E-05	1.70E-02
**KEGG**	Shigellosis	5.94E-06	4.92E-05	1.70E-02
**REACTOME**	Interleukin-6 signaling	6.36E-06	5.24E-05	1.82E-02
**REACTOME**	Interleukin-1 family signaling	6.40E-06	5.26E-05	1.83E-02
**WikiPathways**	IL-7 Signaling	6.43E-06	5.27E-05	1.84E-02
**PID**	Downstream signaling in naïve CD8+ T cells	6.86E-06	5.60E-05	1.96E-02
**WikiPathways**	Hepatitis C and Hepatocellular Carcinoma	6.96E-06	5.67E-05	1.99E-02
**WikiPathways**	Nucleotide-binding Oligomerization Domain (NOD)	7.00E-06	5.68E-05	2.00E-02
**PharmGKB**	Leukotriene modifiers pathway Pharmacodynamics	7.05E-06	5.71E-05	2.02E-02
**REACTOME**	MyD88 deficiency (TLR5)	7.05E-06	5.71E-05	2.02E-02
**REACTOME**	Activation of C3 and C5	8.24E-06	6.63E-05	2.35E-02
**WikiPathways**	Kit receptor signaling	8.27E-06	6.64E-05	2.36E-02
**INOH**	TLR ECSIT MEKK1 JNK	8.36E-06	6.69E-05	2.39E-02
**ACSN2**	MITOCHONDRIA OXIDATIVE STRESS	8.56E-06	6.83E-05	2.45E-02
**INOH**	IL-4 signaling	8.71E-06	6.93E-05	2.49E-02
**WikiPathways**	VEGFA-VEGFR2 Signaling	8.98E-06	7.13E-05	2.57E-02
**REACTOME**	G alpha (i) signalling events	9.26E-06	7.33E-05	2.65E-02
**ACSN2**	MIRNA TF IMMUNOSUPPRESSIVE	1.04E-05	8.18E-05	2.96E-02
**ACSN2**	MIRNA TF IMMUNOSTIMULATORY	1.12E-05	8.80E-05	3.19E-02
**PID**	Beta5 beta6 beta7 and beta8 integrin cell surface interactions	1.12E-05	8.80E-05	3.19E-02
**REACTOME**	PI5P, PP2A and IER3 Regulate PI3K/AKT Signaling	1.13E-05	8.82E-05	3.22E-02
**WikiPathways**	Interleukin-11 Signaling	1.15E-05	9.00E-05	3.29E-02
**INOH**	IL-6 signaling	1.19E-05	9.27E-05	3.40E-02
**PID**	Glypican 1 network	1.20E-05	9.33E-05	3.44E-02
**REACTOME**	CLEC7A/inflammasome	1.20E-05	9.34E-05	3.44E-02
**WikiPathways**	Leptin signaling	1.29E-05	9.97E-05	3.69E-02
**REACTOME**	Constitutive Signaling by Aberrant PI3K in Cancer	1.32E-05	1.02E-04	3.77E-02
**Panther_Pathway**	Integrin signalling	1.34E-05	1.03E-04	3.83E-02
**WikiPathways**	White fat cell differentiation	1.37E-05	1.05E-04	3.93E-02
**stke**	B Cell Antigen Receptor	1.39E-05	1.06E-04	3.96E-02
**REACTOME**	Toll Like Receptor 9 (TLR9) Cascade	1.48E-05	1.13E-04	4.23E-02
**WikiPathways**	Canonical NF-KB	1.65E-05	1.25E-04	4.72E-02
**BioCarta**	roles of arrestin dependent recruitment of src kinases in gpcr signaling	1.69E-05	1.28E-04	4.84E-02
**Panther_Pathway**	PDGF signaling	1.77E-05	1.34E-04	5.07E-02
**REACTOME**	Transfer of LPS from LBP carrier to CD14	1.80E-05	1.35E-04	5.13E-02
**KEGG**	Transcriptional misregulation in cancer	1.79E-05	1.35E-04	5.12E-02
**REACTOME**	Interleukin-6 family signaling	1.82E-05	1.36E-04	5.20E-02
**INOH**	TLR ECSIT MEKK1 p38	1.82E-05	1.36E-04	5.19E-02
**BioCarta**	il22 soluble receptor signaling	1.89E-05	1.41E-04	5.40E-02
**PID**	HIV-1 Nef: Negative effector of Fas and TNF-alpha	1.91E-05	1.42E-04	5.45E-02
**REACTOME**	TRAF6 mediated induction of NFkB and MAP kinases upon TLR7/8 or 9 activation	1.95E-05	1.45E-04	5.59E-02
**stke**	T Cell Signal Transduction	1.99E-05	1.48E-04	5.69E-02
**WikiPathways**	IL-1 signaling	2.02E-05	1.49E-04	5.77E-02
**KEGG**	Rap1 signaling	2.03E-05	1.49E-04	5.80E-02
**REACTOME**	Signaling by PDGF	2.10E-05	1.54E-04	6.00E-02
**WikiPathways**	Human Thyroid Stimulating Hormone (TSH) signaling	2.14E-05	1.57E-04	6.11E-02
**SIGNOR2.0**	SIGNOR-AML	2.17E-05	1.59E-04	6.22E-02
**BioCarta**	tpo signaling	2.25E-05	1.64E-04	6.43E-02

**PID**	Glucocorticoid receptor regulatory network	2.33E-05	1.69E-04	6.66E-02
**ACSN2**	INTEGRINS	2.41E-05	1.74E-04	6.87E-02
**WikiPathways**	Development of pulmonary dendritic cells and macrophage subsets	2.45E-05	1.77E-04	6.99E-02
**Spike**	MAPK signaling	2.48E-05	1.79E-04	7.09E-02
**NetPath**	IL-7	2.58E-05	1.86E-04	7.37E-02
**REACTOME**	IRAK2 mediated activation of TAK1 complex upon TLR7/8 or 9 stimulation	2.65E-05	1.90E-04	7.58E-02
**REACTOME**	TRAF6-mediated induction of TAK1 complex within TLR4 complex	2.65E-05	1.90E-04	7.58E-02
**REACTOME**	p75NTR signals via NF-kB	2.67E-05	1.91E-04	7.63E-02
**BioCarta**	role of egf receptor transactivation by gpcrs in cardiac hypertrophy	2.75E-05	1.96E-04	7.86E-02
**ACSN2**	MARKERS NEUTROPHIL	2.76E-05	1.96E-04	7.88E-02
**REACTOME**	The NLRP3 inflammasome	3.02E-05	2.14E-04	8.64E-02
**INOH**	IL-5 signaling	3.14E-05	2.22E-04	8.98E-02
**PID**	EPHA forward signaling	3.23E-05	2.28E-04	9.22E-02
**INOH**	TLR NFkB	3.30E-05	2.33E-04	9.44E-02
**KEGG**	PI3K-Akt signaling	3.48E-05	2.44E-04	9.94E-02
**REACTOME**	Erythropoietin activates RAS	3.86E-05	2.70E-04	1.10E-01
**NetPath**	CRH	4.02E-05	2.81E-04	1.15E-01
**WikiPathways**	Transcription factor regulation in adipogenesis	4.05E-05	2.82E-04	1.16E-01
**ACSN2**	DEATH RECEPTOR	4.24E-05	2.95E-04	1.21E-01
**WikiPathways**	RAC1/PAK1/p38/MMP2	4.27E-05	2.96E-04	1.22E-01
**BioCarta**	alternative complement	4.34E-05	3.00E-04	1.24E-01
**REACTOME**	Signaling by cytosolic FGFR1 fusion mutants	4.41E-05	3.04E-04	1.26E-01
**PID**	CD40/CD40L signaling	4.65E-05	3.20E-04	1.33E-01
**PharmGKB**	EGFR Inhibitor Pathway Pharmacodynamics	4.91E-05	3.37E-04	1.40E-01
**REACTOME**	Antigen activates B Cell Receptor (BCR) leading to generation of second messengers	5.12E-05	3.51E-04	1.46E-01
**Spike**	Apoptosis Anti-Apoptosis Network	5.32E-05	3.64E-04	1.52E-01
**PID**	Alpha9 beta1 integrin signaling events	5.61E-05	3.82E-04	1.60E-01
**WikiPathways**	TNF alpha Signaling	5.81E-05	3.95E-04	1.66E-01
**WikiPathways**	Oxidative Stress	5.85E-05	3.97E-04	1.67E-01
**Panther_Pathway**	JAK STAT signaling	5.93E-05	4.01E-04	1.69E-01
**ACSN2**	NECROPTOSIS	6.04E-05	4.08E-04	1.73E-01
**PID**	IL2-mediated signaling events	6.12E-05	4.12E-04	1.75E-01
**WikiPathways**	PDGFR-beta	6.14E-05	4.13E-04	1.76E-01
**WikiPathways**	IL17 signaling	6.24E-05	4.18E-04	1.78E-01
**REACTOME**	Class A/1 (Rhodopsin-like receptors)	6.32E-05	4.23E-04	1.81E-01
**REACTOME**	ECM proteoglycans	6.61E-05	4.42E-04	1.89E-01
**NetPath**	AndrogenReceptor	6.66E-05	4.44E-04	1.90E-01
**WikiPathways**	Vitamin B12 Metabolism	7.12E-05	4.73E-04	2.03E-01
**WikiPathways**	Adipogenesis	7.33E-05	4.86E-04	2.09E-01
**REACTOME**	Interleukin-12 family signaling	7.66E-05	5.07E-04	2.19E-01
**WikiPathways**	Prion disease	7.97E-05	5.26E-04	2.28E-01
**KEGG**	Relaxin signaling	8.15E-05	5.37E-04	2.33E-01
**BioCarta**	il 3 signaling	8.19E-05	5.38E-04	2.34E-01
**KEGG**	MAPK signaling	8.82E-05	5.78E-04	2.52E-01
**PID**	Calcineurin-regulated NFAT-dependent transcription in lymphocytes	9.10E-05	5.95E-04	2.60E-01
**NetPath**	TNFalpha	9.20E-05	6.00E-04	2.63E-01
**REACTOME**	Signaling by TGF-beta Receptor Complex in Cancer	9.45E-05	6.15E-04	2.70E-01
**WikiPathways**	PI3K-Akt Signaling	9.58E-05	6.22E-04	2.74E-01
**REACTOME**	Collagen formation	1.01E-04	6.53E-04	2.88E-01
**IPAVS**	ER Stress Map	1.02E-04	6.60E-04	2.92E-01
**REACTOME**	Assembly of collagen fibrils and other multimeric structures	1.04E-04	6.73E-04	2.98E-01
**REACTOME**	Role of phospholipids in phagocytosis	1.04E-04	6.73E-04	2.98E-01
**WikiPathways**	Brain-Derived Neurotrophic Factor (BDNF) signaling	1.11E-04	7.14E-04	3.18E-01
**WikiPathways**	EPO Receptor Signaling	1.12E-04	7.15E-04	3.19E-01
**BioCarta**	ifn gamma signaling	1.13E-04	7.22E-04	3.23E-01
**REACTOME**	Interleukin-20 family signaling	1.14E-04	7.26E-04	3.25E-01
**WikiPathways**	Integrin-mediated Cell Adhesion	1.14E-04	7.27E-04	3.26E-01
**REACTOME**	Interleukin receptor SHC signaling	1.17E-04	7.43E-04	3.35E-01
**KEGG**	Sphingolipid signaling	1.19E-04	7.50E-04	3.39E-01
**INOH**	B cell receptor signaling	1.18E-04	7.50E-04	3.38E-01
**BioCarta**	keratinocyte differentiation	1.20E-04	7.58E-04	3.43E-01
**REACTOME**	PI3K/AKT Signaling in Cancer	1.23E-04	7.75E-04	3.52E-01
**REACTOME**	Other interleukin signaling	1.35E-04	8.50E-04	3.87E-01
**WikiPathways**	Transcriptional cascade regulating adipogenesis	1.36E-04	8.53E-04	3.89E-01
**REACTOME**	MyD88-independent TLR4 cascade	1.42E-04	8.86E-04	4.06E-01

**REACTOME**	TRIF(TICAM1)-mediated TLR4 signaling	1.42E-04	8.86E-04	4.06E-01
**ACSN2**	SENESCENCE	1.42E-04	8.87E-04	4.06E-01
**PID**	Signaling events mediated by TCPTP	1.42E-04	8.87E-04	4.05E-01
**REACTOME**	Loss of Function of SMAD2/3 in Cancer	1.49E-04	9.21E-04	4.24E-01
**WikiPathways**	Corticotropin-releasing hormone signaling	1.53E-04	9.49E-04	4.38E-01
**PID**	Signaling events mediated by Stem cell factor receptor (c-Kit)	1.58E-04	9.74E-04	4.51E-01
**WikiPathways**	Apoptosis-related network due to altered Notch3 in ovarian cancer	1.67E-04	1.03E-03	4.77E-01
**REACTOME**	Free fatty acids regulate insulin secretion	1.72E-04	1.05E-03	4.90E-01
**PID**	Angiopoietin receptor Tie2-mediated signaling	1.73E-04	1.06E-03	4.94E-01
**stke**	Interferon gamma Pathway	1.75E-04	1.07E-03	5.01E-01
**REACTOME**	Collagen biosynthesis and modifying enzymes	1.83E-04	1.11E-03	5.22E-01
**KEGG**	Adherens junction	1.82E-04	1.11E-03	5.21E-01
**PID**	TNF receptor signaling pathway	1.94E-04	1.18E-03	5.56E-01
**IPAVS**	RANK Singnaling Pathway in Osteoclast resorption	1.99E-04	1.21E-03	5.69E-01
**REACTOME**	Scavenging by Class A Receptors	2.01E-04	1.22E-03	5.75E-01
**PharmGKB**	Tacrolimus Cyclosporine Pathway Pharmacodynamics	2.05E-04	1.24E-03	5.86E-01
**REACTOME**	Interleukin-18 signaling	2.10E-04	1.26E-03	6.00E-01
**SIGNOR2.0**	SIGNOR-IL1R	2.12E-04	1.27E-03	6.05E-01
**BioCarta**	inhibition of cellular proliferation by gleevec	2.15E-04	1.29E-03	6.13E-01
**REACTOME**	Axon guidance	2.23E-04	1.34E-03	6.38E-01
**WikiPathways**	The human immune response to tuberculosis	2.26E-04	1.35E-03	6.45E-01
**REACTOME**	Activation of IRF3/IRF7 mediated by TBK1/IKK epsilon	2.32E-04	1.38E-03	6.62E-01
**PID**	Caspase Cascade in Apoptosis	2.41E-04	1.44E-03	6.90E-01
**WikiPathways**	Role Altered Glycolysation of MUC1 in Tumour Microenvironment	2.48E-04	1.48E-03	7.10E-01
**WikiPathways**	Focal Adhesion-PI3K-Akt-mTOR-signaling	2.63E-04	1.56E-03	7.52E-01
**WikiPathways**	EBV LMP1 signaling	2.65E-04	1.57E-03	7.59E-01
**REACTOME**	Diseases of signal transduction	2.90E-04	1.71E-03	8.29E-01
**REACTOME**	TGFBR2 MSI Frameshift Mutants in Cancer	2.92E-04	1.72E-03	8.36E-01
**WikiPathways**	IL-9 Signaling	2.92E-04	1.72E-03	8.36E-01
**INOH**	IFN alpha signaling	3.06E-04	1.80E-03	8.75E-01
**WikiPathways**	Integrated Breast Cancer	3.13E-04	1.83E-03	8.94E-01
**IPAVS**	FAS signaling in cardiac hypertrophy	3.31E-04	1.93E-03	9.45E-01
**NetPath**	IL9	3.39E-04	1.98E-03	9.68E-01
**BioCarta**	il 4 signaling	3.50E-04	2.04E-03	1.00E+00
**BioCarta**	d4gdi signaling	3.59E-04	2.08E-03	1.00E+00
**PID**	Integrins in angiogenesis	3.63E-04	2.10E-03	1.00E+00
**IPAVS**	EGF receptor transactivation in Cardiac Hypertrophy	4.07E-04	2.35E-03	1.00E+00
**WikiPathways**	TGF-beta Signaling	4.11E-04	2.37E-03	1.00E+00
**BioCarta**	hiv-1 nef: negative effector of fas and tnf	4.14E-04	2.38E-03	1.00E+00
**KEGG**	Focal adhesion	4.21E-04	2.42E-03	1.00E+00
**SIGNOR2.0**	SIGNOR-TGFb	4.28E-04	2.45E-03	1.00E+00
**INOH**	IFN gamma signaling	4.27E-04	2.45E-03	1.00E+00
**Panther_Pathway**	Plasminogen activating cascade	4.30E-04	2.46E-03	1.00E+00
**KEGG**	Viral carcinogenesis	4.39E-04	2.50E-03	1.00E+00
**REACTOME**	Class I MHC mediated antigen processing & presentation	4.39E-04	2.50E-03	1.00E+00
**PID**	IFN-gamma	4.55E-04	2.57E-03	1.00E+00
**BioCarta**	how does salmonella hijack a cell	4.55E-04	2.59E-03	1.00E+00
**INOH**	IL-23 signaling	4.55E-04	2.59E-03	1.00E+00
**KEGG**	Lysosome	4.55E-04	2.59E-03	1.00E+00
**BioCarta**	cd40l signaling	4.82E-04	2.72E-03	1.00E+00
**REACTOME**	CLEC7A (Dectin-1) signaling	4.85E-04	2.73E-03	1.00E+00
**BioCarta**	the information processing pathway at the ifn beta enhancer	5.01E-04	2.81E-03	1.00E+00
**WikiPathways**	Mammary gland development pathway - Involution (Stage 4 of 4)	5.02E-04	2.82E-03	1.00E+00
**REACTOME**	CTLA4 inhibitory signaling	5.06E-04	2.83E-03	1.00E+00
**PID**	ErbB1 downstream signaling	5.08E-04	2.84E-03	1.00E+00
**ACSN2**	IMMUNOSTIMULATORY CYTOKINE EXPRESSION	5.12E-04	2.85E-03	1.00E+00
**stke**	Natural Killer Cell Receptor Signaling Pathway	5.20E-04	2.88E-03	1.00E+00
**REACTOME**	RUNX3 Regulates Immune Response and Cell Migration	5.18E-04	2.88E-03	1.00E+00
**WikiPathways**	Eicosanoid Synthesis	5.18E-04	2.88E-03	1.00E+00
**KEGG**	Necroptosis	5.24E-04	2.90E-03	1.00E+00
**NetPath**	Alpha6Beta4Integrin	5.32E-04	2.93E-03	1.00E+00
**PID**	AP-1 transcription factor network	5.55E-04	3.06E-03	1.00E+00
**BioCarta**	pdgf signaling	5.61E-04	3.08E-03	1.00E+00
**REACTOME**	DEx/H-box helicases activate type I IFN and inflammatory cytokines production	5.67E-04	3.11E-03	1.00E+00
**PID**	IL5-mediated signaling events	5.80E-04	3.17E-03	1.00E+00
**REACTOME**	Signaling by FGFR1 in disease	5.80E-04	3.17E-03	1.00E+00
**ACSN2**	CASPASES	5.84E-04	3.19E-03	1.00E+00
**NetPath**	EGFR1	5.87E-04	3.19E-03	1.00E+00
**REACTOME**	Cytosolic sensors of pathogen-associated DNA	5.99E-04	3.25E-03	1.00E+00

**REACTOME**	Downstream signal transduction	6.13E-04	3.32E-03	1.00E+00
**REACTOME**	NF-kB is activated and signals survival	6.19E-04	3.35E-03	1.00E+00
**REACTOME**	DDX58/IFIH1-mediated induction of interferon-alpha/beta	6.34E-04	3.42E-03	1.00E+00
**PID**	Regulation of Androgen receptor activity	6.38E-04	3.44E-03	1.00E+00
**REACTOME**	L1CAM interactions	6.70E-04	3.61E-03	1.00E+00
**REACTOME**	NOD1/2 Signaling Pathway	6.82E-04	3.66E-03	1.00E+00
**WikiPathways**	TGF-beta Receptor Signaling	6.89E-04	3.70E-03	1.00E+00
**REACTOME**	Death Receptor Signalling	7.10E-04	3.80E-03	1.00E+00
**REACTOME**	Synthesis of Lipoxins (LX)	7.12E-04	3.80E-03	1.00E+00
**ACSN2**	CAF INHIBITION ANTITUMOR	7.20E-04	3.84E-03	1.00E+00
**IPAVS**	Alkaptonuria metabolite	7.23E-04	3.85E-03	1.00E+00
**WikiPathways**	MAPK Signaling	7.27E-04	3.86E-03	1.00E+00
**REACTOME**	Loss of Function of TGFBR1 in Cancer	7.34E-04	3.88E-03	1.00E+00
**PID**	HIF-1-alpha transcription factor network	7.33E-04	3.89E-03	1.00E+00
**NetPath**	TWEAK	7.45E-04	3.94E-03	1.00E+00
**WikiPathways**	Regulation of Actin Cytoskeleton	7.50E-04	3.95E-03	1.00E+00
**ACSN2**	APOPTOSIS	7.61E-04	4.01E-03	1.00E+00
**REACTOME**	Collagen chain trimerization	7.68E-04	4.04E-03	1.00E+00
**PharmGKB**	Doxorubicin Pathway Cardiomyocyte Cell Pharmacodynamics	7.76E-04	4.07E-03	1.00E+00
**PID**	RhoA signaling	7.80E-04	4.08E-03	1.00E+00
**WikiPathways**	EGF/EGFR Signaling	7.83E-04	4.09E-03	1.00E+00
**ACSN2**	IMMUNE STIMULATION	7.86E-04	4.10E-03	1.00E+00
**ACSN2**	IMMUNE SUPPRESSION	7.86E-04	4.10E-03	1.00E+00
**ACSN2**	INPUT ACTIVATORS	7.86E-04	4.10E-03	1.00E+00
**ACSN2**	INPUT INHIBITORS	7.86E-04	4.10E-03	1.00E+00
**SignaLink2.0**	JAK/STAT(non-core)	7.99E-04	4.14E-03	1.00E+00
**WikiPathways**	Androgen receptor signaling	8.15E-04	4.21E-03	1.00E+00
**stke**	Tumor Necrosis Factor Pathway	8.32E-04	4.29E-03	1.00E+00
**REACTOME**	Signaling by Erythropoietin	8.35E-04	4.30E-03	1.00E+00
**PID**	TGF-beta receptor signaling	8.77E-04	4.51E-03	1.00E+00
**REACTOME**	Signal amplification	9.08E-04	4.66E-03	1.00E+00
**Spike**	Hearing and Vision Proteins	9.14E-04	4.68E-03	1.00E+00
**KEGG**	HIF-1 signaling	9.25E-04	4.73E-03	1.00E+00
**Panther_Pathway**	5HT2 type receptor mediated signaling	9.28E-04	4.74E-03	1.00E+00
**KEGG**	Adipocytokine signaling	9.35E-04	4.76E-03	1.00E+00
**WikiPathways**	p38 MAPK Signaling	9.35E-04	4.76E-03	1.00E+00
**REACTOME**	Terminal pathway of complement	9.39E-04	4.77E-03	1.00E+00
**BioCarta**	ifn alpha signaling	9.52E-04	4.83E-03	1.00E+00
**REACTOME**	Disease	9.56E-04	4.83E-03	1.00E+00
**REACTOME**	Caspase activation via extrinsic apoptotic signalling	9.55E-04	4.83E-03	1.00E+00
**BioCarta**	growth hormone signaling	9.72E-04	4.90E-03	1.00E+00
**KEGG**	Prolactin signaling	9.78E-04	4.92E-03	1.00E+00
**ACSN2**	TUMOR GROWTH	1.01E-03	5.06E-03	1.00E+00
**SIGNOR2.0**	SIGNOR-TA	1.04E-03	5.23E-03	1.00E+00
**SIGNOR2.0**	SIGNOR-EGF	1.05E-03	5.27E-03	1.00E+00
**REACTOME**	TAK1 activates NFkB by phosphorylation and activation of IKKs complex	1.08E-03	5.40E-03	1.00E+00
**WikiPathways**	Focal Adhesion	1.08E-03	5.41E-03	1.00E+00
**NetPath**	IL11	1.10E-03	5.46E-03	1.00E+00
**KEGG**	Hepatitis C	1.11E-03	5.49E-03	1.00E+00
**NetPath**	TGF beta Receptor	1.13E-03	5.60E-03	1.00E+00
**stke**	Fas Signaling Pathway	1.14E-03	5.62E-03	1.00E+00
**WikiPathways**	Nanoparticle triggered regulated necrosis	1.14E-03	5.63E-03	1.00E+00
**BioCarta**	inactivation of gsk3 by akt causes accumulation of b-catenin in alveolar macrophages	1.15E-03	5.69E-03	1.00E+00
**BioCarta**	ctcf: first multivalent nuclear factor	1.16E-03	5.71E-03	1.00E+00
**stke**	G alpha 13 Pathway	1.17E-03	5.76E-03	1.00E+00
**REACTOME**	Regulation of IFNA signaling	1.21E-03	5.93E-03	1.00E+00
**REACTOME**	TRAF6 mediated IRF7 activation	1.22E-03	5.97E-03	1.00E+00
**REACTOME**	Signaling by GPCR	1.23E-03	6.02E-03	1.00E+00
**REACTOME**	Interleukin-23 signaling	1.37E-03	6.69E-03	1.00E+00
**NetPath**	Gastrin	1.39E-03	6.80E-03	1.00E+00
**BioCarta**	epo signaling	1.45E-03	7.07E-03	1.00E+00
**SIGNOR2.0**	SIGNOR-IOA	1.45E-03	7.08E-03	1.00E+00
**REACTOME**	Non-integrin membrane-ECM interactions	1.48E-03	7.17E-03	1.00E+00
**BioCarta**	mechanism of gene regulation by peroxisome proliferators via ppara	1.48E-03	7.18E-03	1.00E+00
**PID**	Calcium signaling in the CD4+ TCR	1.50E-03	7.25E-03	1.00E+00
**IPAVS**	acute Myeloid leukemia Signaling	1.50E-03	7.26E-03	1.00E+00
**stke**	Interleukin 4 (IL-4) Pathway	1.50E-03	7.26E-03	1.00E+00
**BioCarta**	mapkinase signaling	1.53E-03	7.37E-03	1.00E+00
**Panther_Pathway**	EGF receptor signaling	1.55E-03	7.44E-03	1.00E+00
**REACTOME**	Platelet Aggregation (Plug Formation)	1.55E-03	7.45E-03	1.00E+00
**REACTOME**	SMAD2/3 Phosphorylation Motif Mutants in Cancer	1.56E-03	7.47E-03	1.00E+00

**REACTOME**	TGFBR1 KD Mutants in Cancer	1.56E-03	7.47E-03	1.00E+00
**WikiPathways**	Cell migration and invasion through p75NTR	1.56E-03	7.48E-03	1.00E+00
**REACTOME**	Apoptosis	1.57E-03	7.49E-03	1.00E+00
**REACTOME**	Alternative complement activation	1.58E-03	7.49E-03	1.00E+00
**IPAVS**	ANG II and JAK STAT interactions in mediating cardiac myocyte function	1.58E-03	7.52E-03	1.00E+00
**PID**	S1P3	1.72E-03	8.15E-03	1.00E+00
**Panther_Pathway**	Histamine H1 receptor mediated signaling	1.72E-03	8.16E-03	1.00E+00
**INOH**	IL-13 signaling	1.77E-03	8.37E-03	1.00E+00
**PID**	IL8- and CXCR1-mediated signaling events	1.79E-03	8.44E-03	1.00E+00
**REACTOME**	Fatty Acids bound to GPR40 (FFAR1) regulate insulin secretion	1.83E-03	8.60E-03	1.00E+00
**PID**	PAR4-mediated thrombin signaling events	1.83E-03	8.60E-03	1.00E+00
**ACSN2**	PYROPTOSIS	1.87E-03	8.80E-03	1.00E+00
**BioCarta**	corticosteroids and cardioprotection	1.88E-03	8.82E-03	1.00E+00
**PID**	IL3-mediated signaling events	1.89E-03	8.84E-03	1.00E+00
**WikiPathways**	Supression of HMGB1 mediated inflammation by THBD	1.91E-03	8.91E-03	1.00E+00
**KEGG**	MicroRNAs in cancer	1.92E-03	8.92E-03	1.00E+00
**PharmGKB**	Peginterferon alpha 2a Peginterferon alpha 2b Pathway Hepatocyte Pharmacodynamics	1.91E-03	8.93E-03	1.00E+00
**PID**	SHP2 signaling	1.93E-03	8.96E-03	1.00E+00
**REACTOME**	Cell-Cell communication	1.96E-03	9.11E-03	1.00E+00
**WikiPathways**	Nonalcoholic fatty liver disease	2.01E-03	9.32E-03	1.00E+00
**IPAVS**	non canonical Wnt signaling	2.03E-03	9.37E-03	1.00E+00
**KEGG**	Small cell lung cancer	2.05E-03	9.46E-03	1.00E+00
**PID**	FAS (CD95) signaling	2.07E-03	9.55E-03	1.00E+00
**PID**	Nongenotropic Androgen signaling	2.10E-03	9.65E-03	1.00E+00
**BioCarta**	tgf beta signaling	2.10E-03	9.65E-03	1.00E+00
**BioCarta**	bone remodeling	2.10E-03	9.65E-03	1.00E+00
**PID**	LPA receptor mediated events	2.18E-03	1.00E-02	1.00E+00
**ACSN2**	FERROPTOSIS	2.20E-03	1.00E-02	1.00E+00
**PID**	FOXA2 and FOXA3 transcription factor networks	2.20E-03	1.00E-02	1.00E+00
**REACTOME**	Syndecan interactions	2.21E-03	1.01E-02	1.00E+00
**Panther_Pathway**	Oxytocin receptor mediated signaling	2.25E-03	1.02E-02	1.00E+00
**PID**	IL2 signaling events mediated by PI3K	2.25E-03	1.02E-02	1.00E+00
**WikiPathways**	Osteopontin Signaling	2.27E-03	1.03E-02	1.00E+00
**Spike**	Hearing related SIX1 Interaction	2.28E-03	1.03E-02	1.00E+00
**WikiPathways**	Resistin as a regulator of inflammation	2.32E-03	1.05E-02	1.00E+00
**REACTOME**	FGFR1 mutant receptor activation	2.33E-03	1.05E-02	1.00E+00
**INOH**	IL-7 signaling	2.33E-03	1.05E-02	1.00E+00
**NetPath**	Leptin	2.37E-03	1.07E-02	1.00E+00
**BioCarta**	y branching of actin filaments	2.37E-03	1.07E-02	1.00E+00
**PID**	Plexin-D1 Signaling	2.48E-03	1.11E-02	1.00E+00
**WikiPathways**	miRNA targets in ECM and membrane receptors	2.49E-03	1.11E-02	1.00E+00
**REACTOME**	Non-genomic estrogen signaling	2.50E-03	1.12E-02	1.00E+00
**BioCarta**	pkc-catalyzed phosphorylation of inhibitory phosphoprotein of myosin phosphatase	2.51E-03	1.12E-02	1.00E+00
**WikiPathways**	PI3K/AKT/mTOR - VitD3 Signalling	2.56E-03	1.14E-02	1.00E+00
**PharmGKB**	Statin Pathway Pharmacodynamics	2.57E-03	1.15E-02	1.00E+00
**REACTOME**	CD209 (DC-SIGN) signaling	2.59E-03	1.15E-02	1.00E+00
**REACTOME**	Inflammasomes	2.59E-03	1.15E-02	1.00E+00
**BioCarta**	angiotensin ii mediated activation of jnk pathway via pyk2 dependent signaling	2.63E-03	1.16E-02	1.00E+00
**REACTOME**	Regulation of IFNG signaling	2.67E-03	1.18E-02	1.00E+00
**ACSN2**	CYTOSKELETON POLARITY	2.72E-03	1.20E-02	1.00E+00
**Spike**	Apoptosis In The Ear	2.74E-03	1.21E-02	1.00E+00
**INOH**	EPO signaling	2.78E-03	1.22E-02	1.00E+00
**PID**	Endothelins	2.78E-03	1.22E-02	1.00E+00
**INOH**	IL-2 signaling	2.84E-03	1.25E-02	1.00E+00
**INOH**	IL-1 NFkB	2.85E-03	1.25E-02	1.00E+00
**WikiPathways**	Mammary gland development pathway - Pregnancy and lactation (Stage 3 of 4)	2.90E-03	1.27E-02	1.00E+00
**Panther_Pathway**	Cytoskeletal regulation by Rho GTPase	2.92E-03	1.27E-02	1.00E+00
**OntoCancro**	Expanded.Apoptosis	2.92E-03	1.28E-02	1.00E+00
**PID**	Regulation of Telomerase	2.93E-03	1.28E-02	1.00E+00
**REACTOME**	GPCR downstream signalling	2.97E-03	1.29E-02	1.00E+00
**KEGG**	ECM-receptor interaction	3.00E-03	1.30E-02	1.00E+00
**WikiPathways**	Epithelial to mesenchymal transition in colorectal cancer	3.02E-03	1.31E-02	1.00E+00
**BioCarta**	activation of pkc through g-protein coupled receptors	3.02E-03	1.31E-02	1.00E+00
**WikiPathways**	Evolocumab Mechanism	3.05E-03	1.32E-02	1.00E+00
**WikiPathways**	Proprotein convertase subtilisin/kexin type 9 (PCSK9) mediated LDL receptor degradation	3.05E-03	1.32E-02	1.00E+00

**ACSN2**	MAPK	3.07E-03	1.32E-02	1.00E+00
**WikiPathways**	Statin	3.08E-03	1.33E-02	1.00E+00
**Panther_Pathway**	CCKR signaling map	3.09E-03	1.33E-02	1.00E+00
**PID**	FGF signaling	3.10E-03	1.33E-02	1.00E+00
**REACTOME**	Signaling by Rho GTPases	3.15E-03	1.35E-02	1.00E+00
**KEGG**	Neurotrophin signaling	3.19E-03	1.36E-02	1.00E+00
**PID**	ALK1 signaling events	3.21E-03	1.37E-02	1.00E+00
**KEGG**	Cholesterol metabolism	3.22E-03	1.37E-02	1.00E+00
**BioCarta**	bioactive peptide induced signaling	3.27E-03	1.39E-02	1.00E+00
**REACTOME**	MyD88 cascade initiated on plasma membrane	3.31E-03	1.41E-02	1.00E+00
**REACTOME**	Toll Like Receptor 10 (TLR10) Cascade	3.31E-03	1.41E-02	1.00E+00
**REACTOME**	Toll Like Receptor 5 (TLR5) Cascade	3.31E-03	1.41E-02	1.00E+00
**BioCarta**	p38 mapk signaling	3.33E-03	1.41E-02	1.00E+00
**REACTOME**	IkBA variant leads to EDA-ID	3.40E-03	1.43E-02	1.00E+00
**PID**	LKB1 signaling events	3.40E-03	1.44E-02	1.00E+00
**REACTOME**	TNFs bind their physiological receptors	3.43E-03	1.45E-02	1.00E+00
**REACTOME**	G alpha (q) signalling events	3.45E-03	1.45E-02	1.00E+00
**KEGG**	Lysine degradation	3.48E-03	1.46E-02	1.00E+00
**ACSN2**	CORE	3.48E-03	1.46E-02	1.00E+00
**REACTOME**	PIP3 activates AKT signaling	3.52E-03	1.47E-02	1.00E+00
**BioCarta**	il-2 receptor beta chain in t cell activation	3.61E-03	1.51E-02	1.00E+00
**REACTOME**	Negative regulation of the PI3K/AKT network	3.61E-03	1.51E-02	1.00E+00
**KEGG**	Non-alcoholic fatty liver disease (NAFLD)	3.71E-03	1.54E-02	1.00E+00
**REACTOME**	RUNX3 regulates CDKN1A transcription	3.71E-03	1.55E-02	1.00E+00
**REACTOME**	Growth hormone receptor signaling	3.72E-03	1.55E-02	1.00E+00
**BioCarta**	il 6 signaling	3.79E-03	1.58E-02	1.00E+00
**IPAVS**	Cardiac Hypertrophy	3.80E-03	1.58E-02	1.00E+00
**BioCarta**	role of erbb2 in signal transduction and oncology	3.83E-03	1.59E-02	1.00E+00
**REACTOME**	NCAM1 interactions	3.89E-03	1.60E-02	1.00E+00
**REACTOME**	Loss of Function of TGFBR2 in Cancer	3.88E-03	1.61E-02	1.00E+00
**REACTOME**	TGFBR2 Kinase Domain Mutants in Cancer	3.88E-03	1.61E-02	1.00E+00
**REACTOME**	Elastic fibre formation	3.94E-03	1.62E-02	1.00E+00
**REACTOME**	IRAK4 deficiency (TLR5)	3.93E-03	1.62E-02	1.00E+00
**stke**	Integrin Signaling Pathway	3.97E-03	1.63E-02	1.00E+00
**systems-biology.org**	hepatocyte v2.9	4.00E-03	1.64E-02	1.00E+00
**KEGG**	Bacterial invasion of epithelial cells	4.06E-03	1.66E-02	1.00E+00
**REACTOME**	Interleukin-15 signaling	4.08E-03	1.67E-02	1.00E+00
**BioCarta**	chrebp regulation by carbohydrates and camp	4.09E-03	1.67E-02	1.00E+00
**REACTOME**	Defective CHST3 causes SEDCJD	4.11E-03	1.67E-02	1.00E+00
**BioCarta**	thrombin signaling and protease-activated receptors	4.10E-03	1.67E-02	1.00E+00
**BioCarta**	cadmium induces dna synthesis and proliferation in macrophages	4.14E-03	1.68E-02	1.00E+00
**PID**	EPHA2 forward signaling	4.14E-03	1.68E-02	1.00E+00
**REACTOME**	Interleukin-1 signaling	4.16E-03	1.69E-02	1.00E+00
**PID**	PAR1-mediated thrombin signaling events	4.17E-03	1.69E-02	1.00E+00
**WikiPathways**	Mammary gland development pathway - Puberty (Stage 2 of 4)	4.18E-03	1.69E-02	1.00E+00
**REACTOME**	Defective CHST14 causes EDS, musculocontractural type	4.19E-03	1.69E-02	1.00E+00
**PID**	S1P4	4.21E-03	1.70E-02	1.00E+00
**REACTOME**	Signaling by ERBB4	4.22E-03	1.70E-02	1.00E+00
**REACTOME**	GPCR ligand binding	4.22E-03	1.70E-02	1.00E+00
**PID**	Nectin adhesion	4.23E-03	1.70E-02	1.00E+00
**WikiPathways**	Vitamin D in inflammatory diseases	4.26E-03	1.71E-02	1.00E+00
**REACTOME**	Defective CHSY1 causes TPBS	4.27E-03	1.71E-02	1.00E+00
**WikiPathways**	Physiological and Pathological Hypertrophy of the Heart	4.30E-03	1.72E-02	1.00E+00
**ACSN2**	TELOMERASE	4.34E-03	1.73E-02	1.00E+00
**WikiPathways**	Notch Signaling	4.34E-03	1.73E-02	1.00E+00
**WikiPathways**	Aryl Hydrocarbon Receptor	4.33E-03	1.73E-02	1.00E+00
**PID**	C-MYB transcription factor network	4.36E-03	1.73E-02	1.00E+00
**BioCarta**	ucalpain and friends in cell spread	4.37E-03	1.74E-02	1.00E+00
**PID**	Signaling events mediated by focal adhesion kinase	4.64E-03	1.84E-02	1.00E+00
**REACTOME**	Integrin alphaIIb beta3 signaling	4.65E-03	1.84E-02	1.00E+00
**REACTOME**	Integrin signaling	4.65E-03	1.84E-02	1.00E+00
**systems-biology.org**	adipocyte v2.1	4.69E-03	1.85E-02	1.00E+00
**WikiPathways**	Estrogen signaling	4.72E-03	1.86E-02	1.00E+00
**REACTOME**	Toll Like Receptor 3 (TLR3) Cascade	4.73E-03	1.86E-02	1.00E+00
**REACTOME**	TRAF6 mediated IRF7 activation in TLR7/8 or 9 signaling	4.83E-03	1.90E-02	1.00E+00
**INOH**	IL-3 signaling	4.83E-03	1.90E-02	1.00E+00
**BioCarta**	regulation of transcriptional activity by pml	4.89E-03	1.92E-02	1.00E+00
**KEGG**	Pancreatic cancer	4.95E-03	1.94E-02	1.00E+00
**REACTOME**	EPHB-mediated forward signaling	5.04E-03	1.97E-02	1.00E+00
**INOH**	BMP2 signaling TGF-beta MV	5.24E-03	2.05E-02	1.00E+00
**REACTOME**	Transcriptional activation of mitochondrial biogenesis	5.26E-03	2.05E-02	1.00E+00

**REACTOME**	RUNX1 and FOXP3 control the development of regulatory T lymphocytes (Tregs)	5.27E-03	2.05E-02	1.00E+00
**REACTOME**	Developmental Biology	5.32E-03	2.07E-02	1.00E+00
**BioCarta**	erythropoietin mediated neuroprotection through nf-kb	5.35E-03	2.08E-02	1.00E+00
**REACTOME**	Role of LAT2/NTAL/LAB on calcium mobilization	5.41E-03	2.10E-02	1.00E+00
**PID**	Regulation of retinoblastoma protein	5.57E-03	2.16E-02	1.00E+00
**WikiPathways**	Primary Focal Segmental Glomerulosclerosis FSGS	5.62E-03	2.17E-02	1.00E+00
**BioCarta**	influence of ras and rho proteins on g1 to s transition	5.69E-03	2.19E-02	1.00E+00
**PID**	a4b7 Integrin signaling	5.68E-03	2.20E-02	1.00E+00
**BioCarta**	il-7 signal transduction	5.73E-03	2.21E-02	1.00E+00
**WikiPathways**	Angiogenesis	5.73E-03	2.21E-02	1.00E+00
**WikiPathways**	Metastatic brain tumor	5.80E-03	2.23E-02	1.00E+00
**ACSN2**	MATRIX REGULATION	5.94E-03	2.28E-02	1.00E+00
**WikiPathways**	Extracellular vesicle-mediated signaling in recipient cells	5.97E-03	2.29E-02	1.00E+00
**REACTOME**	Signaling by ROBO receptors	6.13E-03	2.35E-02	1.00E+00
**ACSN2**	MOTILITY	6.17E-03	2.35E-02	1.00E+00
**KEGG**	Prostate cancer	6.17E-03	2.35E-02	1.00E+00
**REACTOME**	PKMTs methylate histone lysines	6.16E-03	2.35E-02	1.00E+00
**BioCarta**	nerve growth factor pathway (ngf)	6.16E-03	2.35E-02	1.00E+00
**SMPDB**	Intracellular Signalling Through Adenosine Receptor A2b and Adenosine	6.21E-03	2.36E-02	1.00E+00
**BioCarta**	erk and pi-3 kinase are necessary for collagen binding in corneal epithelia	6.26E-03	2.37E-02	1.00E+00
**KEGG**	Human papillomavirus infection	6.34E-03	2.40E-02	1.00E+00
**REACTOME**	PPARA activates gene expression	6.42E-03	2.43E-02	1.00E+00
**WikiPathways**	Pancreatic adenocarcinoma	6.47E-03	2.45E-02	1.00E+00
**SMPDB**	Intracellular Signalling Through Adenosine Receptor A2a and Adenosine	6.61E-03	2.50E-02	1.00E+00
**REACTOME**	Localization of the PINCH-ILK-PARVIN complex to focal adhesions	6.67E-03	2.52E-02	1.00E+00
**BioCarta**	#NAME?	6.71E-03	2.53E-02	1.00E+00
**BioCarta**	g-protein signaling through tubby proteins	6.83E-03	2.57E-02	1.00E+00
**REACTOME**	Thrombin signalling through proteinase activated receptors (PARs)	6.90E-03	2.59E-02	1.00E+00
**REACTOME**	TRAF6 mediated NF-kB activation	6.93E-03	2.60E-02	1.00E+00
**Panther_Pathway**	Ras Pathway	6.97E-03	2.61E-02	1.00E+00
**PID**	FOXA1 transcription factor network	7.05E-03	2.64E-02	1.00E+00
**REACTOME**	NCAM signaling for neurite out-growth	7.08E-03	2.64E-02	1.00E+00
**BioCarta**	role of -arrestins in the activation and targeting of map kinases	7.18E-03	2.68E-02	1.00E+00
**KEGG**	Chronic myeloid leukemia	7.31E-03	2.73E-02	1.00E+00
**ACSN2**	RCD GENES	7.33E-03	2.73E-02	1.00E+00
**REACTOME**	Semaphorin interactions	7.38E-03	2.74E-02	1.00E+00
**REACTOME**	Interleukin-12 signaling	7.47E-03	2.77E-02	1.00E+00
**BioCarta**	acetylation and deacetylation of rela in nucleus	7.48E-03	2.77E-02	1.00E+00
**PID**	Validated nuclear estrogen receptor alpha network	7.59E-03	2.81E-02	1.00E+00
**WikiPathways**	Histone Modifications	7.58E-03	2.81E-02	1.00E+00
**Spike**	WNT signaling	7.66E-03	2.83E-02	1.00E+00
**WikiPathways**	Ras Signaling	7.74E-03	2.85E-02	1.00E+00
**Panther_Pathway**	FGF signaling	7.73E-03	2.85E-02	1.00E+00
**Panther_Pathway**	Interferon gamma signaling	7.74E-03	2.85E-02	1.00E+00
**WikiPathways**	Development and heterogeneity of the ILC family	7.80E-03	2.86E-02	1.00E+00
**WikiPathways**	Simplified Interaction Map Between LOXL4 and Oxidative Stress	7.80E-03	2.86E-02	1.00E+00
**REACTOME**	Intrinsic Pathway for Apoptosis	7.80E-03	2.87E-02	1.00E+00
**BioCarta**	tsp-1 induced apoptosis in microvascular endothelial cell	7.96E-03	2.91E-02	1.00E+00
**REACTOME**	Regulated proteolysis of p75NTR	7.99E-03	2.92E-02	1.00E+00
**INOH**	LIF signaling	8.20E-03	2.99E-02	1.00E+00
**WikiPathways**	TFs Regulate miRNAs related to cardiac hypertrophy	8.23E-03	3.00E-02	1.00E+00
**REACTOME**	RIP-mediated NFkB activation via ZBP1	8.26E-03	3.01E-02	1.00E+00
**BioCarta**	stat3 signaling	8.39E-03	3.05E-02	1.00E+00
**Panther_Pathway**	Enkephalin release	8.42E-03	3.06E-02	1.00E+00
**REACTOME**	TGFBR1 LBD Mutants in Cancer	8.42E-03	3.06E-02	1.00E+00
**BioCarta**	rho cell motility signaling	8.46E-03	3.06E-02	1.00E+00
**OntoCancro**	Apoptosis	8.45E-03	3.06E-02	1.00E+00
**KEGG**	Thyroid hormone signaling	8.55E-03	3.09E-02	1.00E+00
**Panther_Pathway**	Axon guidance mediated by Slit Robo	8.68E-03	3.13E-02	1.00E+00
**PID**	Syndecan-2-mediated signaling events	8.92E-03	3.22E-02	1.00E+00
**PID**	Plasma membrane estrogen receptor signaling	9.02E-03	3.25E-02	1.00E+00
**BioCarta**	carm1 and regulation of the estrogen receptor	9.12E-03	3.27E-02	1.00E+00
**WikiPathways**	miRNA regulation of prostate cancer signaling	9.11E-03	3.27E-02	1.00E+00
**BioCarta**	activation of camp-dependent protein kinase pka	9.20E-03	3.30E-02	1.00E+00
**BioCarta**	tnfr1 signaling	9.24E-03	3.31E-02	1.00E+00

**REACTOME**	Regulation of lipid metabolism by Peroxisome proliferator-activated receptor alpha (PPARalpha)	9.29E-03	3.32E-02	1.00E+00
**PID**	Alternative NF-kappaB	9.33E-03	3.33E-02	1.00E+00
**PID**	Signaling events mediated by HDAC Class I	9.52E-03	3.40E-02	1.00E+00
**NetPath**	Ghrelin	9.53E-03	3.40E-02	1.00E+00
**SIGNOR2.0**	SIGNOR-IL6	9.65E-03	3.44E-02	1.00E+00
**REACTOME**	p130Cas linkage to MAPK signaling for integrins	9.69E-03	3.44E-02	1.00E+00
**BioCarta**	rac1 cell motility signaling	9.73E-03	3.46E-02	1.00E+00
**BioCarta**	cxcr4 signaling	9.78E-03	3.47E-02	1.00E+00
**WikiPathways**	Insulin Signaling	9.85E-03	3.49E-02	1.00E+00
**PID**	ErbB2/ErbB3 signaling events	9.99E-03	3.52E-02	1.00E+00
**PID**	Integrin-linked kinase signaling	9.97E-03	3.52E-02	1.00E+00
**WikiPathways**	DNA Damage Response (only ATM dependent)	9.99E-03	3.53E-02	1.00E+00
**BioCarta**	fmlp induced chemokine gene expression in hmc-1 cells	9.97E-03	3.53E-02	1.00E+00
**INOH**	VEGF	1.02E-02	3.61E-02	1.00E+00
**stke**	TGF beta Signaling in Gastrointestinal Stem Cells	1.03E-02	3.61E-02	1.00E+00
**REACTOME**	p75 NTR receptor-mediated signalling	1.06E-02	3.71E-02	1.00E+00
**SMPDB**	Degradation of Superoxides	1.06E-02	3.72E-02	1.00E+00
**REACTOME**	Formation of Fibrin Clot (Clotting Cascade)	1.08E-02	3.78E-02	1.00E+00
**ACSN2**	G2 M CHECKPOINT	1.11E-02	3.89E-02	1.00E+00
**REACTOME**	Signaling by high-kinase activity BRAF mutants	1.12E-02	3.90E-02	1.00E+00
**PID**	CXCR3-mediated signaling events	1.13E-02	3.94E-02	1.00E+00
**PID**	Regulation of RhoA activity	1.14E-02	3.98E-02	1.00E+00
**REACTOME**	Signaling by NODAL	1.14E-02	3.98E-02	1.00E+00
**REACTOME**	Interleukin-37 signaling	1.15E-02	3.99E-02	1.00E+00
**PID**	Ceramide signaling	1.16E-02	4.02E-02	1.00E+00
**OntoCancro**	Non.homologous.end.joining	1.18E-02	4.10E-02	1.00E+00
**OntoCancro**	Mismatch.repair..MMR.	1.19E-02	4.11E-02	1.00E+00
**ACSN2**	CAF	1.19E-02	4.11E-02	1.00E+00
**OntoCancro**	Homologous.recombination..HR.	1.19E-02	4.11E-02	1.00E+00
**OntoCancro**	Nucleotide.excision.repair..NER.	1.19E-02	4.11E-02	1.00E+00
**OntoCancro**	Base.excision.repair..BER.	1.20E-02	4.13E-02	1.00E+00
**BioCarta**	links between pyk2 and map kinases	1.21E-02	4.15E-02	1.00E+00
**PID**	Signaling events regulated by Ret tyrosine kinase	1.21E-02	4.16E-02	1.00E+00
**WikiPathways**	Differentiation of white and brown adipocyte	1.21E-02	4.17E-02	1.00E+00
**ACSN2**	INHIBITING CHECKPOINTS	1.22E-02	4.18E-02	1.00E+00
**OntoCancro**	Chromosome.Stability	1.22E-02	4.19E-02	1.00E+00
**REACTOME**	RUNX1 regulates estrogen receptor mediated transcription	1.25E-02	4.28E-02	1.00E+00
**ACSN2**	HEDGEHOG	1.25E-02	4.29E-02	1.00E+00
**BioCarta**	visceral fat deposits and the metabolic syndrome	1.25E-02	4.29E-02	1.00E+00
**REACTOME**	NOTCH1 Intracellular Domain Regulates Transcription	1.26E-02	4.31E-02	1.00E+00
**NetPath**	BDNF	1.28E-02	4.37E-02	1.00E+00
**PID**	JNK signaling in the CD4+ TCR	1.29E-02	4.39E-02	1.00E+00
**PID**	RXR and RAR heterodimerization with other nuclear receptor	1.30E-02	4.42E-02	1.00E+00
**BioCarta**	pelp1 modulation of estrogen receptor activity	1.30E-02	4.42E-02	1.00E+00
**BioCarta**	egf signaling	1.32E-02	4.47E-02	1.00E+00
**PID**	Validated transcriptional targets of TAp63 isoforms	1.32E-02	4.47E-02	1.00E+00
**OntoCancro**	Cell.Cycle	1.34E-02	4.53E-02	1.00E+00
**WikiPathways**	TCA Cycle Nutrient Utilization and Invasiveness of Ovarian Cancer	1.35E-02	4.54E-02	1.00E+00
**REACTOME**	Recycling pathway of L1	1.35E-02	4.55E-02	1.00E+00
**REACTOME**	Molecules associated with elastic fibres	1.35E-02	4.56E-02	1.00E+00
**PharmGKB**	VEGF Signaling Pathway	1.37E-02	4.59E-02	1.00E+00
**REACTOME**	Regulation of Insulin-like Growth Factor (IGF) transport and uptake by Insulin-like Growth Factor Binding Proteins (IGFBPs)	1.37E-02	4.59E-02	1.00E+00
**REACTOME**	Programmed Cell Death	1.36E-02	4.59E-02	1.00E+00
**PID**	CDC42 signaling events	1.37E-02	4.59E-02	1.00E+00
**PharmGKB**	Beta agonist Beta blocker Pathway Pharmacodynamics	1.37E-02	4.59E-02	1.00E+00
**PID**	PDGFR-alpha signaling	1.38E-02	4.62E-02	1.00E+00
**KEGG**	FoxO signaling	1.38E-02	4.62E-02	1.00E+00
**REACTOME**	ADP signalling through P2Y purinoceptor 1	1.40E-02	4.66E-02	1.00E+00
**IPAVS**	GSK3 as a convergence point in hypertrophic signaling	1.40E-02	4.66E-02	1.00E+00
**stke**	Estrogen Receptor Pathway	1.42E-02	4.73E-02	1.00E+00
**KEGG**	Ras signaling	1.44E-02	4.78E-02	1.00E+00
**UniProt_Pathways**	Metabolic intermediate metabolism; (S)-3-hydroxy-3-methylglutaryl-CoA degradation	1.46E-02	4.84E-02	1.00E+00
**WikiPathways**	Apoptosis Modulation and Signaling	1.47E-02	4.86E-02	1.00E+00
**REACTOME**	Signaling by Non-Receptor Tyrosine Kinases	1.49E-02	4.95E-02	1.00E+00
**REACTOME**	Signaling by PTK6	1.49E-02	4.95E-02	1.00E+00
**KEGG**	Parathyroid hormone synthesis, secretion and action	1.50E-02	4.95E-02	1.00E+00
**systems-biology.org**	Skeletal muscle v2.9	1.49E-02	4.95E-02	1.00E+00

Supplementary Table 4

Gene ontology analysis of differentially regulated genes from sorted tumor cells from tumors of mice on Dox or Ctrl chow as analyzed in Figure 5A. GO analysis according to molecular function, cellular compartments and biological function are shown.

Molecular function

term	p-value	q-value
superoxide-generating NAD(P)H oxidase activity (GO:0016175)	2.77E-07	4.44E-05
oxidoreductase activity, acting on NAD(P)H, oxygen as acceptor (GO:0050664)	1.13E-06	9.03E-05
amyloid-beta binding (GO:0001540)	5.21E-06	0.000278
G protein-coupled chemoattractant receptor activity (GO:0001637)	0.000104	0.004005
cytokine receptor activity (GO:0004896)	0.000125	0.004005
C-C chemokine receptor activity (GO:0016493)	0.000265	0.006024
complement component C3b binding (GO:0001851)	0.000297	0.006024
C-C chemokine binding (GO:0019957)	0.000301	0.006024
chemokine receptor activity (GO:0004950)	0.000384	0.006823
chemokine binding (GO:0019956)	0.000715	0.011437
superoxide-generating NADPH oxidase activator activity (GO:0016176)	0.000821	0.011947
leucine zipper domain binding (GO:0043522)	0.001311	0.017475
LRR domain binding (GO:0030275)	0.002613	0.030048
chemokine receptor binding (GO:0042379)	0.002629	0.030048

Cellular compartment

term	p-value	q-value
phagocytic vesicle (GO:0045335)	1.91E-10	2.10E-08
tertiary granule membrane (GO:0070821)	6.46E-09	3.56E-07
secretory granule membrane (GO:0030667)	3.67E-08	1.04E-06
specific granule membrane (GO:0035579)	3.77E-08	1.04E-06
endocytic vesicle (GO:0030139)	9.26E-08	2.04E-06
tertiary granule (GO:0070820)	3.08E-07	5.64E-06
NADPH oxidase complex (GO:0043020)	4.14E-07	6.51E-06
specific granule (GO:0042581)	2.89E-06	3.97E-05
endocytic vesicle membrane (GO:0030666)	2.71E-05	0.000331
actin filament (GO:0005884)	4.79E-05	0.000527
phagolysosome (GO:0032010)	0.000297	0.002965
cytoplasmic vesicle membrane (GO:0030659)	0.0012	0.010152
collagen-containing extracellular matrix (GO:0062023)	0.0012	0.010152
phagocytic vesicle membrane (GO:0030670)	0.001942	0.015257
secondary lysosome (GO:0005767)	0.00342	0.025083
ficolin-1-rich granule membrane (GO:0101003)	0.004621	0.031773
endocytic vesicle lumen (GO:0071682)	0.00588	0.036026
integral component of plasma membrane (GO:0005887)	0.005895	0.036026

Biological process

term	p-value	q-value
synapse pruning (GO:0098883)	2.00E-12	2.60E-09
microglial cell activation (GO:0001774)	1.67E-09	1.09E-06
regulation of microglial cell mediated cytotoxicity (GO:1904149)	4.31E-09	1.47E-06
positive regulation of cytokine production (GO:0001819)	4.51E-09	1.47E-06
innate immune response (GO:0045087)	1.17E-08	2.79E-06
cell junction disassembly (GO:0150146)	1.29E-08	2.79E-06
neutrophil degranulation (GO:0043312)	4.00E-07	6.19E-05
positive regulation of myeloid leukocyte mediated immunity (GO:0002888)	4.14E-07	6.19E-05
neutrophil activation involved in immune response (GO:0002283)	4.42E-07	6.19E-05
neutrophil mediated immunity (GO:0002446)	4.76E-07	6.19E-05
positive regulation of superoxide anion generation (GO:0032930)	8.31E-07	9.83E-05
regulation of superoxide anion generation (GO:0032928)	1.13E-06	0.000113
superoxide anion generation (GO:0042554)	1.13E-06	0.000113
macrophage activation (GO:0042116)	1.51E-06	0.000131
regulation of hippocampal neuron apoptotic process (GO:0110089)	1.61E-06	0.000131
regulation of neutrophil degranulation (GO:0043313)	1.61E-06	0.000131
phagocytosis, engulfment (GO:0006911)	1.74E-06	0.000133
negative regulation of myeloid leukocyte mediated immunity (GO:0002887)	3.20E-06	0.000231
positive regulation of interleukin-6 production (GO:0032755)	3.86E-06	0.000264
positive regulation of tumor necrosis factor production (GO:0032760)	4.17E-06	0.000271
inflammatory response (GO:0006954)	5.10E-06	0.000316
regulation of tumor necrosis factor production (GO:0032680)	5.57E-06	0.000317
positive regulation of tumor necrosis factor superfamily cytokine production (GO:1903557)	5.60E-06	0.000317
positive regulation of neuron death (GO:1901216)	5.86E-06	0.000318
regulation of interleukin-2 production (GO:0032663)	6.51E-06	0.000339
positive regulation of reactive oxygen species metabolic process (GO:2000379)	7.22E-06	0.000361
regulation of complement activation (GO:0030449)	7.99E-06	0.000385
regulation of immune effector process (GO:0002697)	1.07E-05	0.000479
positive regulation of phagocytosis (GO:0050766)	1.07E-05	0.000479
regulation of humoral immune response (GO:0002920)	1.17E-05	0.000508
complement activation, classical pathway (GO:0006958)	1.33E-05	0.000557
receptor-mediated endocytosis (GO:0006898)	1.42E-05	0.000577
regulation of phagocytosis (GO:0050764)	1.67E-05	0.000658
cellular response to chemical stress (GO:0062197)	2.00E-05	0.000766
plasma membrane invagination (GO:0099024)	2.13E-05	0.00079
humoral immune response mediated by circulating immunoglobulin (GO:0002455)	2.59E-05	0.000935
superoxide metabolic process (GO:0006801)	2.77E-05	0.000973
regulation of interleukin-6 production (GO:0032675)	3.25E-05	0.001112
positive regulation of immune response (GO:0050778)	5.84E-05	0.001947
cellular response to oxidative stress (GO:0034599)	6.66E-05	0.002165

antigen processing and presentation of exogenous peptide antigen via MHC class I (GO:0042590)	7.04E-05	0.002235
response to interferon-gamma (GO:0034341)	7.95E-05	0.002463
positive regulation of leukocyte mediated cytotoxicity (GO:0001912)	9.06E-05	0.002741
regulation of interferon-gamma production (GO:0032649)	0.000112	0.003245
regulation of neuron death (GO:1901214)	0.000112	0.003245
toll-like receptor signaling pathway (GO:0002224)	0.000147	0.003987
positive regulation of monocyte chemotaxis (GO:0090026)	0.000147	0.003987
positive regulation of neutrophil chemotaxis (GO:0090023)	0.000147	0.003987
hemopoiesis (GO:0030097)	0.000171	0.004485
toll-like receptor 4 signaling pathway (GO:0034142)	0.000172	0.004485
regulation of cytokine production (GO:0001817)	0.000182	0.004633
positive regulation of leukocyte chemotaxis (GO:0002690)	0.000222	0.005272
negative regulation of biomineral tissue development (GO:0070168)	0.000231	0.005272
negative regulation of interleukin-2 production (GO:0032703)	0.000231	0.005272
regulation of platelet activation (GO:0010543)	0.000231	0.005272
positive regulation of granulocyte chemotaxis (GO:0071624)	0.000231	0.005272
positive regulation of neutrophil migration (GO:1902624)	0.000231	0.005272
negative regulation of histone H4 acetylation (GO:0090241)	0.000297	0.006641
positive regulation of neuron apoptotic process (GO:0043525)	0.000301	0.006641
cellular response to cytokine stimulus (GO:0071345)	0.000329	0.007123
regulation of neutrophil chemotaxis (GO:0090022)	0.000341	0.007153
positive regulation of T cell migration (GO:2000406)	0.000341	0.007153
regulation of monocyte chemotaxis (GO:0090025)	0.000384	0.007802
positive regulation of interleukin-2 production (GO:0032743)	0.000384	0.007802
regulation of vascular endothelial growth factor production (GO:0010574)	0.00043	0.008608
regulation of type 2 immune response (GO:0002828)	0.000443	0.008736
positive regulation of cell death (GO:0010942)	0.00048	0.009325
vascular endothelial growth factor receptor signaling pathway (GO:0048010)	0.000509	0.00973
positive regulation of receptor catabolic process (GO:2000646)	0.000618	0.011302
regulation of MHC class I biosynthetic process (GO:0045343)	0.000618	0.011302
regulation of dopamine metabolic process (GO:0042053)	0.000618	0.011302
cytokine-mediated signaling pathway (GO:0019221)	0.000625	0.011302
positive regulation of mononuclear cell migration (GO:0071677)	0.00065	0.011592
antigen processing and presentation of exogenous peptide antigen via MHC class I, TAP-dependent (GO:0002479)	0.000704	0.012097
regulation of cellular biosynthetic process (GO:0031326)	0.000715	0.012097
hematopoietic progenitor cell differentiation (GO:0002244)	0.000715	0.012097
negative regulation of macromolecule metabolic process (GO:0010605)	0.000716	0.012097
positive regulation of transcription, DNA-templated (GO:0045893)	0.000738	0.012302
integrin-mediated signaling pathway (GO:0007229)	0.00078	0.012737
positive regulation of cytokine production involved in immune response (GO:0002720)	0.000783	0.012737
positive regulation of isomerase activity (GO:0010912)	0.000821	0.012983
negative regulation of long-term synaptic potentiation (GO:1900272)	0.000821	0.012983

regulation of gene expression (GO:0010468)	0.000828	0.012983
regulation of lipid biosynthetic process (GO:0046890)	0.000932	0.014266
positive regulation of lymphocyte activation (GO:0051251)	0.000932	0.014266
regulation of interleukin-8 production (GO:0032677)	0.001041	0.015044
regulation of T-helper 1 type immune response (GO:0002825)	0.001052	0.015044
regulation of respiratory burst (GO:0060263)	0.001052	0.015044
positive regulation of macrophage cytokine production (GO:0060907)	0.001052	0.015044
negative regulation of leukocyte degranulation (GO:0043301)	0.001052	0.015044
regulation of histone H4 acetylation (GO:0090239)	0.001052	0.015044
regulation of defense response (GO:0031347)	0.00114	0.01612
positive regulation of cell development (GO:0010720)	0.001187	0.016429
positive regulation of production of molecular mediator of immune response (GO:0002702)	0.001187	0.016429
negative regulation of programmed cell death (GO:0043069)	0.00122	0.016708
immunoglobulin mediated immune response (GO:0016064)	0.001311	0.017223
neuron remodeling (GO:0016322)	0.001311	0.017223
defense response to tumor cell (GO:0002357)	0.001311	0.017223
positive regulation of cell activation (GO:0050867)	0.001311	0.017223
negative regulation of apoptotic process (GO:0043066)	0.001436	0.018541
second-messenger-mediated signaling (GO:0019932)	0.001477	0.018541
cortical cytoskeleton organization (GO:0030865)	0.001482	0.018541
T cell differentiation (GO:0030217)	0.001482	0.018541
visual system development (GO:0150063)	0.001482	0.018541
B cell mediated immunity (GO:0019724)	0.001596	0.019493
T cell chemotaxis (GO:0010818)	0.001596	0.019493
positive regulation of transport (GO:0051050)	0.001603	0.019493
positive regulation of NF-kappaB transcription factor activity (GO:0051092)	0.001669	0.020102
regulation of myoblast fusion (GO:1901739)	0.001908	0.022368
positive regulation of T-helper 1 type immune response (GO:0002827)	0.001908	0.022368
negative regulation of histone acetylation (GO:0035067)	0.001908	0.022368
positive regulation of chemotaxis (GO:0050921)	0.001942	0.022556
negative regulation of gene expression (GO:0010629)	0.002031	0.023388
positive regulation of nucleic acid-templated transcription (GO:1903508)	0.00205	0.023396
regulation of B cell proliferation (GO:0030888)	0.002069	0.023406
antifungal innate immune response (GO:0061760)	0.002247	0.02457
positive regulation of adaptive immune response based on somatic recombination of immune receptors built from immunoglobulin superfamily domains (GO:0002824)	0.002247	0.02457
macrophage activation involved in immune response (GO:0002281)	0.002247	0.02457
negative regulation of alcohol biosynthetic process (GO:1902931)	0.002247	0.02457
regulation of interleukin-10 production (GO:0032653)	0.002339	0.025355
calcium-mediated signaling (GO:0019722)	0.002434	0.026172
cellular defense response (GO:0006968)	0.002481	0.02628
negative regulation of bone mineralization (GO:0030502)	0.002613	0.02628
cellular response to low-density lipoprotein particle stimulus (GO:0071404)	0.002613	0.02628

negative regulation of T cell mediated immunity (GO:0002710)	0.002613	0.02628
regulation of macrophage cytokine production (GO:0010935)	0.002613	0.02628
positive regulation of leukocyte degranulation (GO:0043302)	0.002613	0.02628
positive regulation of T cell chemotaxis (GO:0010820)	0.002613	0.02628
positive regulation of type 2 immune response (GO:0002830)	0.002613	0.02628
positive regulation of ERK1 and ERK2 cascade (GO:0070374)	0.002626	0.02628
positive regulation of osteoclast differentiation (GO:0045672)	0.003004	0.029162
regulation of T cell chemotaxis (GO:0010819)	0.003004	0.029162
negative regulation of leukocyte mediated cytotoxicity (GO:0001911)	0.003004	0.029162
positive regulation of cell killing (GO:0031343)	0.003004	0.029162
positive regulation of chemokine production (GO:0032722)	0.003105	0.029822
regulation of immune response (GO:0050776)	0.003117	0.029822
positive regulation of intracellular signal transduction (GO:1902533)	0.003191	0.0303
negative regulation of cytokine production (GO:0001818)	0.003347	0.031119
positive regulation of syncytium formation by plasma membrane fusion (GO:0060143)	0.00342	0.031119
positive regulation of alpha-beta T cell proliferation (GO:0046641)	0.00342	0.031119
positive regulation of myeloid leukocyte cytokine production involved in immune response (GO:0061081)	0.00342	0.031119
neutrophil activation (GO:0042119)	0.00342	0.031119
dendritic cell chemotaxis (GO:0002407)	0.00342	0.031119
chemokine-mediated signaling pathway (GO:0070098)	0.003631	0.032804
positive regulation of cell migration (GO:0030335)	0.003738	0.033535
positive regulation of interferon-gamma production (GO:0032729)	0.003817	0.033955
ruffle organization (GO:0031529)	0.003863	0.033955
positive regulation of smooth muscle cell migration (GO:0014911)	0.003863	0.033955
positive regulation of MAPK cascade (GO:0043410)	0.004088	0.035693
negative regulation of nucleic acid-templated transcription (GO:1903507)	0.004118	0.035718
positive regulation of lymphocyte chemotaxis (GO:0140131)	0.00433	0.036819
neuron maturation (GO:0042551)	0.00433	0.036819
dendritic cell migration (GO:0036336)	0.00433	0.036819
cellular response to chemokine (GO:1990869)	0.004412	0.03727
cellular response to interferon-gamma (GO:0071346)	0.004494	0.037724
positive regulation of interleukin-8 production (GO:0032757)	0.004621	0.038296
pattern recognition receptor signaling pathway (GO:0002221)	0.004621	0.038296
regulation of T cell cytokine production (GO:0002724)	0.004822	0.039089
myeloid cell activation involved in immune response (GO:0002275)	0.004822	0.039089
Rac protein signal transduction (GO:0016601)	0.004822	0.039089
I-kappaB kinase/NF-kappaB signaling (GO:0007249)	0.004837	0.039089
synapse organization (GO:0050808)	0.005186	0.041646
lipopolysaccharide-mediated signaling pathway (GO:0031663)	0.005339	0.042095
positive regulation of alpha-beta T cell activation (GO:0046635)	0.005339	0.042095
cortical actin cytoskeleton organization (GO:0030866)	0.005339	0.042095
positive regulation of T cell proliferation (GO:0042102)	0.005761	0.045149
purinergic nucleotide receptor signaling pathway (GO:0035590)	0.00588	0.045531

positive regulation of lipid storage (GO:0010884)	0.00588	0.045531
negative regulation of transcription, DNA-templated (GO:0045892)	0.005918	0.04556
negative regulation of B cell activation (GO:0050869)	0.006444	0.04903
natural killer cell mediated cytotoxicity (GO:0042267)	0.006444	0.04903
cell chemotaxis (GO:0060326)	0.006518	0.049302
